# A Non‐Antigenic Randomized Polyethylene Glycol/Poly(2‐Phenyl‐2‐Oxazine)‐Based Drug Delivery Platform

**DOI:** 10.1002/marc.202500781

**Published:** 2025-12-24

**Authors:** Julian Schmidt, Anna‐Lena Ziegler, Florian T. Kaps, Laura J. Rosenberger, Matthias Bros, Holger Frey, Robert Luxenhofer

**Affiliations:** ^1^ Department of Chemistry Johannes Gutenberg University Mainz Mainz Germany; ^2^ Soft Matter Chemistry Department of Chemistry and Helsinki Institute of Sustainability Science Faculty of Science University of Helsinki Helsinki Finland; ^3^ Department of Dermatology University Medical Center of the Johannes Gutenberg University Mainz Mainz Germany

**Keywords:** ABA‐triblock copolymer, anti‐PEG antibodies, drug delivery, PEG‐alternatives, polymeric micelles, POx/POzi, rPEG

## Abstract

Polyethylene glycol (PEG) plays a central role in nanomedicine, providing essential properties such as the stealth effect. However, the emergence of anti‐PEG antibodies (APAs) increasingly undermines these benefits, raising concerns about safety and efficiency. This creates an urgent need for PEG alternatives. Randomized PEG (rPEG) represents a conceptually new strategy that preserves the PEG‐like structure and performance while markedly reducing antigenicity. In this work, rPEG is employed as a non‐antigenic A‐block in ABA‐type polymeric micelles (PMs). To enhance drug loading capacity beyond conventional PMs, the hydrophobic middle block is composed of poly(2‐phenyl‐2‐oxazine) (PPheOzi). rPEG is synthesized by anionic ring‐opening polymerization, PPheOzi by cationic ring‐opening polymerization, and the combined rPEG‐*b*‐PPheOzi‐*b*‐rPEG triblock copolymers are linked via copper‐catalyzed azide‐alkyne cycloaddition. The formulations exhibit a distinct correlation between the solubilization of Efavirenz and the systematically varied rPEG composition, ranging from outstanding to moderate micelle drug loading capacities. A fundamental preclinical safety profile was established through investigations in murine fibroblasts and human peripheral blood mononuclear cells (PBMCs). Competitive enzyme‐linked immunosorbent assays (ELISA) revealed a pronounced reduction in APA affinity in comparison to PEG. Taken together, the synergistic combination of rPEG and PPheOzi establishes a non‐antigenic micellar platform capable of achieving high drug loadings.

## Graphical abstract

Randomized PEG (rPEG), a conceptually new PEG alternative, is combined with poly(2‐phenyl‐2‐oxazine) (PPheOzi) to establish an rPEG‐*b*‐PPheOzi‐*b*‐rPEG micellar drug delivery platform. The PPheOzi block enables high drug loadings, while competitive ELISA reveals a drastic reduction in anti‐PEG antibody affinity of rPEG in comparison to PEG. Positioning the synergistic approach as a promising candidate for advanced nanomedicine development.

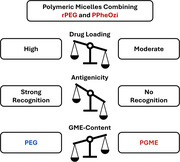

## 1 Introduction

Poly(ethylene glycol) (PEG) is an essential building block in modern nanomedicine [1, 2]. The covalent conjugation of PEG to proteins, small molecules, lipids, or antibodies is referred to as PEGylation, which is one of the key methodologies to increase aqueous solubility [1, 3]. For decades, it has been established that PEGylation significantly increases the circulation time and stability of pharmaceuticals [1, 2, 3, 4, 5, 6]. This is commonly attributed to the “stealth effect” of the nonionic PEG, which has been linked to hydrophilic shielding [7]. Furthermore, investigations of PEGylated nanocarriers revealed that PEG also modulates the composition of the protein corona. Subsequently, the reduced cell uptake of PEGylated nanoparticles has been attributed to the formation of the protein corona [8, 9, 10]. With 38 FDA‐approved PEGylated therapeutics and more than 80 ongoing clinical trials as of May 2023, PEGylation has become an indispensable platform in modern nanomedical applications [1].

In contradiction to the previous scientific consensus that PEG is immunologically inert, it is now well established that the immune system can develop anti‐PEG antibodies (APA) [11, 12]. A concerning increase in APAs in the general population is well‐documented, significantly boosted by the PEGylated lipid nanoparticle (LNP) used in the vaccinations during the SARS‐CoV‐2 pandemic [13, 14, 15]. The population in a typical Western society has an APA‐prevalence of up to 83% [16]. Other studies also detected APA in healthy adults who had never received PEGylated therapeutics. This can be attributed to daily exposure to PEG in cosmetics and food [17, 18]. The ubiquitous prevalence of APAs can lead to accelerated blood clearance (ABC) as well as to hypersensitivity reactions for PEGylated drugs or nanocarriers [12]. The exact mechanism of hypersensitivity remains uncertain [12, 19]. In general, a complement activation‐related pseudoallergy (CARPA) is supposed. In severe cases, the occurrence of anaphylaxis has been reported [19, 20]. The growing body of evidence on PEG immunogenicity has led to concerns about the safety and efficiency of PEGylation. This, in turn, has stimulated a renewed search for PEG alternatives in recent years. Consequently, several biocompatible PEG alternatives based on non‐polyether polymer classes are under investigation, including poly(2‐oxazoline)s (POx), poly(2‐oxazine)s (POzi), polysarcosines, and polymethacrylates with oligo(ethylene glycol) side chains, among others [7, 21, 22, 23, 24]. However, it is essential to note that recent, independent studies have found evidence that some PEG‐alternatives can also induce antibody formation and an ABC effect [25, 26, 27].

We recently introduced the randomized PEG (rPEG) technology as a fundamentally different concept of “PEG isomerization” to target the “PEG dilemma” [28]. The rPEG technology preserves the polyether backbone and introduces statistically distributed side chains by random copolymerization of ethylene oxide (EO) and glycidyl methyl ether (GME). The incorporation of GME disrupts the regularity of the PEG structure, which is the epitope for recognition by APAs [28, 29, 30, 31]. The results clearly show that rPEG retains the beneficial characteristics of PEG while avoiding recognition by anti‐PEG antibodies in a pharmaceutically relevant concentration range [28, 32]. As rPEG is designed as a “drop‐in” replacement for PEG, it is necessary to integrate rPEG into several research fields related to PEGylated pharmaceutical materials.

Polymeric micelles (PMs) constitute a conceptually simple and versatile drug delivery system, typically formed via the self‐assembly of amphiphilic AB or ABA block copolymers [33, 34, 35, 36]. They are employed in clinical applications as excipients or surfactants in nanoparticle formulations as solubilizing agents for poorly water‐soluble drugs [36]. For example, Genexol‐PM is a mPEG‐*b*‐PDLLA‐based system used for the solubilization of Paclitaxel [34, 37]. However, micelles usually have only moderate loading capacities [33, 34, 35, 38]. Despite the concerns regarding immunogenicity, PEG remains the only clinically approved shell‐forming polymer in polymeric micelles (PM) to date [34]. Poloxamers, amphiphilic ABA PEG‐*b*‐PPO‐*b*‐PEG triblock copolymers, are an example of a widely used PEG‐based excipient [35, 37]. However, Poloxamers also induce a significant boost of the antibody levels of anti‐PEG IgG and IgM, which consequently leads to a strong ABC effect for PEGylated nanoparticles [39].

Recent studies on POx/POzi‐based PM systems are challenging the commonly moderate loading capacities of established systems. It was shown that these systems can achieve significantly higher drug loadings [40, 41, 42]. Contrary to the long‐standing view that the hydrophilic corona merely stabilizes the micelle while the hydrophobic core dictates drug loading, several studies have revealed that variations in the hydrophilic shell critically influence both system stability and the solubilization of hydrophobic drugs [43, 44, 45, 46]. However, antibody formation and the ABC effect have already been reported for poly(2‐methyloxazoline) (PMeOx) and poly(2‐ethyloxazoline) (PEtOx), which are used as hydrophilic building blocks in PEG‐free liposomes and LNPs, respectively [25, 26].

Herein, we present the use of rPEG in polymeric micelles. Instead of employing a polyether‐based hydrophobic block, we used the versatile chemistry of POx/POzi to introduce a poly(2‐phenyl‐2‐oxazine) (PPheOzi) middle block. Driven by the need to establish PEG alternatives, the synergistic integration of rPEG and PPheOzi is a promising concept for a non‐antigenic drug delivery system with improved loading capacities.

**FIGURE 1:**
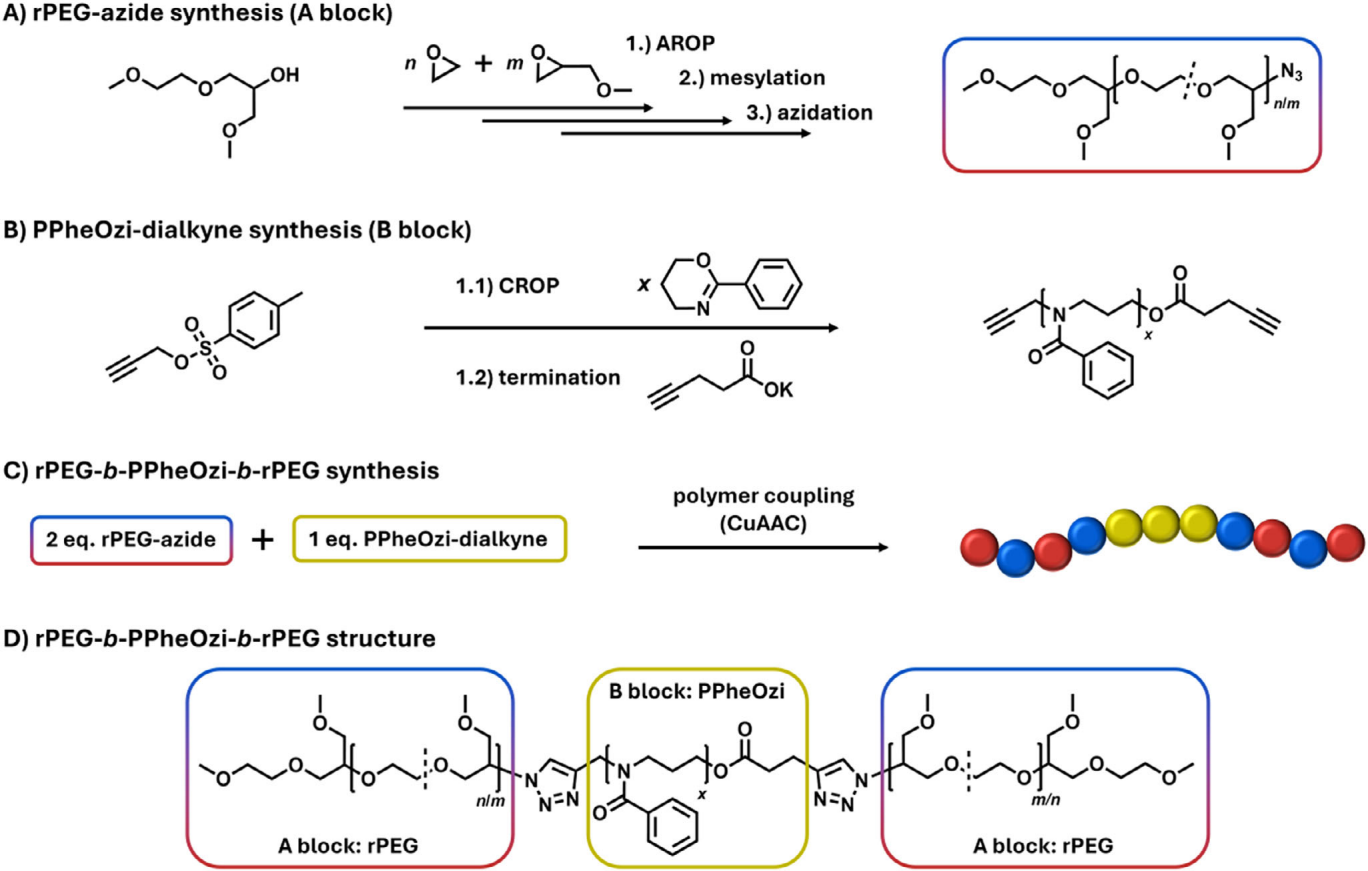
Multi‐step reaction scheme of this work. (A) Synthesis of rPEG‐azide via anionic ring‐opening copolymerization (AROP) of ethylene oxide (EO) and glycidyl methyl ether (GME) with subsequent end group mesylation and azidation. (B) Synthesis of PPheOzi‐dialkyne via cationic ring‐opening polymerization (CROP) of 2‐phenyl‐2‐oxazine employing alkyne‐functionalized initiator and terminating agent. (C) ABA triblock copolymer synthesis via copper‐catalyzed azide‐alkyne cycloaddition (CuAAC). (D) Chemical structure of the ABA triblock copolymer (rPEG‐b‐PPheOzi‐b‐rPEG).

This study investigates the impact of varying GME content in different rPEGs on the drug‐loading capacity and antigenicity of polymeric micelles employing a hydrophobic PPheOzi core. To combine these two complementary synthesized polymer classes, post‐polymerization coupling reactions, such as copper‐catalyzed azide‐alkyne cycloaddition (CuAAC), are necessary (Figure 1).

## 2 Results and Discussion

### 2.1 ABA Triblock Copolymer Synthesis

**FIGURE 2:**
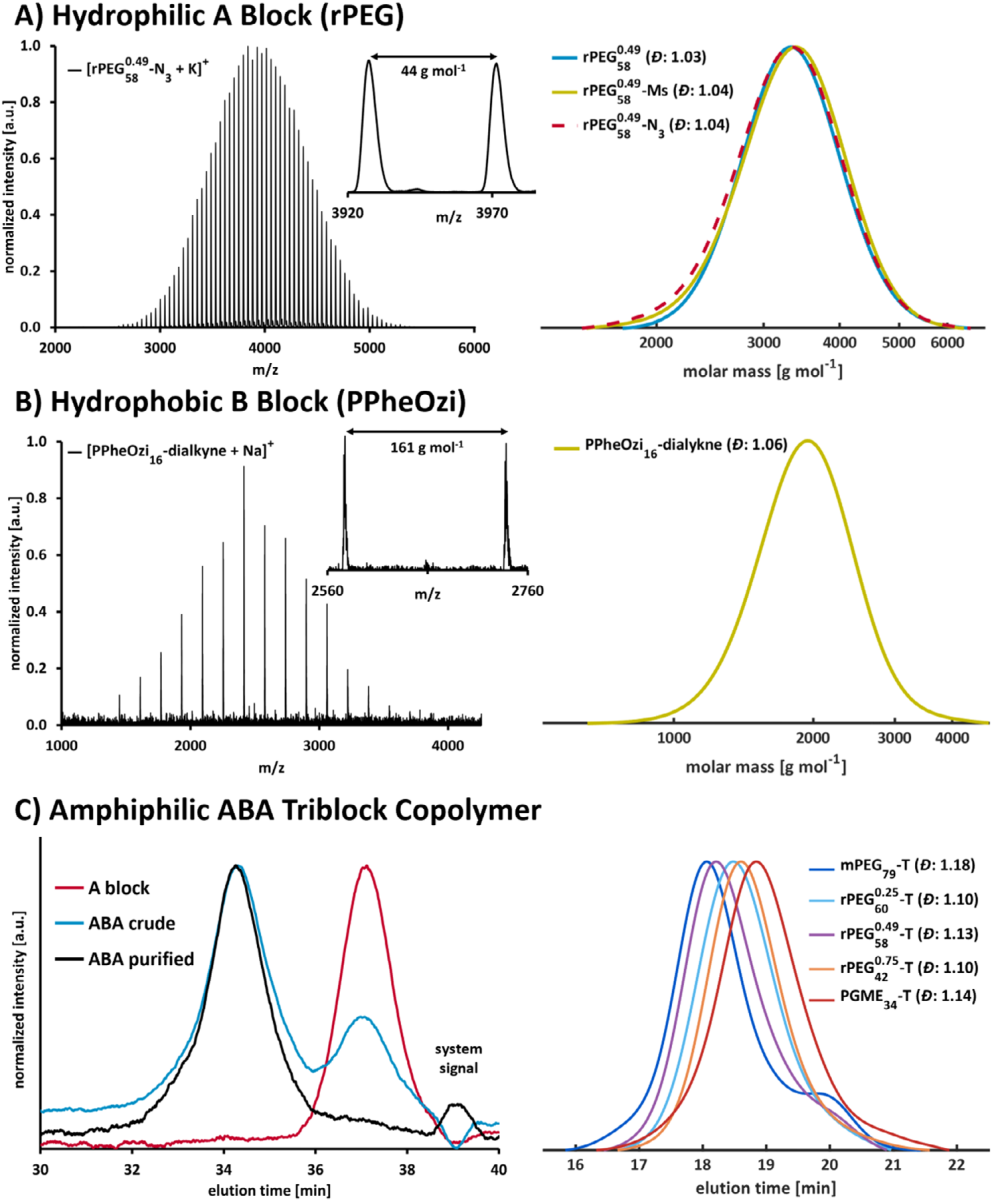
(A) Left: MALDI‐TOF MS of ${\mathrm{rPEG}}_{58}^{0.49}$‐N_3_. Right: Overlayed SEC traces of ${\mathrm{rPEG}}_{58}^{0.49}$ (blue), ${\mathrm{rPEG}}_{58}^{0.49}$‐Ms (yellow) and ${\mathrm{rPEG}}_{58}^{0.49}$‐N_3_ (red). (B) Left: MALDI‐TOF MS and SEC trace (right) of PPheOzi_16_. (C) Left: SEC traces of the purification process of the PGME_34_‐Τ triblock copolymer: Hydrophilic starting material A block (red, PGME_34_‐N_3_), the crude ABA triblock copolymer (blue), and the purified ABA triblock copolymer (black, PGME_34_‐b‐PPheOzi_16_‐b‐PGME_34_). Right: SEC traces of all synthesized ABA triblock copolymers after purification.

The preparation of polyether/POzi‐based triblock copolymers via CuAAC requires azide‐functionalized polyethers, which are synthesized in a three‐step process. To this end, first, the randomized PEGs (rPEGs) and poly(glycidyl methyl ether) (PGME) were synthesized by statistical copolymerization of ethylene oxide (EO) and glycidyl methyl ether (GME) using anionic ring‐opening polymerization (AROP), targeting a molar mass of 3500 g mol^−1^ and GME contents ranging from 25 mol% to 100 mol% (PGME homopolymer) to ensure comparability. After polymerization, the primary and secondary hydroxyl ω‐end groups were mesylated. Subsequently, the azide end groups were introduced by nucleophilic substitution. The average yields of the azidation reaction were successfully increased to above 80% by optimizing the purification procedure. Previous protocols relied on water‐ and brine‐based extractions, which resulted in considerable material losses [32]. This limitation can be effectively addressed by replacing the extraction medium with a 333 g L^−1^ aqueous ammonium sulfate solution. All polymers were characterized by SEC, NMR spectroscopy, and MALDI‐TOF‐MS (Figures S1–S13 and S20–S33 and S35–S39). The rPEG‐ and PGME‐azides were obtained with narrow molar mass distributions (dispersity *Ð* ≤ 1.10), and molar masses ranging from 3000 to 3900 g mol^−1^ (Figure 2A; Table 1). MALDI‐TOF‐MS analysis showed for all rPEG‐azides a single distribution (Figure 2A; Figures S28–S33). The good match between the experimental *m*/*z* values and the calculated *m*/*z* values for the azide end group indicates successful introduction of the azide functionality (e.g., exp.: *m*/*z* = 3928 vs calc.: *m*/*z* = 3929) (Figure S31). FTIR spectroscopy provides further proof of the azide end group modification (Figures S43–S47).

**TABLE 1:** Molecular parameters of mPEG‐, rPEG‐, and PGME‐azides‐ as well as PPheOzi‐dialkyne.

Sample	*DP*_calc_	*DP*_exp_ ^a^	GME_calc_ [%]	GME_exp_ ^a^ [%]	*M*_n,calc_ [g mol^−1^]	*M*_n,MALDI_ [g mol^−1^]	*M*_n,SEC_ [g mol^−1^]	*Ð*_SEC_
mPEG_79_‐N_3_ ^b^	—	77	0	0	—	3600	3300	1.03
${\mathrm{rPEG}}_{60}^{0.25}$‐N_3_	63	60	25	25	3500	3500^c^	2700	1.10
${\mathrm{rPEG}}_{58}^{0.49}$‐N_3_	54	58	50	49	3600	3900	3200	1.04
${\mathrm{rPEG}}_{42}^{0.75}$‐N_3_	45	32	75	75	3500	3300	2600	1.06
PGME_34_‐N_3_	40	34	100	98^d^	3500	3000	2300	1.04
PPheOzi_16_‐dialkyne	15	16	—	—	2500	2500	1900	1.06

^a^ Determined via ^1^H NMR and MALDI‐TOF MS of rPEG‐OH/PGME‐OH after polymerization;

^b^ Commercially acquired from Biopharma PEG Scientific Inc;

^c^ *M*_p,MALDI_;

^d^ One EO unit is introduced via the initiator.

Throughout this manuscript, the composition of the rPEG polymers is described as ${\mathrm{rPEG}}_{DP}^{f}$, where *f* represents the molar fraction of GME in the sample and *DP* the total degree of polymerization. A detailed description of the *DP*‐ and *f* calculations is given in the Supporting Information (Equations S1 and S2).

As the second building block, a dialkyne functionalized POzi was required for the CuAAC. To ensure systematic comparability among the triblock copolymers with rPEGs of varying GME content, an identical POzi batch was employed for the hydrophobic core. The respective poly(2‐phenyl‐2‐oxazine) (PPheOzi) batch was obtained via cationic ring‐opening polymerization (CROP). The α‐,ω‐dialkyne end groups were introduced using propargyl tosylate as the initiator and potassium pentynoate as the terminating agent, respectively, as previously described [47].

MALDI‐TOF MS revealed a molar mass of *M*_n,MALDI_ = 2500 g mol^−1^ (*DP* = 16) and one distinct distribution, which demonstrates good agreement with calculated *m*/*z* values for the α‐,ω‐dialkyne structure (Figure 2B; Figure S34). The monomodal and narrow molar mass distribution featuring a dispersity of *Ð*  =  1.06 was confirmed by SEC (Figure 2B).

In polymer coupling reactions, quantitative conversion and high product purity are often challenging [48]. The previously described CuAAC, in combination with salting‐out purification, enables the separation of the synthesized ABA triblock copolymer from the starting materials, allowing for the efficient synthesis and purification of ABA triblock copolymers [47]. To ensure high conversion, an excess of the hydrophilic A block is used for the polymer coupling. The residual hydrophilic A polymer is then removed via salting‐out, which can be followed by SEC. This procedure is based on the different solubilities of the hydrophilic A polymer and the amphiphilic ABA triblock copolymer in an aqueous salt solution. The triblock copolymer precipitates at a lower salt concentration and can therefore be isolated from the hydrophilic A polymer. The hydrophilicity of the rPEGs depends on the EO/GME comonomer ratio. It decreases to some extent with increasing GME content, as evident from the lower critical solution temperature (LCST) of 58°C of the PGME homopolymer [28]. To account for this, the applicability of the salting‐out procedure was evaluated using the least hydrophilic A block polymer, PGME_34_‐N_3_, and its respective ABA copolymer. Overlayed SEC traces indicate that the residual PGME A block in the crude product was removed during the purification (Figure 2C). This confirms the successful transfer of the salting‐out procedure to PGME, which allowed the synthesis of PGME_34_‐*b*‐PPheOzi_16_‐*b*‐PGME_34_ (PGME_34_‐**T**). Consequently, the CuAAC, in combination with the salting‐out procedure, was successfully applied to all triblocks (Figure 2C). In the SEC trace of the purified mPEG triblock copolymer, a minor second mode was observable. Its origin could not be conclusively assigned, as it may arise from residual hydrophilic starting material or an AB diblock copolymer impurity. However, given its low intensity, it is unlikely to significantly affect the ensuing formulation studies. To ensure better readability, throughout this manuscript, the ABA triblock copolymers are abbreviated by the respective A block with an additional **T** for triblock, as all samples feature the same B block. For example, ${\mathrm{rPEG}}_{58}^{0.49}$‐*b*‐PPheOzi_16_‐*b*‐${\mathrm{rPEG}}_{58}^{0.49}$ will be referred to as ${\mathrm{rPEG}}_{58}^{0.49}$‐**T**.

In summary, well‐defined rPEG‐azides, PGME‐azide, and PPheOzi‐dialkyne building blocks were successfully synthesized using AROP, followed by post‐polymerization modifications, and CROP, respectively. The ABA triblock copolymers consisting of the hydrophilic mPEG/rPEG/PGME A blocks and the hydrophobic PPheOzi B block were obtained in good purity via the CuAAC reaction.

### 2.2 Drug Solubilization and Micelle Size Distribution

The five polyether‐based carrier polymers were evaluated for their solubilization properties of Efavirenz (EFV), a poorly water‐soluble HIV drug (0.004 g L^−1^) (Figure S40) [49, 50]. To the best of our knowledge, this work presents the first example of combining polyethers with poly(2‐oxazine)s for micellar drug delivery applications. Moreover, PPheOzi is employed here for the first time to facilitate the solubilization of EFV.

Drug formulations were obtained using the thin‐film method. Briefly, the polymer solutions were mixed with drug solutions in varying ratios, using polymer‐drug mass ratios of 10–2 and 10–5, followed by solvent removal to form the thin polymer‐drug film, and subsequent redissolution in an aqueous solution.

**FIGURE 3:**
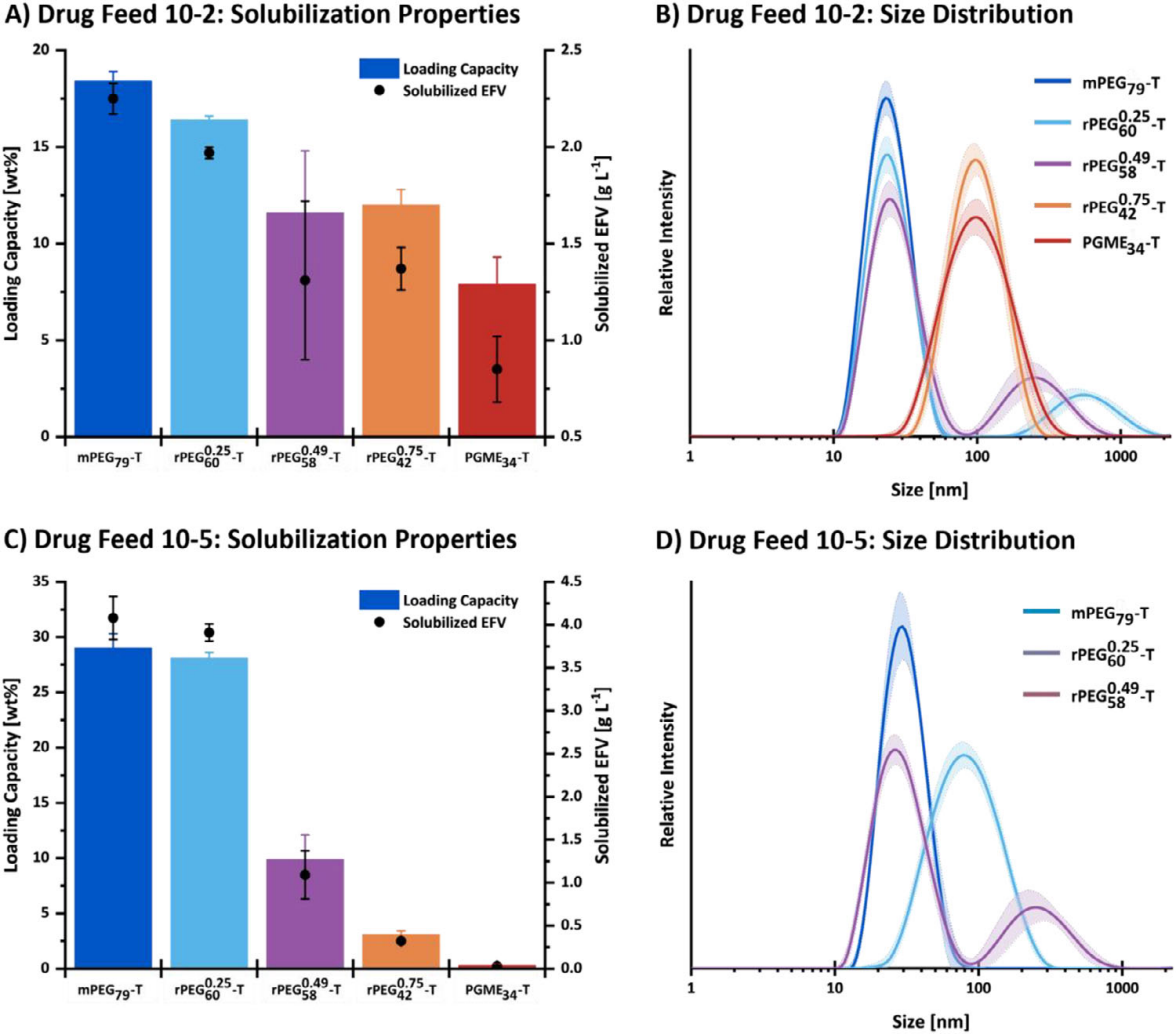
(A, B) Drug‐solubilization and micelle size assessment for a 10–2 polymer‐drug mass ratio. Loading capacity (LC), solubilized Efavirenz, and intensity size distribution, respectively. (C, D) Drug‐solubilization and micelle size assessment for a 10–5 polymer‐drug mass ratio. Loading capacity (LC), solubilized Efavirenz, and intensity size distribution, respectively. LC and solubilized EFV were quantified using HPLC analysis with external calibration. The intensity size distribution was determined using Zetasizer DLS measurements and is visualized as the mean of up to 10 measurements with the standard deviation error.

First, mPEG_79_‐**T** was investigated as a comparison to Poloxamer‐based formulations and as a reference for the rPEG‐**T**‐based formulations. At a drug feed of 2 g L^−1^, quantitative solubilization could be achieved. Increasing the drug feed to 5 g L^−1^ resulted in minor drug precipitation but allowed for the solubilization of 4.1 g L^−1^ EFV. This corresponds to an approximately 1000‐fold increase in EFV solubility and a drug loading capacity (LC) of around 29 wt.% (Figure 3C). Notably, the mPEG_79_‐**T** surpasses the highest reported LC of < 19 wt.% for Poloxamer‐based EFV nanoformulations (20–23 g L^−1^ EFV at 100 g L^−1^ Poloxamer 407) [50, 51]. In addition the ${\mathrm{rPEG}}_{60}^{0.25}$‐**T**‐based formulation, with 25 mol% GME, showed also remarkable solubilization properties, with an LC of 28 wt% (3.9 g L^−1^ EFV) (Figure 3C). This highlights the equal performance ${\mathrm{rPEG}}_{60}^{0.25}$‐**T** to the mPEG_79_‐**T** based formulation. Guided by these results and previous studies, we hypothesize that the high drug loading is attributable to additional hydrogen‐bonding and possibly *π*‐*π* interactions facilitated by PPheOzi [40, 41]. Continuing along with the rPEG series with increasing GME content results in a decrease in effective EFV solubilization. Going from 25 mol% to 49 mol% GME at the same polymer‐drug ratio of 10–5, the loading efficiency (LE) drops from 78 wt.% to 22 wt.%, which indicates an insufficient solubilization at a 10–5 polymer‐drug ratio (Table S1). Furthermore, at 75 mol% and 100 mol% GME, the amount of solubilized EFV drops significantly.

A similar trend was observed for a 10–2 polymer‐drug mass ratio. An increase in GME content, and therefore a slight decrease in the hydrophilicity of the A block, reduced the amount of solubilized EFV (Figure 3A), similar to previous results showing decreased drug solubilization when exchanging the hydrophilic block from poly(2‐methy‐2‐oxazoline) to the less hydrophilic poly(2‐ethyl‐2‐oxazoline) 44. This results in a slightly lower EFV solubilization performance of ${\mathrm{rPEG}}_{58}^{0.49}$‐**T** (LC = 12 wt%) compared to the Poloxamer‐based system (LC < 19 wt%) [50, 51]. Nevertheless, the EFV solubilization was still increased by 300 times compared to the aqueous solubility of EFV (1.31 g L^−1^ compared to 0.004 g L^−1^). Additionally, the reduced antigenicity of ${\mathrm{rPEG}}_{58}^{0.49}$‐**T**, as outlined in the subsequent section, represents a significant advantage that needs to be considered. It should be noted that for the POx and POzi‐based amphiphilic block copolymers, an increase in hydrophobicity of the hydrophobic block is often detrimental to their solubilization capacity, even though, in some cases, aromatic side chains have been found somewhat beneficial [41, 52, 53].

Focusing next on the micelle size distribution, DLS analysis at the lower drug feed (10–2) revealed a pronounced effect of GME content on micelle size (Figure 3B). Formulations containing outer blocks with 0–49 mol% GME consistently self‐assembled into micelles with a *Z*‐average of around 23 nm and a PDI < 0.140. In contrast, formulations with 75–100 mol% GME produced substantially larger micelles (∼90 nm), with higher PDIs of 0.162 and 0.268, respectively. Notably, some samples show the presence of larger aggregates. However, this is a common phenomenon for PMs, and the number of these secondary aggregates is negligibly low considering the correlation of the scattering intensity with *r*_h_^6^ [38]. Interestingly, at the higher drug feed (10–5), the 25 mol% GME formulation exhibited a pronounced shift in size distribution. DLS analysis revealed a monomodal population with a *Z*‐average of 71 nm, with a corresponding PDI of 0.272 (Figure 3D). Building on these observations, the pronounced shift in micelle size at higher drug feed levels suggests potential structural rearrangements within the self‐assembly. Taken together, the data indicate that either swelling of the micellar core, a morphological transition, or fusion into higher‐order structures may have occurred. A possible hypothesis is that the drug loading approached the maximum capacity, resulting in a corona saturated with drug molecules and inducing the highest possible degree of swelling within the PPheOzi core [33, 54]. However, comprehensive morphological investigations, particularly via electron microscopy, are required to gain deeper insight into the resulting nanostructures.

### 2.3 In Vitro Biocompatibility and Antigenicity Assessment

**FIGURE 4:**
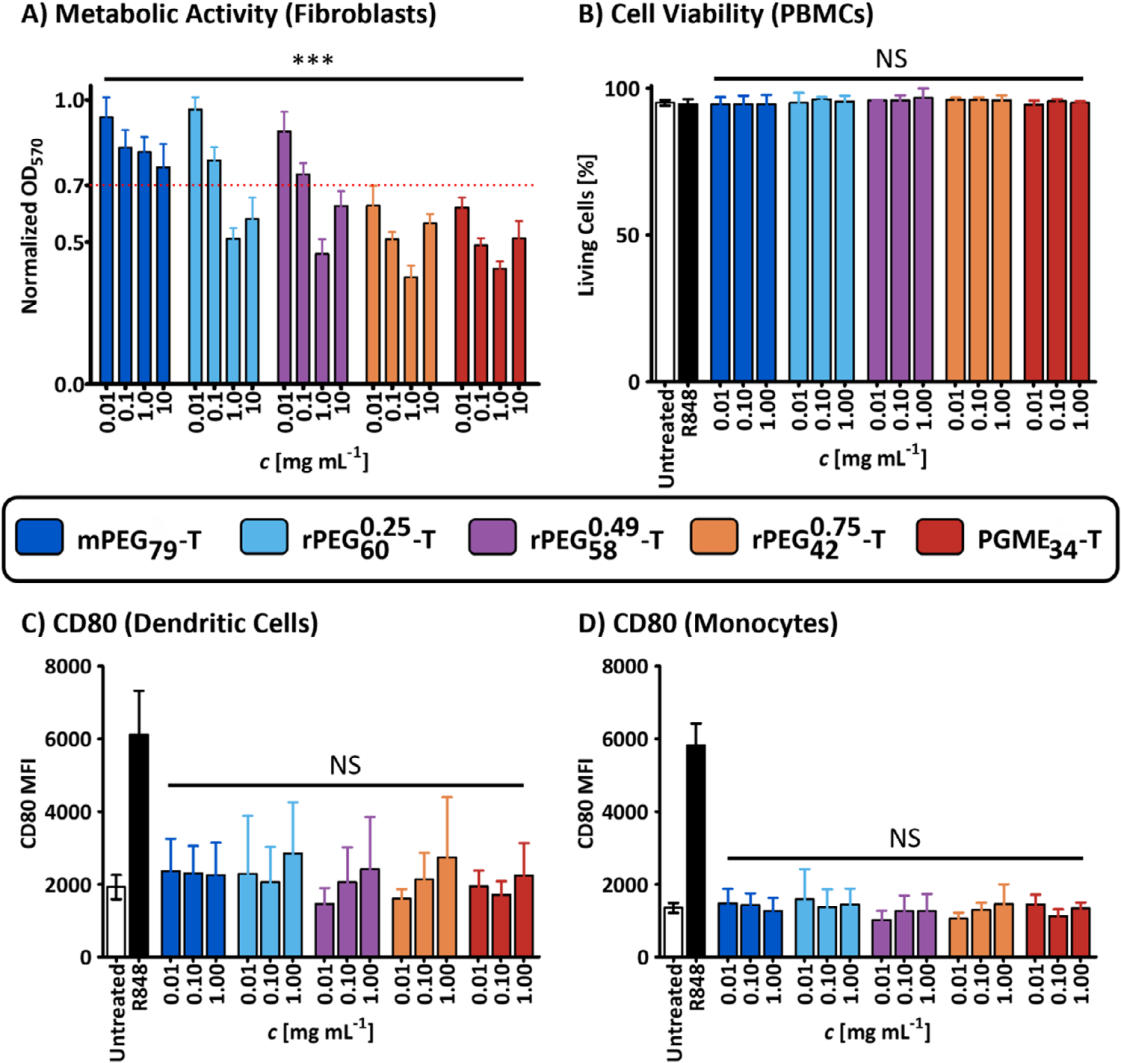
(A) Metabolic activity of murine NIH‐3T3 fibroblasts determined via MTT assay at four concentrations (0.01–10 mg mL^−1^). (B) Cell viability of human leukocytes (PBMCs); (C, D) CD80 expression on dendritic cells (CD19^−^/CD1c^+^) and monocytes (CD19^−^/CD14^+^) as biomarker for immunostimulatory effects, determined via flow cytometry at three concentrations (0.01–1.00 mg mL^−1^). Biological means with standard deviation are presented, *** p < 0.0001, NS (not significant) p > 0.05 as calculated by a two‐way ANOVA between the triblock copolymers and the concentrations.

Furthermore, in vitro biocompatibility and the recognition by anti‐PEG antibodies (APAs) were fundamentally assessed. To this end, the metabolic activity was initially evaluated as an indicator of cytotoxicity using an MTT assay in murine fibroblasts. A concentration‐dependent decrease in metabolic activity was observed across all samples (Figure 4A). The ${\mathrm{rPEG}}_{60}^{0.25}$‐**T** and ${\mathrm{rPEG}}_{58}^{0.49}$‐**T** formulation, with 25 mol% and 49 mol% GME content, featured 90% and >70% normalized metabolic activity at 0.01 and 0.1 mg mL^−1^, respectively. In contrast, the metabolic activity for ${\mathrm{rPEG}}_{42}^{0.75}$‐**T** and PGME_34_‐**T** decreased to ≤ 62% (0.01 mg mL^−1^). However, the highest concentration tested (10 mg mL^−1^) showed increased metabolic activity in comparison to the intermediate concentrations (0.1 and 1.0 mg mL^−1^). This observation is puzzling and cannot be fully explained at this point. A possible hypothesis is that some aggregation or precipitation occurred, resulting in a reduced active concentration to which the cells were exposed during the incubation period. Taken together, the MTT results surprisingly reflect a correlation between an increasing molar GME amount and a decrease in the metabolic activity. Nevertheless, it is neither clearly linear nor clearly threshold‐dominated, and it raises new questions, as previous studies have demonstrated that rPEG has an excellent in vitro biocompatibility [28]. To corroborate the conclusions drawn from the MTT assay, flow cytometry analysis of human peripheral blood mononuclear cells (PBMCs) was employed as a complementary method. Interestingly, in contrast to the MTT results, none of the samples exhibited a reduction in cell viability (Figure 4B). All rPEG‐**T** samples tested within the concentration range of 0.01 to 1.00 mg mL^−1^ maintained approximately 90% viable PBMCs, comparable to the untreated control. Consequently, a reduction in cell viability with increasing GME content is not observed. These seemingly divergent results, when viewed together, highlight distinct yet complementary biological responses: a non‐cytotoxic profile in primary human immune cells across the entire scope, coupled with an inhibitory influence on murine fibroblast proliferation at pharmaceutical less relevant concentrations and high molar GME amounts. However, the fundamental biological response mechanism remains speculative and is intriguing for further research.

Additionally, the flow cytometry analysis revealed that the expression of CD80 on dendritic cells (DC) and monocytes remained at a basal level (Figures 4C, D). These findings provide the first indication that rPEG‐*b*‐PPheOzi‐*b*‐rPEG triblock copolymers do not elicit immunostimulatory responses.

**FIGURE 5:**
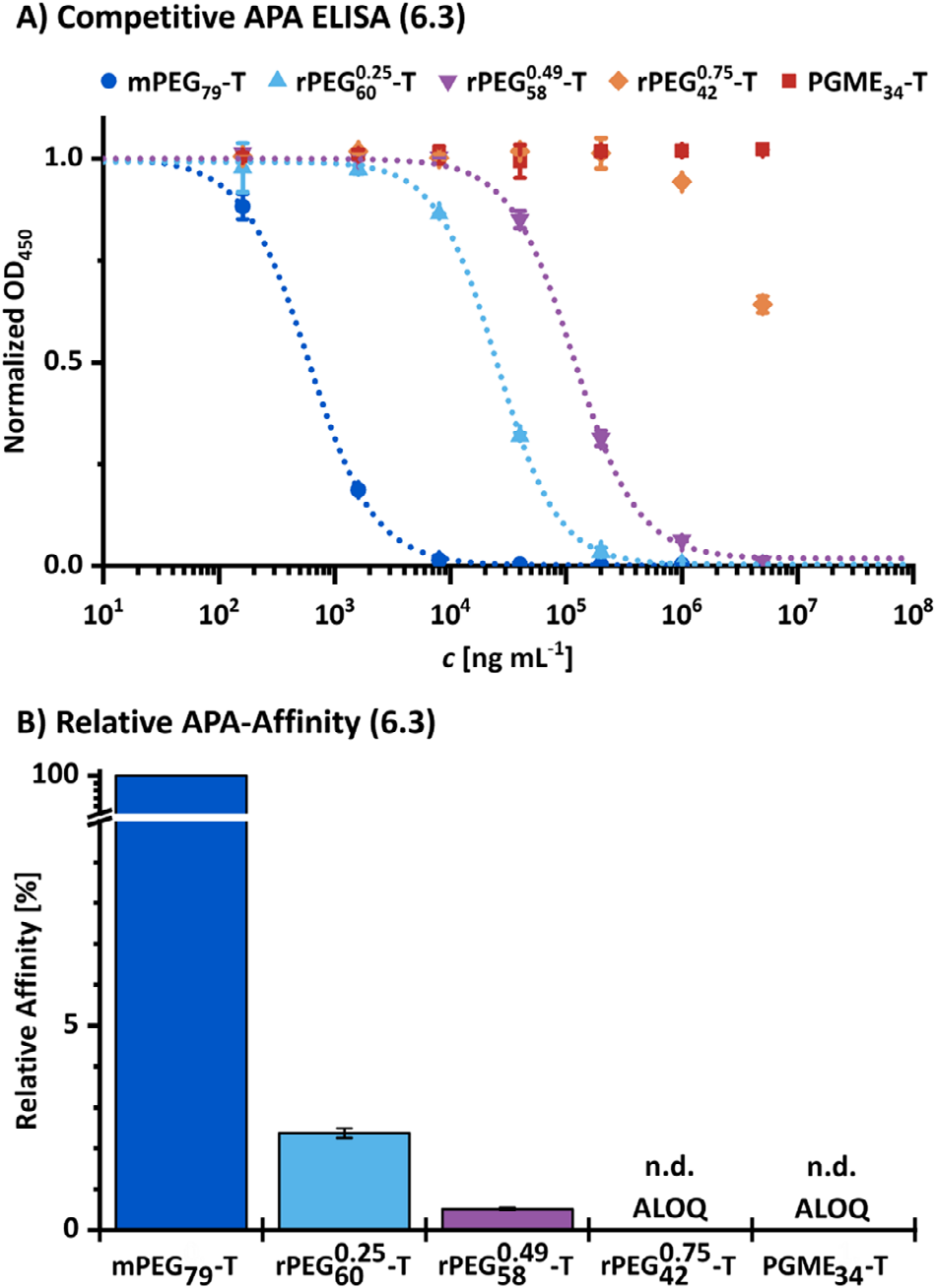
(A) Competitive ELISA performed on an mPEG5k‐NH_2_‐coated Maxisorp well plate using 6.3 as the primary antibody. The triblock copolymer samples were quantitatively evaluated over a concentration range of 160–5 × 10^6^ ng mL^−1^. *EC*_50_ were derived using a four‐parameter logistic (4PL) sigmoidal regression model. For 75 and 100 mol% GME, the EC_50_ values exceeded the upper concentration limit and were therefore reported as being above the limit of quantification (ALOQ). (B) Relative affinity of 6.3 for the investigated rPEG and PGME triblock copolymers normalized to the *EC*_50_ of mPEG_79_‐T. The relative affinities of ${\mathrm{rPEG}}_{42}^{0.75}$‐T, and PGME_34_‐T are effectively close to zero but are reported as n.d. since their *EC*_50_ values were ALOQ.

While immunocompatibility is a prerequisite, the performance of the PEG‐alternative carriers can be nullified due to the recognition by anti‐PEG antibodies (APAs). To study APA binding, the reduced antigenicity of the triblock copolymers was confirmed using competitive enzyme‐linked immunosorbent assay (ELISA) with the backbone‐specific 6.3 APA (murine IgG_1_) employing serial triblock copolymer dilutions. The mPEG_79_‐**T** reference was strongly recognized, with a half‐maximal effective concentration (*EC*_50_) of 600 ng mL^−1^, which was set as 100% relative affinity (Figure 5). Remarkably, a 40‐fold increase in *EC*_50_ was observed with just 25 mol% GME, rising to almost 200‐fold at 49 mol% GME. These shifts translate into a drastic reduction of the relative affinity, dropping to only 2.4% and 0.5%, respectively. For the two remaining formulations (≥75 mol% GME), only a marginal or absent decrease in the optical density (OD) was observed across the concentration range. Because of this, the *EC*_50_ values are considered above the limit of quantification (ALOQ), and no reliable sigmoidal regression could be applied. Accordingly, the relative affinities approach zero. However, they are formally listed as not determined (n.d.) since the *EC*_50_ values are ALOQ. Collectively, these results highlight the inherently low antigenicity of the rPEG‐*b*‐PPheOzi‐*b*‐rPEG system, supporting its suitability for biomedical applications, and the design idea that the randomized PEG (rPEG) technology can be applied as a non‐antigenic drop‐in replacement for PEG.

## 3 Conclusion

The increasing prevalence of anti‐PEG antibodies (APAs) undermines the longstanding benefits of PEGylation. APAs can induce undesired immune responses, e.g., hypersensitivity reactions (CARPA) or accelerated blood clearance (ABC). This raises serious concerns regarding safety and efficiency. The randomized PEG technology (rPEG) tackles this challenge by randomly incorporating glycidyl methyl ether (GME) units while preserving the polyether backbone. Furthermore, rPEG is designed as a “drop‐in” alternative to PEG, for instance, for PEGylated polymeric micelles (PM). PMs are commonly used for the solubilization of poorly water‐soluble drugs. However, clinically established systems feature only moderate drug loadings, which increases the required amount of the potentially immunogenic PEGylated nanocarrier. On the contrary, poly(2‐oxazoline) (POx) and poly(2‐oxazine) (POzi) based PMs permit higher drug loadings.

We addressed the two issues of antigenicity and moderate drug loadings by combining the non‐antigenic rPEG with poly(2‐phenyl‐2‐oxazine) (PPheOzi) into an ABA triblock PM system, utilizing copper‐catalyzed azide‐alkyne cycloaddition (CuAAC) as the polymer coupling reaction. Well‐defined triblock copolymers were prepared, and starting material impurities could be removed during the work‐up procedure. Formulation studies revealed remarkably solubilization properties for the PEG‐ and the 25 mol% GME‐based formulations, with a nearly 1000‐fold increase in Efavirenz (EFV) solubility. In line with previous studies the high drug loading might be attributed to additional polymer‐drug hydrogen‐bonding beside the general hydrophobic interactions. Higher GME contents showed a correlation between increasing GME content and, therefore, the slightly decreasing hydrophilicity of the A block, with a corresponding decrease in the solubilization of EFV. However, it is essential to state that the employed rPEGs/PGME feature a constant molar mass. Consequently, the chain length of the corona decreases with increasing GME content. We strongly assume that maintaining a constant degree of polymerization could yield higher loading capacities, as indicated by the results of a previous study [28]. A systematic investigation of this hypothesis, particularly the effect of the degree of polymerization on the loading capacities, will be the focus of future research.

Employing primary human immune cells showed the absence of cytotoxicity and immunostimulatory effects across all rPEG‐*b*‐PPheOzi‐*b*‐rPEG samples. However, an inhibitory effect on murine fibroblasts was observable at higher concentrations. Therefore, an in‐depth safety analysis is advisable before proceeding with in vivo studies.

Remarkably, the competitive enzyme‐linked immunosorbent assays (ELISA) using anti‐PEG IgG (6.3) revealed that the combination of rPEG, employing only 25 mol% GME with PPheOzi, was already sufficient to effectively suppress antibody recognition to 2.4% relative affinity, while simultaneously achieving excellent drug‐loading capacities. This confirmed the synergistic approach of combining rPEG as a non‐antigenic building block with PPheOzi as a high drug‐loading building block. This work represents an essential step toward establishing the randomized PEG (rPEG) technology. Ongoing investigations will clarify whether the immune system can eventually adapt to such statistics‐based systems. We are confident that the rPEG concept is promising to mitigate undesired recognition and paves the way to safer nanocarrier designs.

## 4 Experimental Procedures

### 4.1 Materials and Instruments

A detailed description of all materials and instruments, including a description of analysis procedures, is referred to in the Supporting Information.

### 4.2 Synthesis of Randomized PEG (rPEG) – Statistical Copolymerization of EO and GME

Randomized PEG (rPEG) was synthesized according to the standard procedure described in a previous publication [28].

Caveat: Ethylene oxide is a highly flammable and toxic gas; it must be handled by trained researchers and staff!

The copolymerization is briefly described for the example of ${\mathrm{rPEG}}_{58}^{0.49}$. This standard procedure was applied to all rPEG samples, varying the ethylene oxide (EO) to glycidyl methyl ether (GME) ratio depending on the targeted molar amount of GME. For PGME only GME was added via a syringe.

Potassium tert‐butoxide (KO*t*Bu, 422 mg, 3.76 mmol, 0.95 eq.) was dissolved in a few milliliters of stabilizer‐free THF containing 1–2 drops of MilliQ water and transferred to an argon‐flushed, flame‐dried flask. A solution of MMEPOH (1‐methoxy‐3‐(2‐methoxyethoxy)propane‐2‐ol, 650 mg, 3.97 mmol, 1.0 eq.) in benzene (5 mL) was added and stirred for 15 min. The solvents were removed under high vacuum at 30°C, and the resulting initiator salt was further dried overnight under high vacuum at 60°C. The dried salt was dissolved in dry DMSO (125 mL) and cooled to −78°C. Glycidyl methyl ether (GME, 9.08 mL, 100.94 mmol, 25.4 eq.) was added via syringe, followed by ethylene oxide (EO, 4.58 mL, 100.94 mmol, 25.4 eq.), which was condensed in a graduated ampule at −78°C and then transferred to the flask. The mixture was allowed to warm to room temperature and stirred at 30°C under static high vacuum for 24 h. After full conversion, DMSO was removed under reduced pressure. The residue was stirred in a mixture of diethyl ether (161 mL), toluene (20 mL), and acetic acid (0.68 mL) for 15 min and then filtered through a dense celite layer. Solvents and excess acetic acid were removed under high vacuum at 60°C to afford ${\mathrm{rPEG}}_{58}^{0.49}$ as a viscous brown liquid (12.96 g, 93%). ^1^H NMR spectra (Figures S1–S4), MALDI‐TOF MS (Figures S20–S23), and SEC traces (Figures S36–S39) of the synthesized rPEGs and PGME are displayed in the Supporting Information.

^1^H NMR (400 MHz, D_2_O *δ*): 3.85‐3.45 (m, polyether backbone), 3.36 (s, OCH_3_) ppm.

### 4.3 Mesylation of Randomized PEG (rPEG‐Ms)

The mesylation procedure was adapted from a previous publication and is exemplified for ${\mathrm{rPEG}}_{58}^{0.49}$‐Ms [32]. The same procedure was applied to all rPEG/PGME‐Ms samples.

${\mathrm{rPEG}}_{58}^{0.49}$ (7.46 g, 2.02 mmol, 1.0 eq.) was dissolved in benzene (70 mL), transferred to an argon‐flushed, flame‐dried Schlenk flask, and dried under high vacuum at 30°C, followed by overnight drying at 60°C. The dried polymer was dissolved in dry DCM (90 mL), and triethylamine (2.24 mL, 16.1 mmol, 8.0 eq.) was added. After 5 min, mesyl chloride (1.25 mL, 16.1 mmol, 8.0 eq.) was added, and the reaction mixture was stirred at room temperature under argon for 3 days. The organic phase was extracted twice with 45 mL aqueous (NH_4_)_2_SO_4_ solution (333 g L^−1^), dried over sodium sulfate, filtered, and concentrated under reduced pressure. The crude product was used in the subsequent azidation step without further purification. ^1^H NMR spectra (Figures S1–S4), MALDI‐TOF MS (Figures S24–S27), and SEC traces (Figures S35–S39) of all synthesized crude rPEG/PGME‐mesyl samples are displayed in the Supporting Information.

^1^H NMR (400 MHz, CD_3_Cl *δ*): 3.75‐3.35 (m, polyether backbone), 3.31 (s, OCH_3_) ppm.

MALDI TOF MS (DCTB, KTFA) *m/z*: calcd for mesyl‐end group: 95.09; found 95.09, average offset < 1.00

### 4.4 Azidation of Randomized PEG‐Ms (rPEG‐N_3_)

The azidation procedure was adapted from a previous publication and is exemplified for ${\mathrm{rPEG}}_{58}^{0.49}$‐N_3_ [32]. The same procedure was applied to all rPEG/PGME‐N_3_ samples.

${\mathrm{rPEG}}_{58}^{0.49}$‐Ms (6.60 g, 1.61 mmol, 1.0 eq.) was dissolved in benzene (30 mL), filtered through a dense celite layer, and rinsed with an additional 20 mL benzene. The filtrate was transferred to an argon‐flushed, flame‐dried Schlenk flask, and the solvent was removed under high vacuum at 30°C, followed by overnight drying at 60°C. The dried polymer was dissolved in dry DMF (65 mL) under argon, and sodium azide (0.84 g, 12.9 mmol, 8.0 eq.) was added under argon flow. The mixture was stirred at 65°C for 3 days. After completion, DMF was removed under reduced pressure, and the residue was dissolved in DCM (100 mL). The organic phase was extracted twice with 50 mL aqueous (NH_4_)_2_SO_4_ solution (333 g L^−1^), dried over sodium sulfate, filtered, and concentrated under reduced pressure. ${\mathrm{rPEG}}_{58}^{0.49}$‐N_3_ was obtained as a viscous brown liquid (5.84 g, 89%). ^1^H NMR spectra (Figures S9–S13), MALDI‐TOF MS (Figures S28–S33), SEC traces (Figure S34–S39), and FT‐IR spectra (Figures S43–S47) of the synthesized rPEG/PGME‐azide and the commercial mPEG_79_‐azide are displayed in the Supporting Information.

^1^H NMR (400 MHz, CD_3_Cl *δ*): 3.75–3.35 (m, polyether backbone), 3.30 (s, OCH_3_) ppm.

MALDI TOF MS (DCTB, KTFA) *m/z*: calcd for azide‐end group: 42.03; found 42.03 with average offset < 1.00

### 4.5 Synthesis of PPheOzi‐dialkyne

PPheOzi was synthesized according to the standard procedure described in a previous publication [47].

Brief description: Distilled propargyl tosylate (1.00 g, 4.79 mmol, 1.0 eq.) was added to an argon‐flushed, flame‐dried Schlenk flask and dissolved in dry benzonitrile (25 mL). Afterward, dry 2‐phenyl‐2‐oxazine (PheOzi, 11.58 g, 71.84 mmol, 15.0 eq.) was added. The mixture was stirred for 24 h at 120°C until full monomer consumption. Potassium pentynoate salt (prepared as previously described) (1.49 g, 10.94 mmol, 2.3 eq.) was added as a termination agent under argon flow [47]. The suspension was stirred at 80°C overnight. Additional acetonitrile was added to lower the viscosity, and the excess of pentynoate salt was removed by centrifugation. The solvents were removed under reduced pressure, and the crude product was precipitated 3 times. First, from chloroform in cold n‐hexane, second, from methanol in water/triethylamine (1% TEA), and third, from methanol in water. PPheOzi_16_ (12.3 g, 4.79 mmol, quant.) was obtained as a brown solid after drying under high vacuum (Figures S14 and S34).

^1^H NMR (500 MHz, CD_2_Cl_2_ *δ*): 7.50‐6.95 (m, phenyl sidechain), 4.18–2.32 (m, ‐NR_2_‐CH_2_‐ and O‐CH_2_‐), 2.10–1.27 (‐CH_2_‐) ppm.

### 4.6 ABA Triblock Copolymer Synthesis via Copper‐Catalyzed Azide‐Alkyne Cycloaddition

The copper‐catalyzed azide‐alkyne cycloaddition (CuAAC) was performed according to a previously reported protocol, using one equivalent of dialkyne‐functionalized PPheOzi_16_ and at least 2.1 equivalents of mPEG‐N_3_, rPEG‐N_3_, or PGME‐N_3_, respectively [47].

As an example, PPheOzi_16_ (0.70 g, 285 µmol, 1.0 eq.) and ${\mathrm{rPEG}}_{58}^{0.49}$‐N_3_ (2.77 g, 711 µmol, 2.5 eq.) were dissolved in acetonitrile (55 mL). PMDTA (*N,N,N',N'',N''*‐pentamethyldiethylenetriamine, 0.12 mL, 569 µmol, 2.0 eq.) was added, and the solution was degassed under argon for 15 min. Copper(I)bromide (81.6 mg, 569 µmol, 2.0 eq.) was added to an argon‐flushed, flame‐dried Schlenk flask, and the degassed polymer solution was transferred to this flask. The reaction mixture was stirred overnight at 50°C. After completion, the solvent was removed under reduced pressure. Excess hydrophilic polymer was removed via salting out: the crude product was dissolved in water (100 g L^−1^), and an aqueous (NH_4_)_2_SO_4_ solution (500 g L^−1^) was added until the triblock copolymer precipitated. To avoid unwanted precipitation of hydrophilic polymers, preliminary solubility tests were conducted to determine the maximum applicable (NH_4_)_2_SO_4_ concentration. The suspension was cooled in an ice bath and allowed to equilibrate overnight at room temperature. The precipitate was collected by centrifugation and dissolved in toluene to remove residual water by azeotropic distillation under reduced pressure.

Finally, the crude product was dissolved in acetonitrile and purified by filtration through a SiO_2_/MgSO_4_ (1:1) plug, rinsing several times with acetonitrile. The solvent was removed under reduced pressure to afford ${\mathrm{rPEG}}_{58}^{0.49}$‐*b*‐PPheOzi_16_‐*b*‐${\mathrm{rPEG}}_{58}^{0.49}$ (1.7 g, 167 µmol, 59%) as a brown waxy solid. The ^1^H NMR spectra of all ABA triblock copolymers are displayed in the Supporting Information (Figures S15–S19).

### 4.7 Drug‐Loaded Polymeric Micelles (Thin‐Film Method)

Drug‐loaded micelles were prepared via the thin‐film method [55]. For each formulation, 150 µL of an ethanolic polymer solution (20 g L^−1^) was mixed with varying volumes of an ethanolic Efavirenz (EFV) solution (20 g L^−1^) (Figure S40). The solvent was evaporated at 50°C under a gentle nitrogen stream to form a thin film, followed by drying under high vacuum for 30 min.

The films were rehydrated with 300 µL pre‐warmed deionized water (37°C), resulting in *c*_polymer‐feed_  =  10 g L^−1^ and *c*_drug‐feed_ = 2 or 5 g L^−1^, respectively. To ensure complete solubilization, mPEG and rPEG‐based triblock copolymers were mixed at 55°C and 1100 rpm for 12 min using an Eppendorf Thermomixer Compact 5355. In contrast, PGME‐based triblocks were mixed at 42°C and 1100 rpm for 30 min due to their cloud point (*T*_cp_ = 47°C, Figure S53). Formulations were centrifuged at 10000 rpm for 5 min (Eppendorf centrifuge 5424) to remove any non‐solubilized drug. The solubilized EFV was quantified via HPLC in triplicate: 10 µL of the supernatant was diluted with 990 µL acetonitrile, filtered through a 0.45 µm syringe filter, and stored at 4°C before analysis. Solubilized EFV (*c*_drug‐solubilized_) was determined using a linear regression (OriginPro 2024) of EFV standards (5–80 mg L^−1^) (Figure S41).

The loading efficiency (LE) and loading capacity (LC) were calculated according to the following Equations 1 and 2, where *c*_drug‐solubilized_ is the solubilized drug concentration, *c*_drug‐feed_ and *c*_polymer‐feed_ are the initial drug and polymer concentrations:

(1) $$LE=\frac{c_{\mathrm{drug}-\mathrm{solubilized}}}{c_{\mathrm{drug}-\mathrm{feed}}}*100\%$$

(2) $$LC=\frac{c_{\mathrm{drug}-\mathrm{solubilized}}}{c_{\mathrm{drug}-\mathrm{solubilized}}+c_{\mathrm{polymer}-\mathrm{feed}}}*100\%$$

## Author Contributions

**Julian Schmidt**: Conceptualization, Data Curation (lead), Investigation (lead), Methodology, Project Administration, Visualization (lead), Writing – Original Draft Preparation (lead), Writing – Review and Editing. **Anna‐Lena Ziegler**: Conceptualization (supporting), Methodology, Writing – Review and Editing. **Florian T. Kaps**: Conceptualization (supporting), Investigation (supporting), Writing – Review and Editing. **Laura Rosenberger**: Conceptualization (supporting), Investigation (supporting), Data Curation (supporting), Visualization (supporting), Writing – Review and Editing. **Matthis Bros**: Conceptualization (supporting), Funding Acquisition, Supervision, Writing – Review and Editing. **Holger Frey**: Conceptualization, Funding Acquisition, Supervision, Writing – Review and Editing. **Robert Luxenhofer**: Conceptualization, Funding Acquisition, Supervision, Writing – Review and Editing.

## Funding

J.S. and H.F. acknowledge funding from the European Research Council, ERC Advanced Grant for the project RandoPEGmed (Project No. 101055434). This work was further supported by the Research Council of Finland (decision numbers: 342983 R.L. and 352397 R.L.). A.‐L.Z. acknowledges financial support through the CHEMS doctoral program at the University of Helsinki. Work of L.R. and M.B. is supported by the Carl Zeiss Stiftung (Nano@Liver, P7).

## Conflicts of Interest

The authors declare no conflicts of interest.

## Supporting information




**Supporting File**: marc70178‐sup‐0001‐SuppMat.docx.

## Data Availability

The data that support the findings of this study are available from the corresponding author upon reasonable request.
