# Supplementary material for: A Non‐Antigenic Randomized Polyethylene Glycol/Poly(2‐Phenyl‐2‐Oxazine)‐Based Drug Delivery Platform
Source: Macromol Rapid Commun. 2025 Dec 24;47(14):e00781. doi: 10.1002/marc.202500781 (PMC13384809; doi:10.1002/marc.202500781)
Supplement: Supplementary file 1 — Supporting File: marc70178‐sup‐0001‐SuppMat.docx. [file MARC-47-e00781-s001.docx]

Supporting Information

A Non-Antigenic Randomized Polyethylene Glycol/Poly(2-Phenyl-2-Oxazine)-Based Drug Delivery Platform

Julian Schmidt, Anna-Lena Ziegler, Florian T. Kaps, Laura J. Rosenberger, Matthias Bros, Holger Frey*, Robert Luxenhofer*

J. Schmidt (orcid: 0009-0000-1206-099X), H. Frey* (orcid: 0000-0002-9916-3103)

Department of Chemistry, Johannes Gutenberg University Mainz, 55128 Mainz, Germany

E-mail: hfrey@uni-mainz.de

A.-L. Ziegler (orcid: 0009-0003-6963-1628), F. T. Kaps (orcid: 0009-0005-8518-0518), R. Luxenhofer* (orcid: 0000-0001-5567-7404)

Soft Matter Chemistry, Department of Chemistry, and Helsinki Institute of Sustainability Science, Faculty of Science, University of Helsinki, Helsinki 00014, Finland

E-mail: robert.luxenhofer@helsinki.fi

L. J. Rosenberger (orcid: 0009-0004-8477-7892), M. Bros

Department of Dermatology, University Medical Center of the Johannes Gutenberg University Mainz, 55101 Mainz, Germany

**1. Supporting Information Text**

**1.1 Supplementary Text**

*rPEG Sample Description*:^[1]^

Throughout this manuscript and the Supporting Information, the randomized PEG (rPEG) samples’ composition is described with:

$$\text{rPEG}_{\text{DP}}^{\text{f}}$$

where *f* is the molar fraction of GME in the sample and *DP* is the total degree of polymerization. All samples carry a methoxy group at the *α*-chain end. After the polymerization, the *ω*-chain end consists of primary and secondary hydroxyl groups, which are modified to mesyl groups and subsequently converted into azide groups.

*Calculation of incorporated GME content and total degree of polymerization*:^[1]^

The calculation of the GME content and total degree of polymerization (*DP*_total_) in the rPEG samples is not feasible via end group analysis from the respective ^1^H NMR spectra because of the overlap of methoxy groups deriving from the initiator and the methoxy methylene side groups. Therefore, the GME content was determined by a combination of MALDI TOF MS and ^1^H NMR spectroscopy. First, the number average molar mass (*M*_n,MALDI_) and the ratio (*I*_backbone_/*I*_MeO_) of the integrals of backbone signals (*I*_backbone_) from 3.90 to 3.45 ppm to methoxy group signals (*I*_MeO_) from 3.45 to 3.25 ppm were determined via MALDI TOF MS and ^1^H NMR spectroscopy, respectively. Based on the following relationships, *DP*_GME_ and, therefore, the molar content of GME (mol%_GME_) and *DP*_total_ of the rPEGs are calculated by choosing values that fit the experimental data.

| $\left( \frac{I_{\mathrm{backbone}}}{I_{\mathrm{MeO}}} \right)=\frac{5\cdot{DP}_{\mathrm{GME}}+4\cdot{DP}_{\mathrm{EO}}}{3\cdot{DP}_{\mathrm{GME}}+3}$ | (S1) |
| --- | --- |
| ${DP}_{\mathrm{total}}={DP}_{\mathrm{GME}}+{DP}_{\mathrm{EO}}=\frac{M_{n,MALDI}-M_{initiator group}-M_{end group}}{44.05\cdot\left( \frac{100-\text{mol\%}_{\mathrm{GME}}}{100} \right)+88.11\cdot\left( \frac{\text{mol\%}_{\mathrm{GME}}}{100} \right)}$ | (S2) |

*ABA Triblock Copolymer Description*:

Throughout this manuscript and the Supporting Information, the ABA triblock copolymers are abbreviated by the respective A block with an additional **T** for triblock, as all samples feature the same B block. For example, $\text{rPEG}_{\text{58}}^{\text{0.49}}$-*b*-PPheOzi_16_-*b*-$\text{rPEG}_{\text{58}}^{\text{0.49}}$ will be referred to as $\text{rPEG}_{\text{58}}^{\text{0.49}}$-**T**.

*Model drug: Efavirenz*

Efavirenz (EFV, IUPAC: (4*S*)-6-Chlor-4-(2-cyclopropylethinyl)-4-(trifluormethyl)-2*H*-3,1-benzoxazin-2-on, **Figure S40**) is used as an antiretroviral combination therapy for HIV-1 infections. EFV is a non-nucleoside reverse transcriptase inhibitor and is commonly applied in combination with two nucleoside analogue reverse transcriptase inhibitors. EFV features a long plasma half-life, which enables a single dose per day. However, its poor aqueous solubility (<10 µg mL^-1^) poses a challenge for further improvement.^[2]^

**1.2 Materials and Instruments**

*Reagents and Equipment*:

All chemicals and solvents were purchased from Acros Organics, Roth, TCI Chemicals, Sigma Aldrich, Fisher Scientific, BLDpharm, Avantor VWR, Deuero GmbH, and abcr GmbH, unless otherwise noted. Ethylene Oxide was acquired from Air Liquide. For the anionic ring-opening polymerization (AROP), THF was passed through basic aluminum oxide, and glycidyl methyl ether was dried over CaH₂ before cryo-transfer for polymerization. For the cationic ring-opening polymerization (CROP), phenyl oxazine and benzonitrile were dried over CaH₂ and P₂O₅, respectively, and subsequently distilled prior to use. The CROP initiator, propargyl *p*-toluenesulfonate (propargyl tosylate), was also distilled before use.

*NMR spectroscopy*:

^1^H spectra were recorded either on a Bruker Avance III HD 400 spectrometer with 400 MHz, respectively, or on a Bruker Avance III 500 spectrometer with 500 MHz, or on a Bruker Avance III 600 spectrometer with 600 MHz and referenced internally to residual proton signals of the deuterated solvent. All spectra were acquired at room temperature. Spectra were processed and analyzed utilizing the MestReNova 14.3.3-33362 software.

*FT-IR spectroscopy*:

FT-IR spectroscopy was conducted using a *Nicoleti* iS10 FT-IR spectrometer (Thermo Scientific, Waltham, MA, USA) with a diamond ATR unit.

*Size exclusion chromatography (SEC)*

SEC analysis was primarily conducted on an Agilent 1100 series HPLC system equipped with a degasser, isocratic pump (G1310A), autosampler (G1313A), column oven (G1316A), and refractive index (RI, G1310A) and variable wavelength (VWD, G1314A) detectors. Separations were performed using a four-column setup (MZ-Analysentechnik GmbH): (i) HEMA-40 guard (40 Å, 10 µm, 50 × 8.0 mm), (ii) HEMA-40 analytical (40 Å, 10 µm, 300 × 8.0 mm), (iii) HEMA-100 analytical (100 Å, 10 µm, 300 × 8.0 mm), and (iv) HEMA-300 analytical (300 Å, 10 µm, 300 × 8.0 mm). The eluent was DMF containing 1 mg mL^-1^ anhydrous LiBr at 1.0 mL min^-1^. The column oven and RI detector were maintained at 50 °C. Calibration employed PEG standards (PSS Polymer Standards Service GmbH) with *M*_p_ values ranging from 106 to 42700 g mol^-1^. Samples (1 mg mL^-1^ in DMF/LiBr with one drop toluene) were filtered through 0.45 µm PTFE filters, and 100 µL were injected; toluene served as an internal standard. Data was analyzed with PSS WinGPC Unichrom V8.31.

For reaction and purification monitoring of ABA triblock copolymers, an Agilent 1260 Infinity II SEC-MDS system was used. Separations employed Waters Styragel columns (HT5, HR4, HR2; 300 mm) at 40 °C with DMF/LiBr (1 g L^-1^) as eluent at 0.8 mL min^-1^. Calibration was based on PMMA standards (PSS, *M*_p_ = 602 g mol^-1^ to 1.51 x 10^6^ g mol^-1^). Samples were dissolved in DMF/LiBr (1 mg mL^-1^) and filtered through 0.45 µm PTFE filters before injection.

*MALDI-TOF Mass Spectrometry*:

MALDI-TOF MS measurements of the rPEG azides were carried out using a Bruker autoflex maX MALDI-TOF using a smartbeam-II solid state laser with a wavelength of 337 nm. Spectra were recorded using Bruker flexControl 3.4 software and analyzed using Bruker flexAnalysis 3.4 and Bruker polytools 1.31. Potassium trifluoroacetate (KTFA) and trans-2-[3-(4-tert-butylphenyl)-2-methyl-2-propenylidene]malononitrile (DCTB) were utilized as ionization salt and matrix, respectively. For sample preparation, the polymers were dissolved in chloroform at 10 mg mL^-1^. 20 µL of the sample solution was combined with 20 µL of the DCTB-matrix solution (10 mg mL^-1^ in chloroform). 5 µL of a 0.1 m KTFA solution in methanol was added, and 1 µL of the resulting mixture was spotted onto an MTP 384 ground steel target plate. The solvents were allowed to evaporate completely before the measurements. All measurements were performed in linear mode.

MALDI-TOF MS measurements of PPheOzi_16_ were performed using a Shimadzu AXIMA Performance MALDI-TOF mass spectrometer. Sodium trifluoroacetate (NaTFA) and trans-2-[3-(4-tert-butylphenyl)-2-methyl-2-propenylidene]malononitrile (DCTB) were utilized as ionization salt and matrix, respectively. The samples were prepared as follows: 10 µL of polymer solution (10 g L^-1^ in methanol) and 10 µL NaTFA solution (5 g L^-1^ in methanol) were mixed with 10 µL of DCTB-matrix solution (100 g L^-1^ in acetonitrile). 1 µL of the mixture was spotted onto a stainless-steel target plate. Measurements were carried out in reflector mode. Calibration was performed using PEG (2000 g mol^-1^) standards.

*Turbidimetry*:

Turbidimetric measurements were performed with a *JASCO* UV-Vis Spectrometer (V750) at a wavelength of 600 nm and a heating rate of 1 K min^-1^ using the software *JASCO Spectra Manager.* Polymers were dissolved in deionized water at 10 mg mL^-1^. Pure deionized water was utilized as a reference value of 100% transmittance and was measured prior to each experiment. All measurements were performed in a quartz glass cuvette with a light path of 10 mm. Cloud point temperatures (*T*_cp_) were determined at a transmittance of 50%.

*HPLC*:

High-performance liquid chromatography (HPLC) was performed using a Shimadzu Prominence system (Tokyo, Japan) equipped with an FCV-11AL valve unit, DGU-20A degasser, LC-20AD UPLC pump, SIL-20A HT autosampler, CTO-20AC column oven, CBM-20A communication module, and SPD-M20A diode array detector (DAD). Chromatographic separation was carried out on a Zorbax Eclipse Plus C18 column (4.6 × 100 mm, 3.5 µm) at 25 °C using an isocratic mobile phase composed of acetonitrile and water (50:50, v/v) containing 0.05% trifluoroacetic acid (TFA). The flow rate was set to 1.0 mL min^-1^, the injection volume was 50 µL, and the total run time was 15 minutes. Detection was performed at 248 nm using a UV/VIS spectrophotometer. The loading efficiency (LE) and loading capacity (LC) were calculated according to the following **Equation S3 and S4**, where *c*_drug-solubilized_ is the solubilized drug concentration, *c*_drug-feed_ and *c*_polymer-feed_ are the initial drug and polymer concentrations:

| $LE= \frac{c_{\text{drug-solubilized}}}{c_{\text{drug-feed}}}*100\%$ | (S3) |
| --- | --- |
| $LC= \frac{c_{\text{drug-solubilized}}}{c_{\text{drug-solubilized}}+ c_{\text{polymer-feed}}}*100\%$ | (S4) |

*Zetasizer*:

DLS measurements were conducted using a Malvern Instruments Zetasizer Nano-ZS equipped with a 4 mW He−Ne laser (*λ* = 633 nm). Hydrodynamic radii were determined at a scattering angle of 173° and a temperature of 37 °C. Samples were equilibrated at 37 °C for 30 minutes before analysis. Each sample was measured multiple times, with 10 – 16 scans per measurement and a 5-minute gap. The micelles were prepared using the thin-film method before measurement. Insoluble (if any) drug was removed by centrifugation. The solutions were diluted with 2 mm aqueous NaNO_3_ solution to a final polymer concentration of 2 g L^-1^ and filtered through a 0.45 µm syringe filter into the cuvette.

*CMC determination using coumarin-6 method*:^[3]^

The critical micelle concentration (CMC) was determined using a slightly modified version of the coumarin-6 fluorescent probe protocol reported by A. Fluksman and O. Benny.^[3]^

For each sample 37.5 µL of a 6 µM coumarin-6/DCM solution added to a vial. The organic solution was completely removed; first by 30 minutes evaporation in the fume hood, followed by 30 minutes evaporation under vacuum. Afterwards, 300 µL of triblock copolymer solutions in PBS (1.0 – 0.0005 g L^-1^) were transferred to the coumarin-6. Solubilization of the fluorescent probe was promoted by 60 min shaking (1150 rpm) at 37 °C, followed by at least 14 hours shaking (600 rpm) at room temperature and again 60 minutes shaking (1150 rpm) at 37 °C. The last shaking step was performed imminently before the measurement. For the measurement 200 µL of test solution were transferred to a black 96 well plate and the fluorecene intensity was measured using a FLUOstar Omega multimode reader (BMG Labtech) with automatic gain adjustment and an 485-12/EM520 optical filter set. The CMC was determined as the intersection of two linear regressions as discribed by A. Fluksman and O. Benny.

*MTT Assay*:

Murine NIH-3T3 fibroblasts (CRL-1658, ATCC) were cultured under standard conditions at 37 °C and 5% CO_2_ in Dulbecco’s Modified Eagle Medium (DMEM; high glucose, GlutaMAX™ Supplement, pyruvate; Thermo Fisher Scientific) supplemented with 10% iron-supplemented calf serum (HyClone™) and 1% penicillin-streptomycin (100 IU mL^-1^ penicillin and 100 µg mL^-1^ streptomycin). Cells were passaged 2 – 3 times per week at a subculture ratio of 1:10 to 1:15 using TrypLE™ Express Enzyme.

For the MTT assay, cells were seeded at a density of 5 – 10 x 10^3^ cells per well on a 96-well plate (SARSTEDT AG & Co. KG). The cells were incubated overnight at standard conditions. Subsequently, the culture medium was removed, and the cells were treated with 100 µL ABA triblock copolymer solution (6 replicates, final concentrations 10, 1.0, 0.1, 0.01 mg mL^-1^ in complete DMEM medium) and controls. Following 24 hours of incubation at standard conditions, the medium was replaced with 100 µL of 0.5 mg mL^-1^ MTT (Thiazolyl Blue Tetrazolium Bromide, Merck KGaA) solution in DMEM. After 4 hours of incubation at standard conditions, formazan crystals were solubilized by adding 200 µL dimethyl sulfoxide per well. Absorbance was measured using a BioTek Synergy H1 plate reader at 570 nm (OD₅₇₀), with a reference reading at 630 nm (OD₆₃₀) subtracted to correct for background. Relative metabolic activity was calculated by normalizing the corrected OD values to untreated control cells cultured in complete DMEM, which were set as 100%. The mean of three independent biological replicates was calculated with error propagation.

*Flow Cytometry:*

Peripheral blood mononuclear cells (PBMCs) were isolated from Buffy coats of blood donations of healthy donors by the Blood Transfusion Unit of the University Medical Center Mainz. Isolation was performed under sterile conditions using a laminar flow bench. For PBMC isolation, 20 mL Histopaque-1077 (1.077 g mL^-1^, Sigma-Aldrich) was carefully over-layered with 20 mL of blood. Tubes were centrifuged at 700 g for 20 min at room temperature with break switched off. The plasma phase was then heat-inactivated for at least 30 min at 56 °C, centrifuged (5 min, 4 °C, 2200 g), and stored at 4 °C until further usage. The PBMCs were retrieved from the interphase after density gradient centrifugation and washed with 30 mL PBS. The mixture was centrifuged (10 min, 4 °C, 600 g), and the supernatant was discarded. After this, the washing procedure was repeated five times with 50 mL PBS. PBMC were cultured in RPMI-1640 media (Thermo Fisher Scientific), supplemented with 2% of heat-inactivated autologous plasma, 2 mm l-glutamine, 100 U mL^-1^ penicillin, 100 µg mL^-1^ streptomycin, 50 µm 2-mercaptoethanol (all from Sigma-Aldrich). PBMCs were seeded into FACS tubes (2.5 x 10^6^ cells in 0.5 mL), and the triblock copolymer samples were added, resulting in final concentrations of 0.01 to 1 mg mL^-1^. Additionally, the positive control was treated with resiquimod (R848, 1 µg mL^-1^) to assess immunostimulatory effects. Samples were incubated overnight at 37 °C and 5% CO_2_ in a humidified atmosphere as indicated. On the next day, 2 mL PBS was added to all tubes and centrifuged at 300 g and 4 °C for 10 min. Samples were then incubated with 20 µL FcR blocking reagent (Miltenyi Biotec), 1:100 in FACS buffer (PBS, 2 mm EDTA, 0.5% fetal calf serum) for 10 min at room temperature. Cells were then incubated with fluorescence-label antibodies against lineage and immune activation markers specific for CD80 (PerCP-eFluor710, clone 2D10.4), CD19 (SuperBright 702, clone HIB19), CD1c (BV605, clone L161), and CD14 (PE-eFluor610, clone 61D3) for 20 min at 4 °C. All antibodies were purchased from Thermo Fisher Scientific. Subsequently, cells were washed with 1 mL FACS buffer and incubated with 100 µL LIVE/DEAD™ Fixable Scarlet (723) Viability Kit (1:5000 in PBS, Thermo Fisher Scientific) for 30 min at 4 °C to identify dead cells. Samples were stored at 4°C until subjection to flow cytometric analysis using an Attune NxT flow cytometer (Lasers: BRVK, Instrument model: 4486521, Thermo Fisher Scientific). Data was analyzed using Attune NxT software v3.1.1 according to the gating strategy depicted in **Figure S** *42*. Mean values and standard deviation were visualized using GraphPad Prism 5.

*Competitive anti-PEG antibody ELISA:*

We established a customized ELISA protocol for rPEG samples adapted from the protocols reported by Roffler et al.^[4]^ In detail, Maxisorp 96-well plates (Thermo Fisher Scientific) were coated with 0.5 µg per well mPEG_114_-amine (Biopharma PEG) in NaHCO_3_/Na_2_CO_3_ buffer (0.1 m, pH 9.5) overnight at 4 °C. The coating solution was discarded, and the plate was washed three times with 300 µL PBS per well. Blocking was performed using 5% (*w/v*) skim milk powder in PBS for 2 hours at 21 °C, followed by one washing step with 300 µL PBS. 50 µL of the competitive triblock copolymer samples (320 – 1 x 10^7^ ng mL^-1^ in PBS) were added in duplicate, as well as 50 µL of PBS as a negative control (12 wells), and incubated for 30 min at 21 °C with shaking at 300 rpm. 6.3 (IBMS Academia Sinica, Taiwan) was diluted with 4% (*w/v*) skim milk powder in PBS at a final concentration of 50 ng mL^-1^. 50 µL of the primary antibody mixture was added to the plate and incubated (1 h, 21 °C, 300 rpm). Note: The analyzed concentrations of the competitive samples and the primary antibody are reduced by a dilution factor of 2. Unbound antibodies, antibodies bound to the competitive analyte, as well as unbound analytes, were removed by washing with 2 x 300 µL 0.1% (*w/v*) CHAPS/PBS (3-((3-Cholamidopropyl)dimethylammonio)-1-propanesulfonate) and 1 x PBS. Detection was performed using 50 µL horseradish peroxidase conjugated AffiniPure donkey anti-mouse IgG (H+L) (1:10000, Immuno Jackson Research) for 1 hour at 21 °C with 300 rpm shaking, followed by washing with 4 x 300 µL 0.1% (*w/v*) CHAPS/PBS and 2 x 300 µL PBS. Subsequently, 100 µL of 1-Step TMB ELISA Substrate Solution (Thermo Fisher Scientific) was incubated for 20 minutes at 21 °C with shaking at 300 rpm. The reaction was stopped with the addition of 100 µL H_2_SO_4_ (2 m). Absorbance was measured using a FLUOstar Omega multi-mode reader (BMG Labtech) at 450 nm (OD_450_), with reference reading at 570 nm (OD_570_) subtracted for background correction. The corrected data were normalized to the mean of 12 PBS controls. Half-maximal effective concentrations (*EC*_50_) were derived using a four-parameter logistic (4PL) sigmoidal regression model using Origin2024 Pro. Subsequently, the relative affinities of the samples were determined by normalization to the reference sample (mPEG_79_-**T**) with error propagation.

**2. Supporting Tables**

Table S 1: Drug-loading characteristics including solubilized EFV concentration, loading efficiency (LE), and loading capacity (LC) determined via HPLC, as well as size-distribution parameters (Z-average and PDI) calculated using weighted variance analysis.

| Triblock  copolymer | Polymer-Drug  Mass Ratio | HPLC Analysis | | | DLS Analysis | |
| --- | --- | --- | --- | --- | --- | --- |
|  |  | *c*_EFV-solubilized_ | LE [wt%] | LC [wt%] | *Z*_av_ [nm] | PDI |
| mPEG_79_-**T** | 10-2 | 2.25 ± 0.08 | 113 ± 4 | 18.4 ± 0.5 | 22.8 ± 0.3 | 0.105 ± 0.010 |
| $\text{rPEG}_{\text{60}}^{\text{0.25}}$-**T** | 10-2 | 1.97 ± 0.03 | 98 ± 2 | 16.4 ± 0.2 | 23.2 ± 0.3  530.1 ± 94.7 | 0.109 ± 0.014  0.203 ± 0.027 |
| $\text{rPEG}_{\text{58}}^{\text{0.49}}$-**T** | 10-2 | 1.31 ± 0.41 | 65 ± 20 | 11.6 ± 3.2 | 24.7 ± 0.5  246.6 ± 36.5 | 0.139 ± 0.028  0.197 ± 0.032 |
| $\text{rPEG}_{\text{42}}^{\text{0.75}}$-**T** | 10-2 | 1.36 ± 0.11 | 68 ± 5 | 12.0 ± 0.8 | 90.8 ± 0.8 | 0.162 ± 0.020 |
| PGME_34_-**T** | 10-2 | 0.85 ± 0.17 | 43 ± 8 | 7.9 ± 1.4 | 88.5 ± 2.3 | 0.268 ± 0.045 |
| mPEG_79_-**T** | 10-5 | 4.08 ± 0.25 | 82 ± 5 | 29.0 ± 1.3 | 28.7 ± 0.7 | 0.105 ± 0.023 |
| $\text{rPEG}_{\text{60}}^{\text{0.25}}$-**T** | 10-5 | 3.91 ± 0.10 | 78 ± 2 | 28.1 ± 0.5 | 70.6 ± 0.6 | 0.272 ± 0.027 |
| $\text{rPEG}_{\text{58}}^{\text{0.49}}$-**T** | 10-5 | 1.09 ± 0.28 | 22 ± 6 | 9.9 ± 2.2 | 26.2 ± 0.6  248.7 ± 36.3 | 0.168 ± 0.022  0.191 ± 0.020 |
| $\text{rPEG}_{\text{42}}^{\text{0.75}}$-**T** | 10-5 | 0.32 ± 0.03 | 6 ± 1 | 3.1 ± 0.3 | n.d.^a)^ | n.d.^a)^ |
| PGME_34_-**T** | 10-5 | 0.03 ± 0.01 | 1 ± 0 | 0.3 ± 0.1 | n.d.^a)^ | n.d.^a)^ |

^a)^Not determined.

Table S 2: Results of the competitive ELISA performed on a mPEG^114^-NH_2_-coated Maxisorp 96-well plate using 6.3 as the primary antibody. Half-maximal effective concentrations (EC_50_) were determined using a four-parameter logistic (4PL) sigmoidal regression model. Relative affinity of 6.3 normalized to the EC_50_ of mPEG_79_-**T**.

| Triblock copolymer | *EC*_50_ [ng mL^-1^] | 6.3 Relative Affinity [%] |
| --- | --- | --- |
| mPEG_79_-**T** | 598 ± 17 | 100.00 |
| $\text{rPEG}_{\text{60}}^{\text{0.25}}$-**T** | 25214 ± 1037 | 2.37 ± 0.12 |
| $\text{rPEG}_{\text{58}}^{\text{0.49}}$-**T** | 116403 ± 5649 | 0.51 ± 0.03 |
| $\text{rPEG}_{\text{42}}^{\text{0.75}}$-**T** | ALOQ^a)^ | n.d./ALOQ^b)^ |
| PGME_34_-**T** | ALOQ^a)^ | n.d./ALOQ^b)^ |

^a)^Above the concentration limit; reported as above the limit of quantification (ALOQ). ^b)^Effectively close to zero but reported as not determined (n.d.) since their *EC*_50_ values were ALOQ.

**3. Supporting Figures**

**3.1 NMR Spectra**


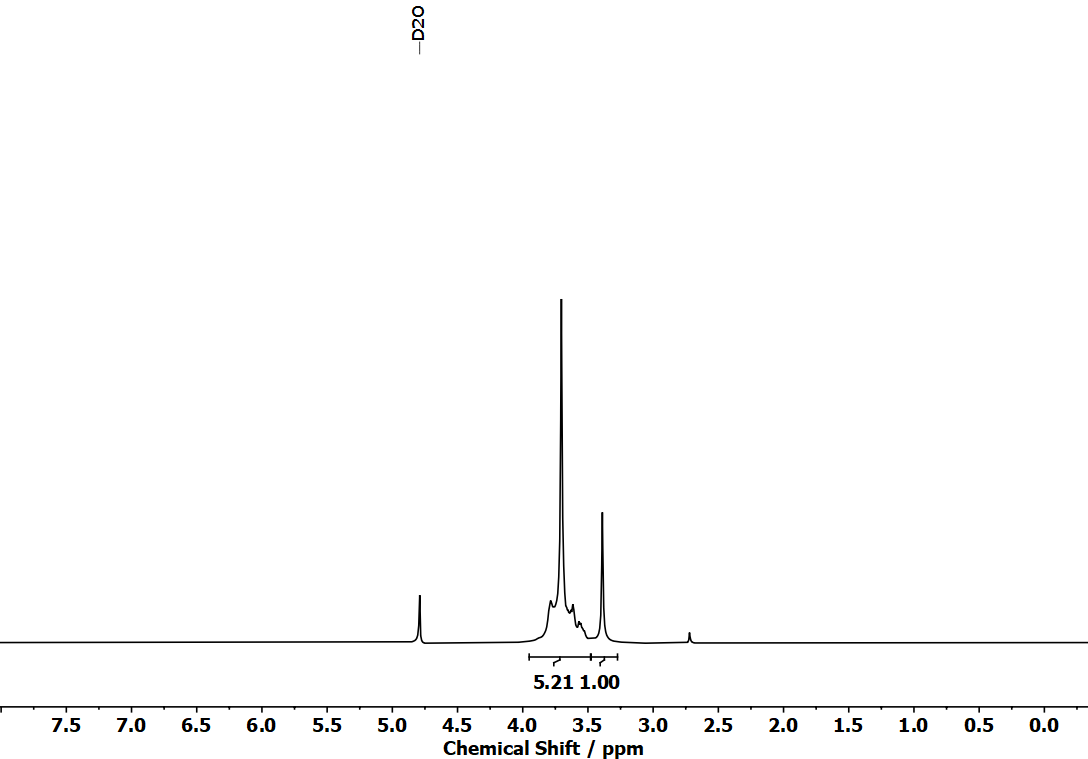


Figure S 1: ^1^H NMR (400 MHz, D_2_O) spectrum of $\text{rPEG}_{\text{60}}^{\text{0.25}}$.


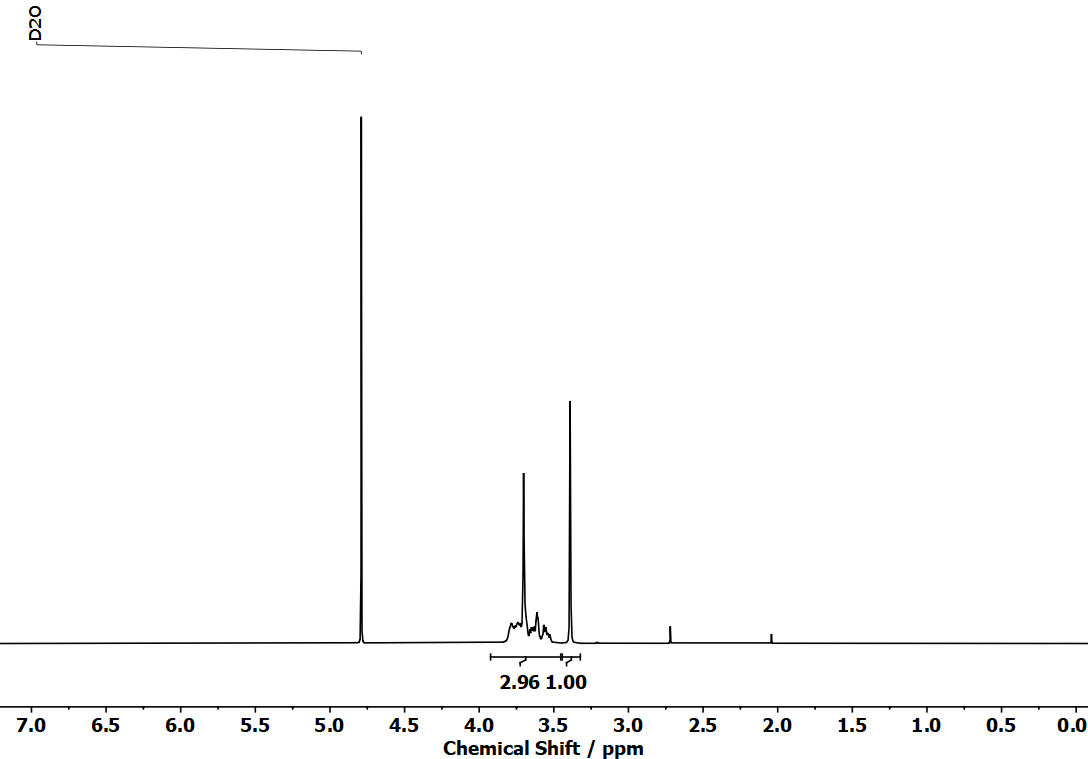


Figure S 2: ^1^H NMR (400 MHz, D_2_O) spectrum of $\text{rPEG}_{\text{58}}^{\text{0.49}}$.


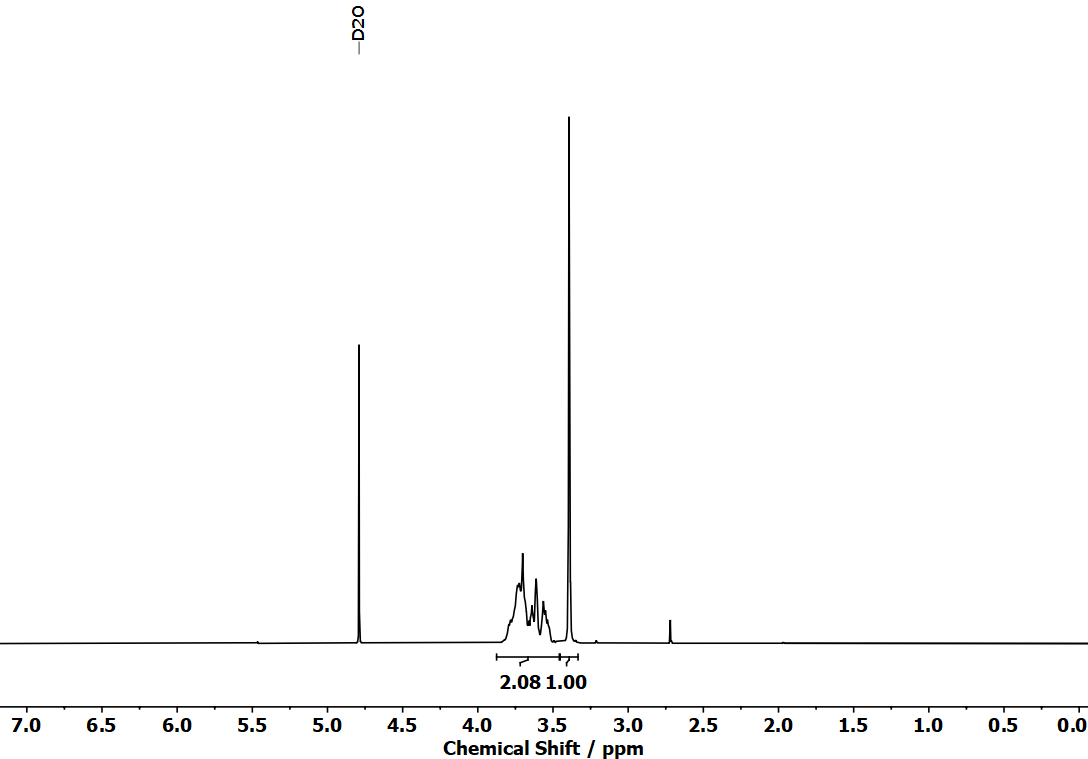


Figure S 3: ^1^H NMR (400 MHz, D_2_O) spectrum of $\text{rPEG}_{\text{42}}^{\text{0.75}}$.


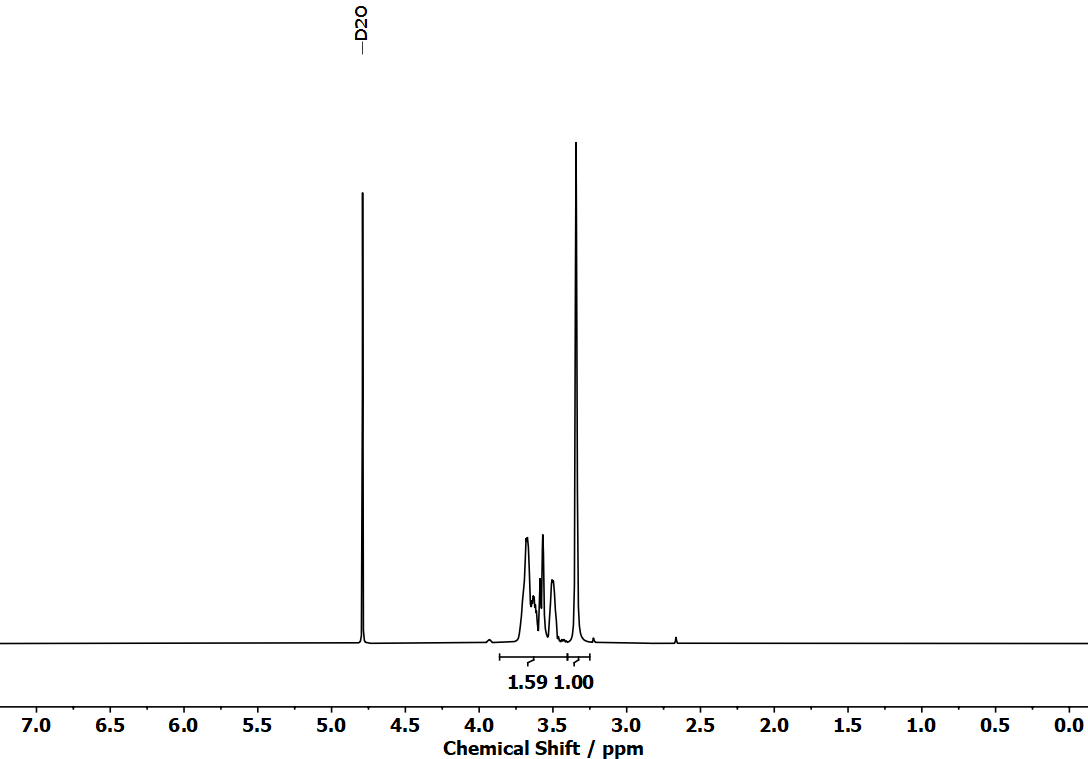


Figure S 4: ^1^H NMR (600 MHz, D_2_O) spectrum of PGME_34_.


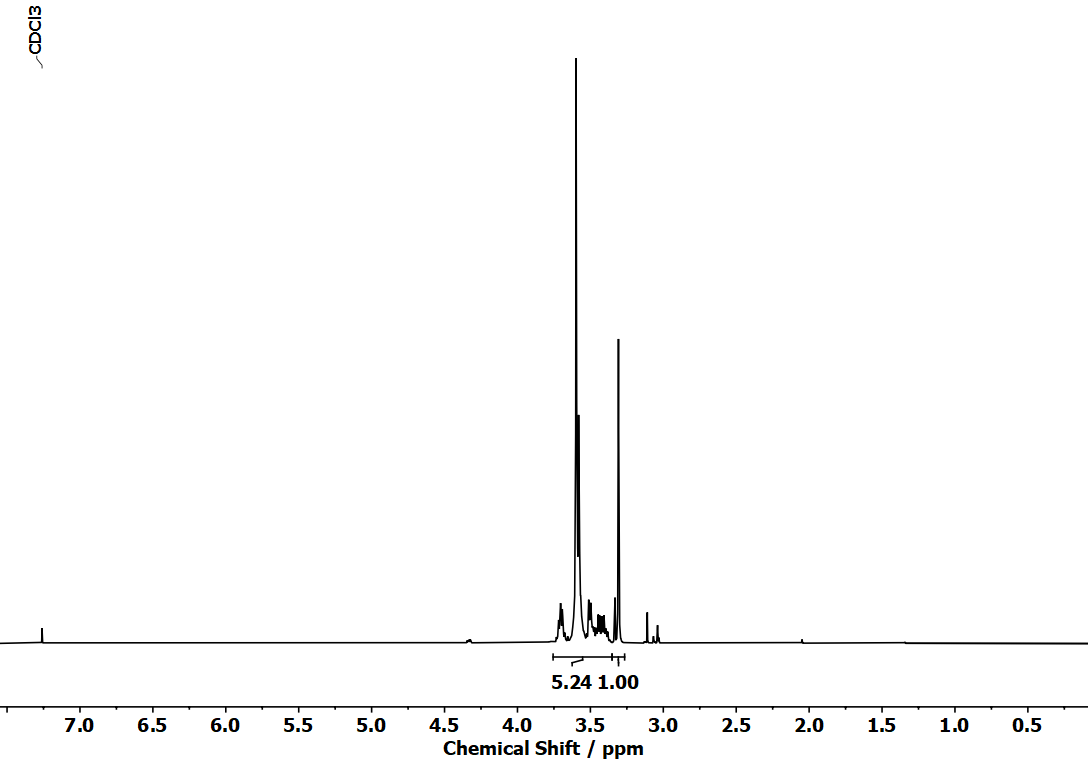


Figure S 5: ^1^H NMR (400 MHz, CD_3_Cl) spectrum of $\text{rPEG}_{\text{60}}^{\text{0.25}}$-Ms.


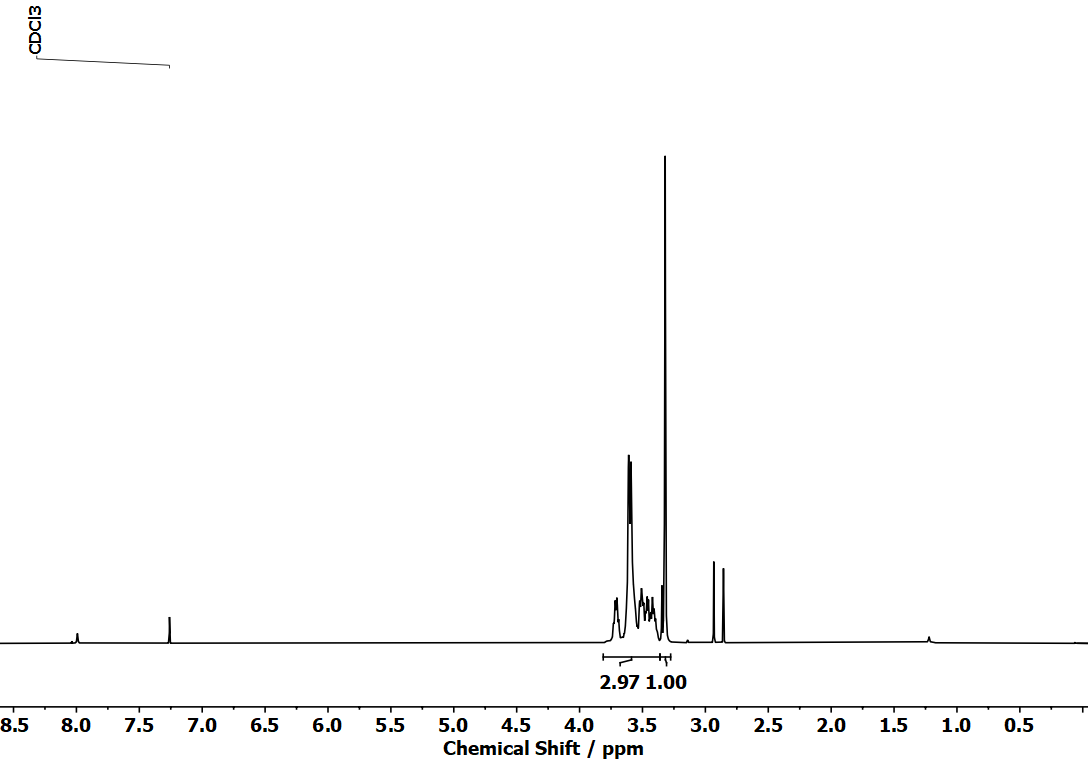


Figure S 6: ^1^H NMR (400 MHz, CD_3_Cl) spectrum of $\text{rPEG}_{\text{58}}^{\text{0.49}}$-Ms.


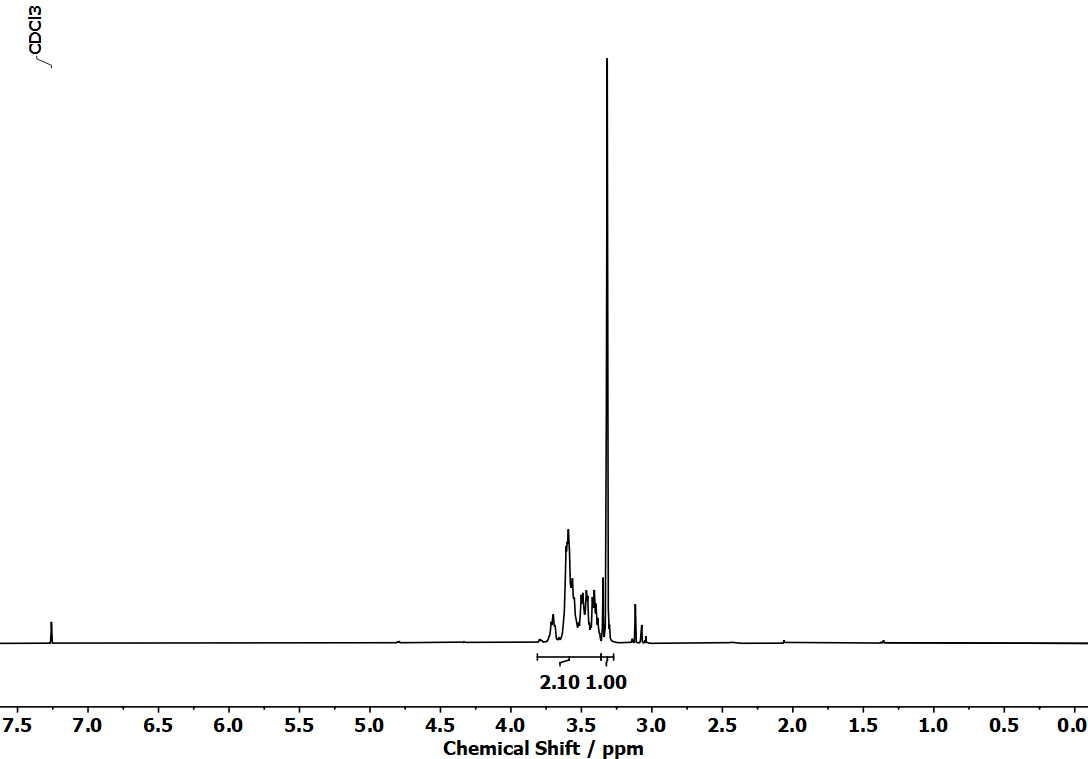


Figure S 7: ^1^H NMR (400 MHz, CD_3_Cl) spectrum of $\text{rPEG}_{\text{42}}^{\text{0.75}}$-Ms.


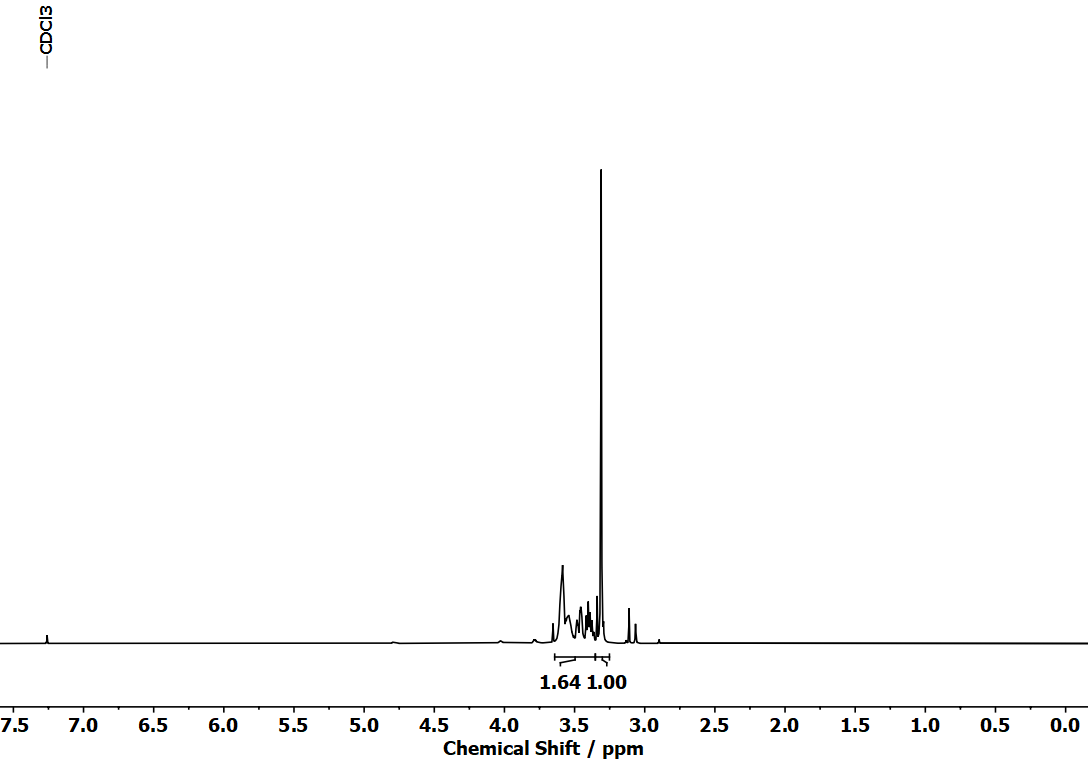


Figure S 8: ^1^H NMR (400 MHz, CD_3_Cl) spectrum of PGME_34_-Ms.


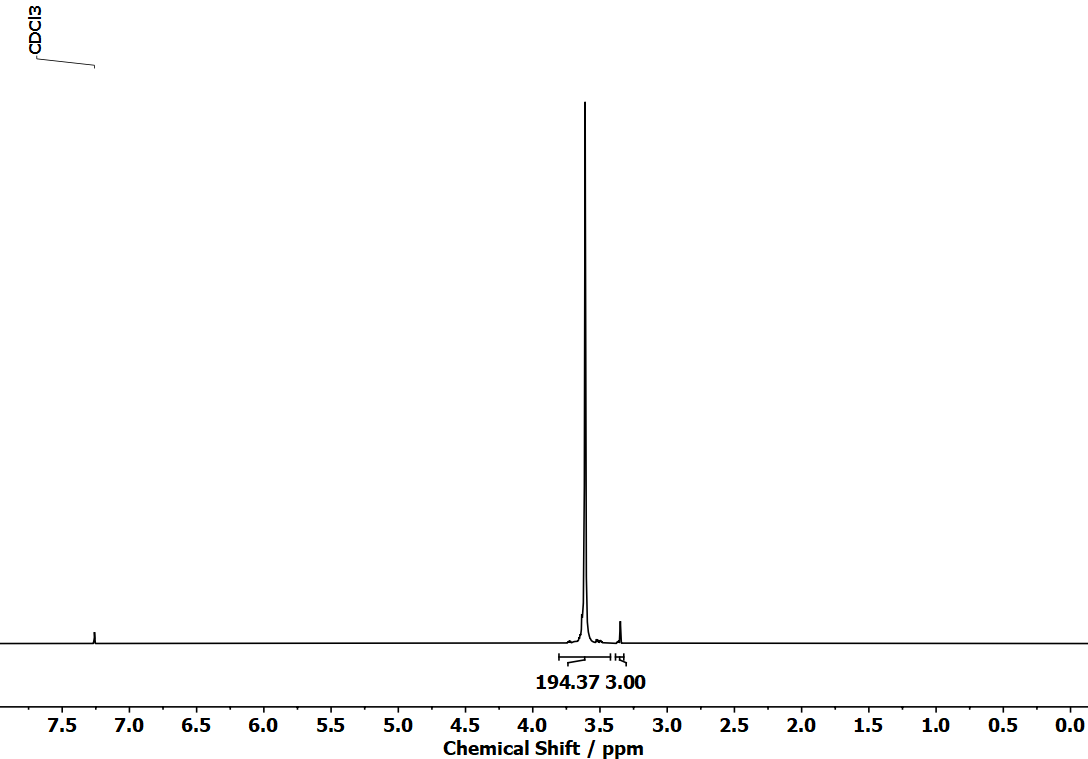


Figure S 9: ^1^H NMR (600 MHz, CDCl_3_) spectrum of the commercially acquired mPEG_79_-N_3_.


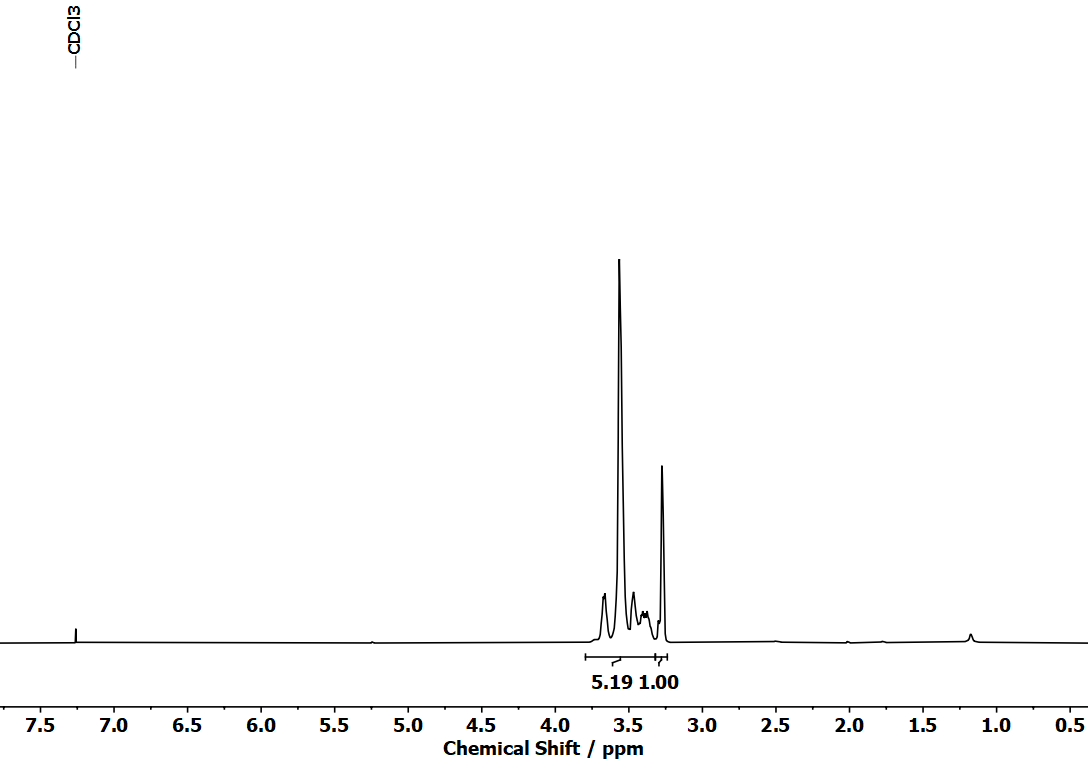


Figure S 10: ^1^H NMR (400 MHz, CD_3_Cl) spectrum of $\text{rPEG}_{\text{60}}^{\text{0.25}}$-N_3_.


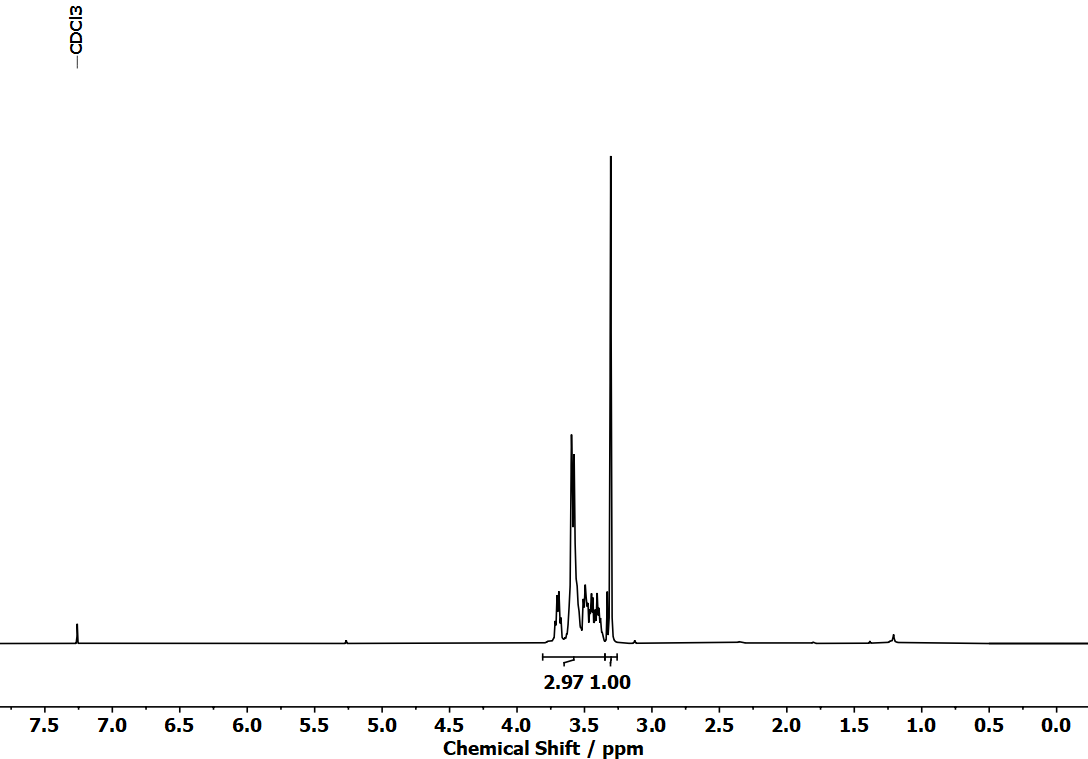


Figure S 11: ^1^H NMR (400 MHz, CD_3_Cl) spectrum of $\text{rPEG}_{\text{58}}^{\text{0.49}}$-N_3_.


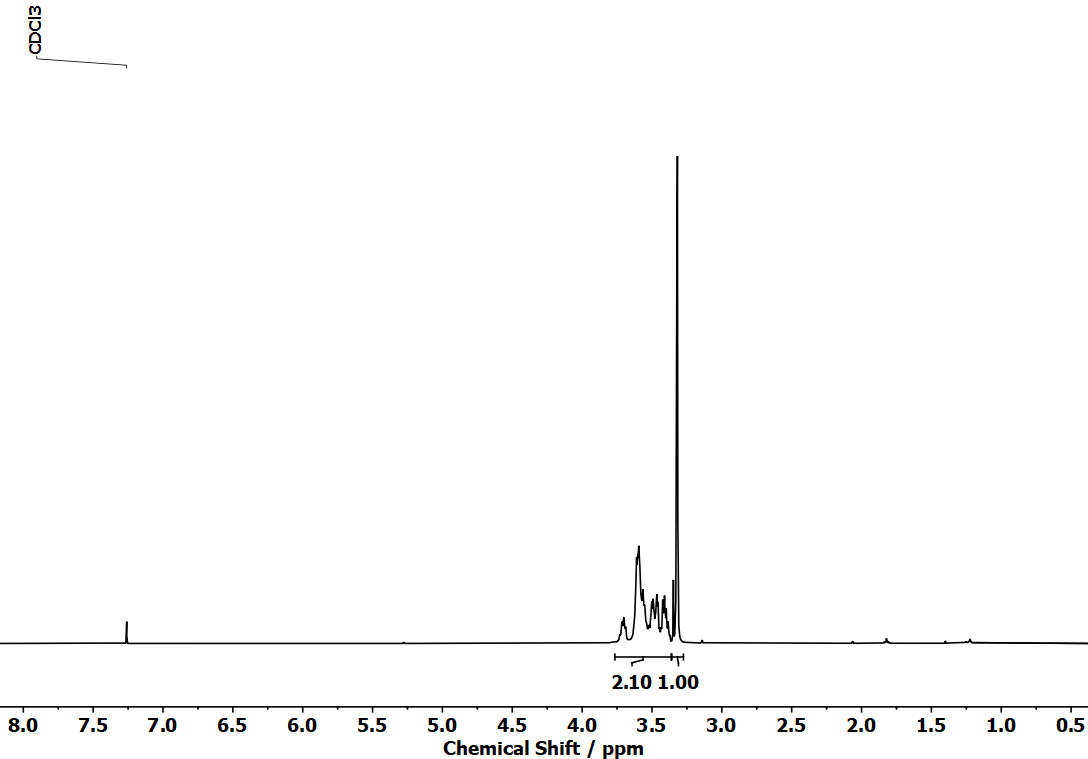


Figure S 12: ^1^H NMR (400 MHz, CD_3_Cl) spectrum of $\text{rPEG}_{\text{42}}^{\text{0.75}}$-N_3_.


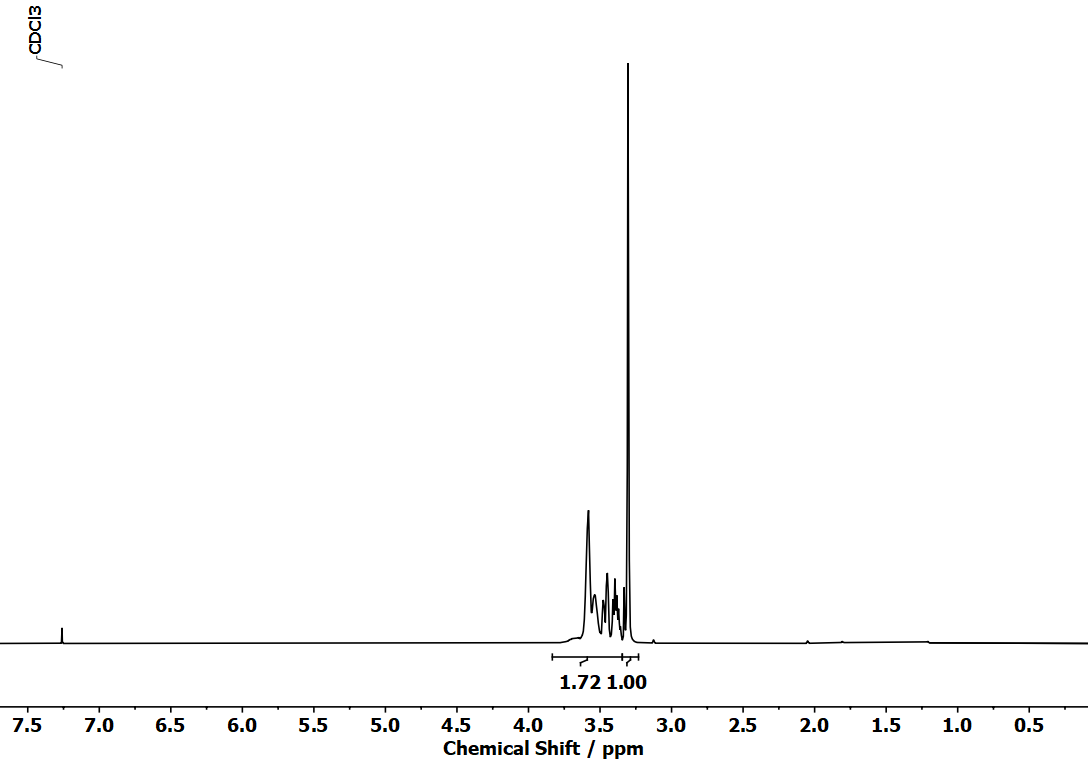


Figure S 13: ^1^H NMR (400 MHz, CD_3_Cl) spectrum of PGME_34_-N_3_.


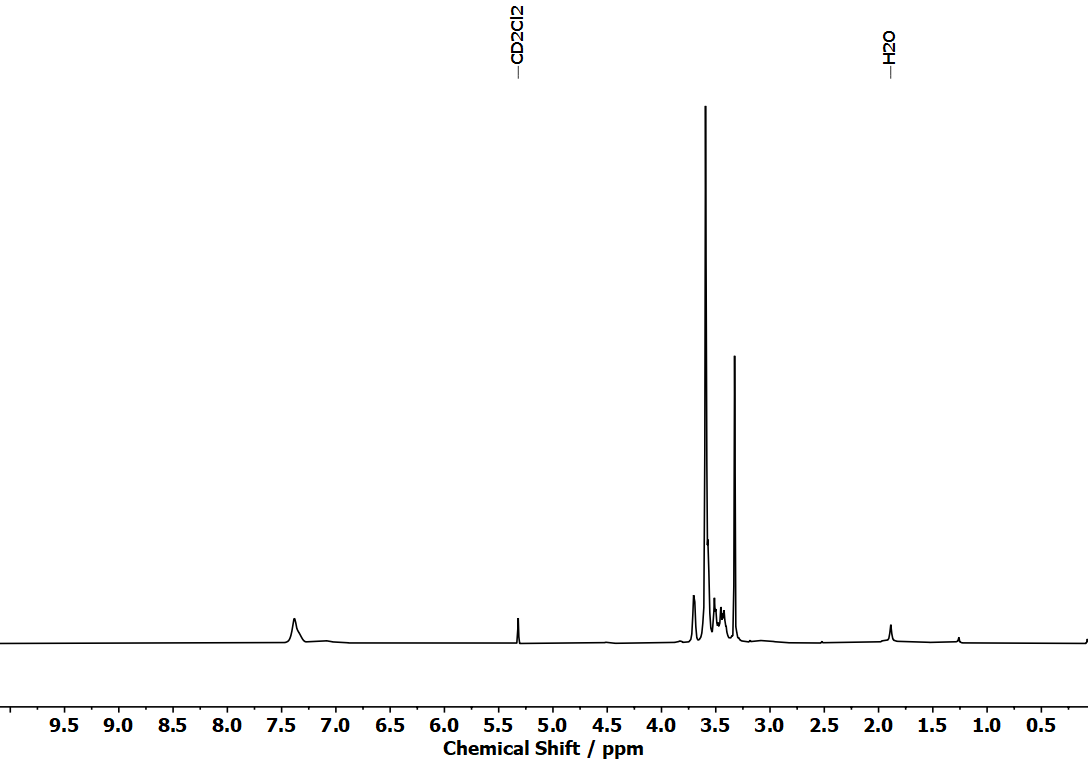


Figure S 14: ^1^H NMR (500 MHz, CD_2_Cl_2_) spectrum of PPheOzi_16_-dialkyne.


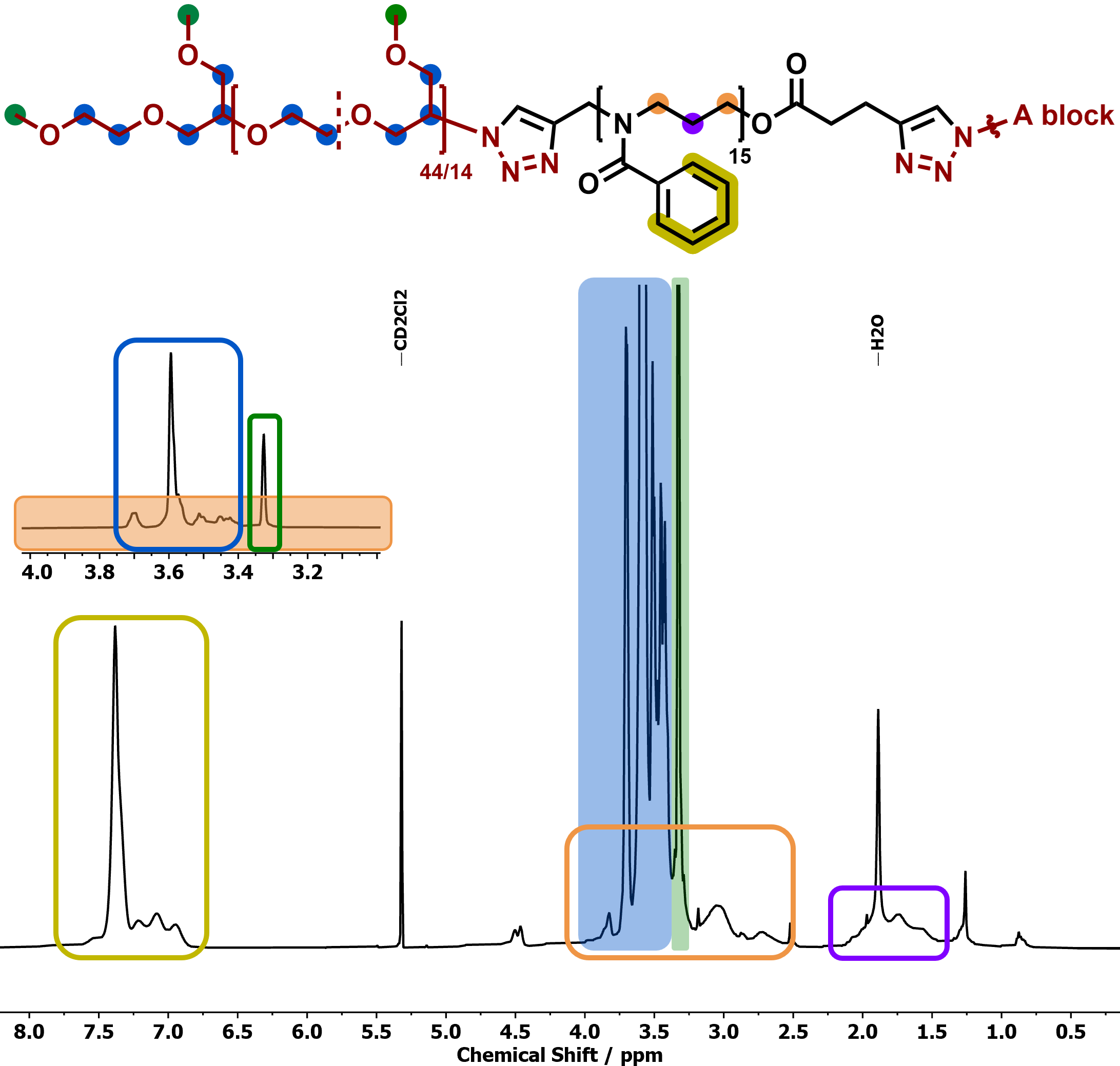


Figure S 15: ^1^H NMR (500 MHz, CD_2_Cl_2_) spectrum of $\text{rPEG}_{\text{60}}^{\text{0.25}}$-*b*-PPheOzi_16_-*b*-$\text{rPEG}_{\text{60}}^{\text{0.25}}$ ($\text{rPEG}_{\text{60}}^{\text{0.25}}$-**T**) with exemplary signal assignment.


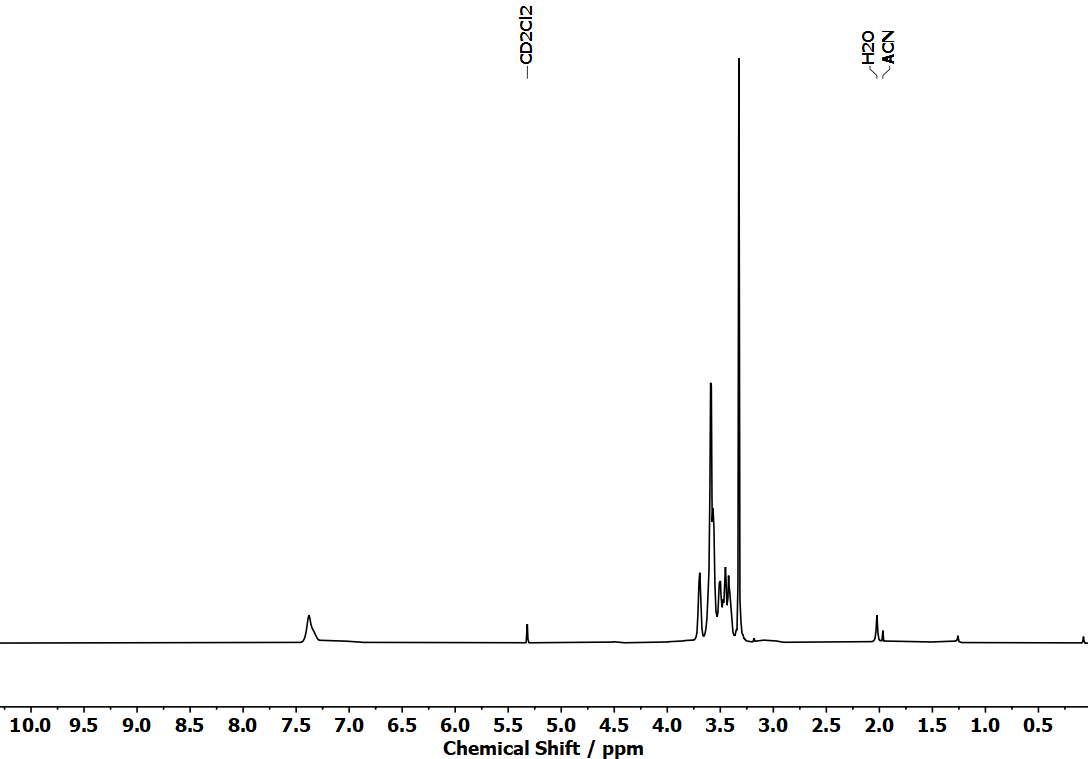


Figure S 16: ^1^H NMR (500 MHz, CD_2_Cl_2_) spectrum of $\text{rPEG}_{\text{58}}^{\text{0.49}}$-*b*-PPheOzi_16_-*b*-$\text{rPEG}_{\text{58}}^{\text{0.49}}$ ($\text{rPEG}_{\text{58}}^{\text{0.49}}$-**T**).


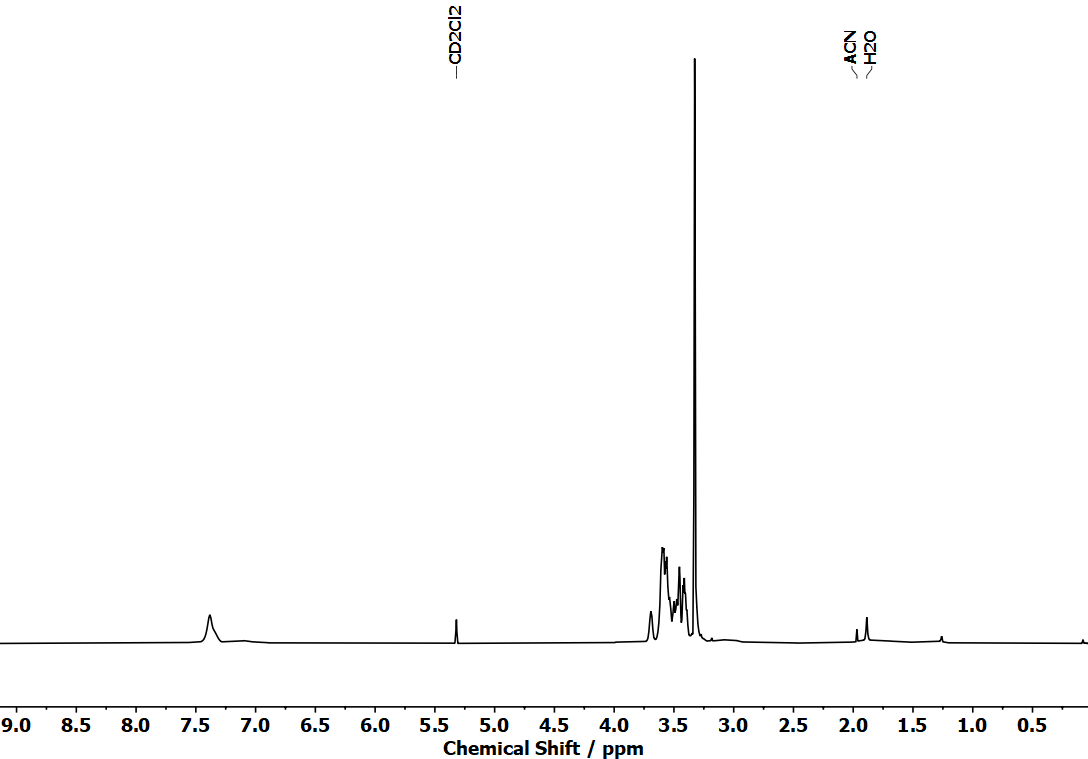


Figure S 17: ^1^H NMR (500 MHz, CD_2_Cl_2_) spectrum of $\text{rPEG}_{\text{42}}^{\text{0.75}}$-*b*-PPheOzi_16_-*b*-$\text{rPEG}_{\text{42}}^{\text{0.75}}$ ($\text{rPEG}_{\text{42}}^{\text{0.75}}$-**T**).


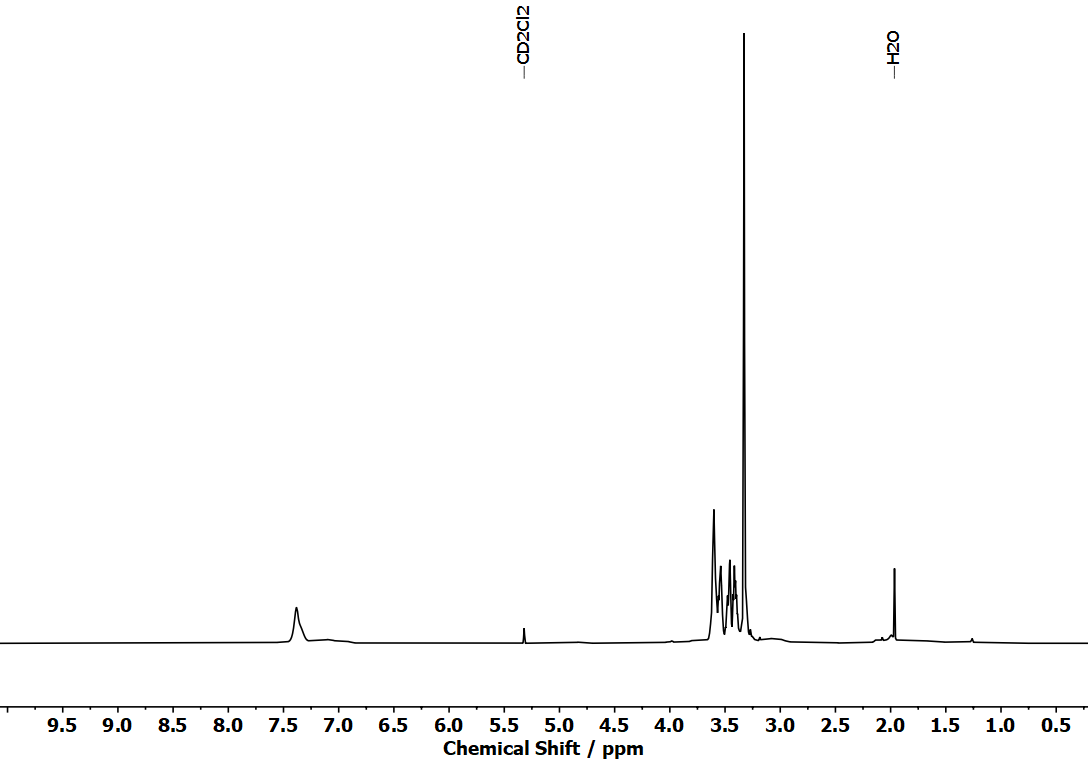


Figure S 18: ^1^H NMR (500 MHz, CD_2_Cl_2_) spectrum of PGME_34_-*b*-PPheOzi_16_-*b*-PGME_34_ (PGME_34_-**T**).


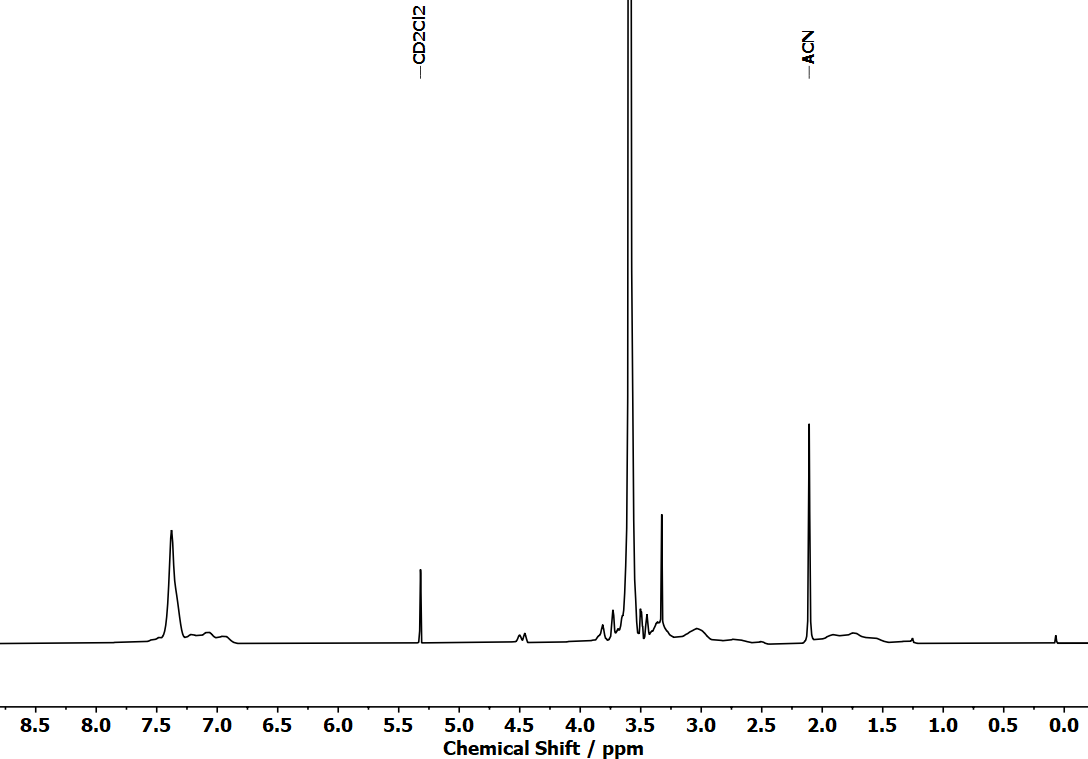


Figure S 19: ^1^H NMR (500 MHz, CD_2_Cl_2_) spectrum of mPEG_79_-b-PPheOzi_16_-b-mPEG_79_ (mPEG_79_-**T**).

**3.2 MALDI-TOF MS**


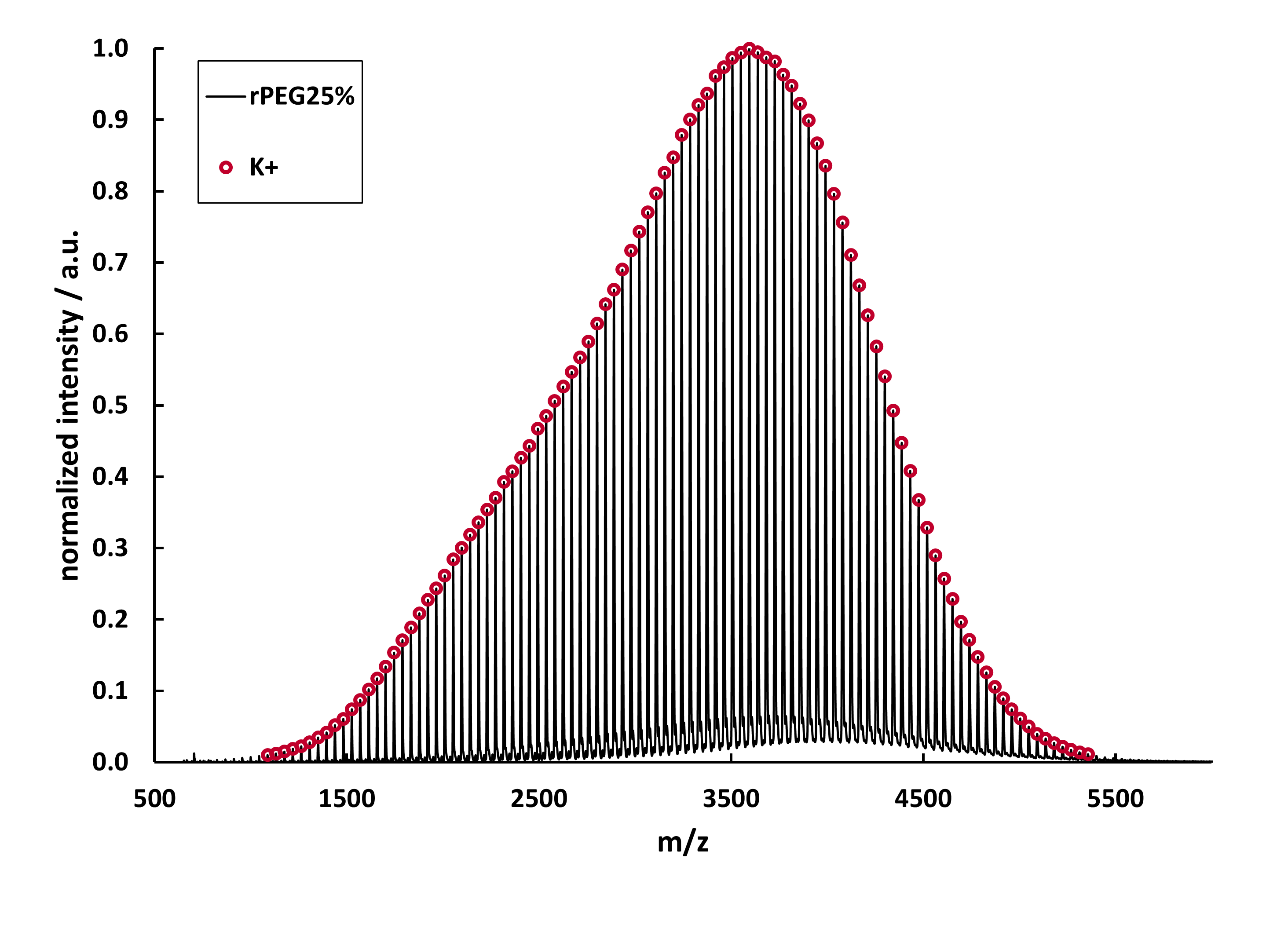


Figure S 20: MALDI-TOF MS (DCTB, KTFA, linear modus) spectrum of $\text{rPEG}_{\text{60}}^{\text{0.25}}$.


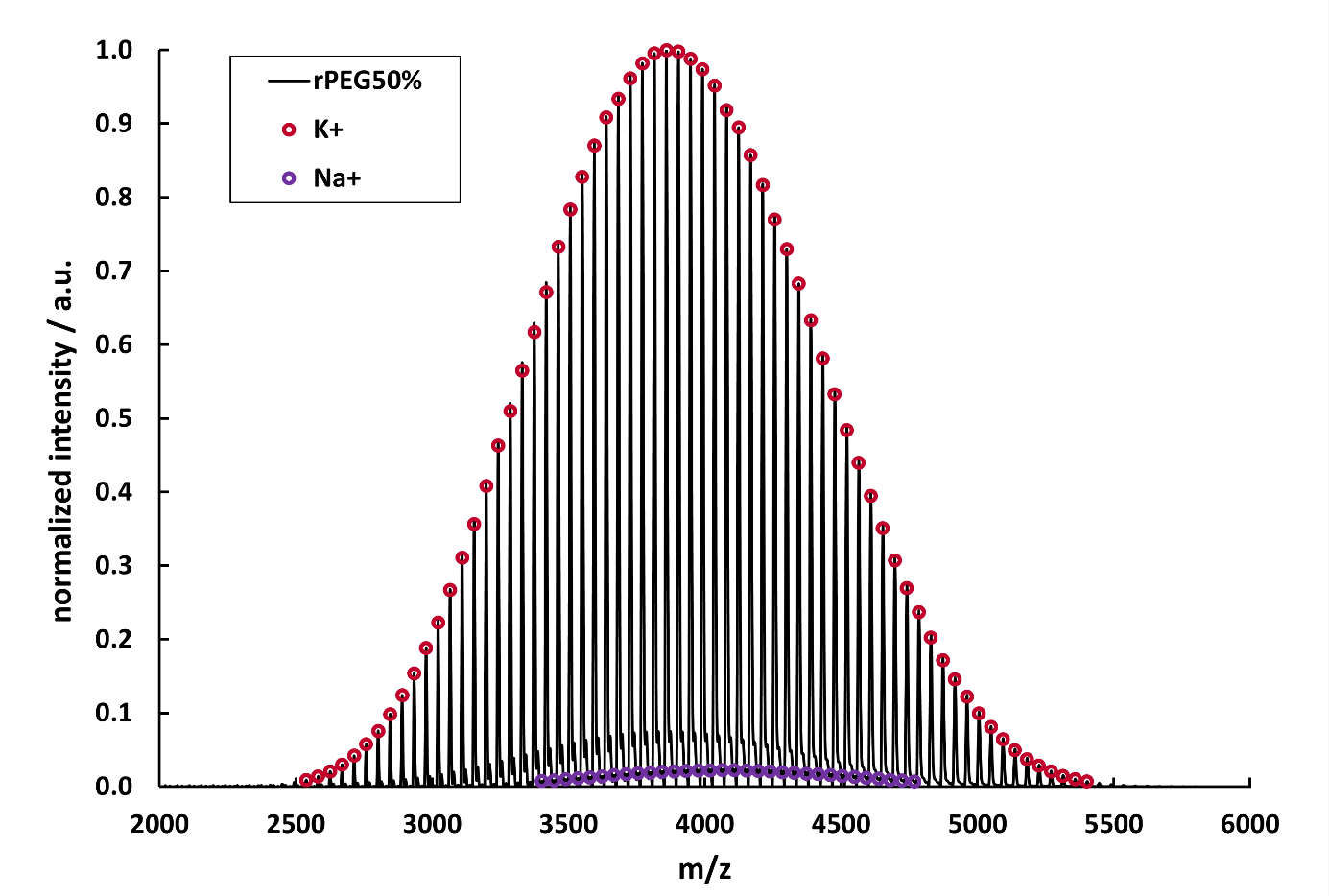


Figure S 21: MALDI-TOF MS (DCTB, KTFA, linear modus) spectrum of $\text{rPEG}_{\text{58}}^{\text{0.49}}$.


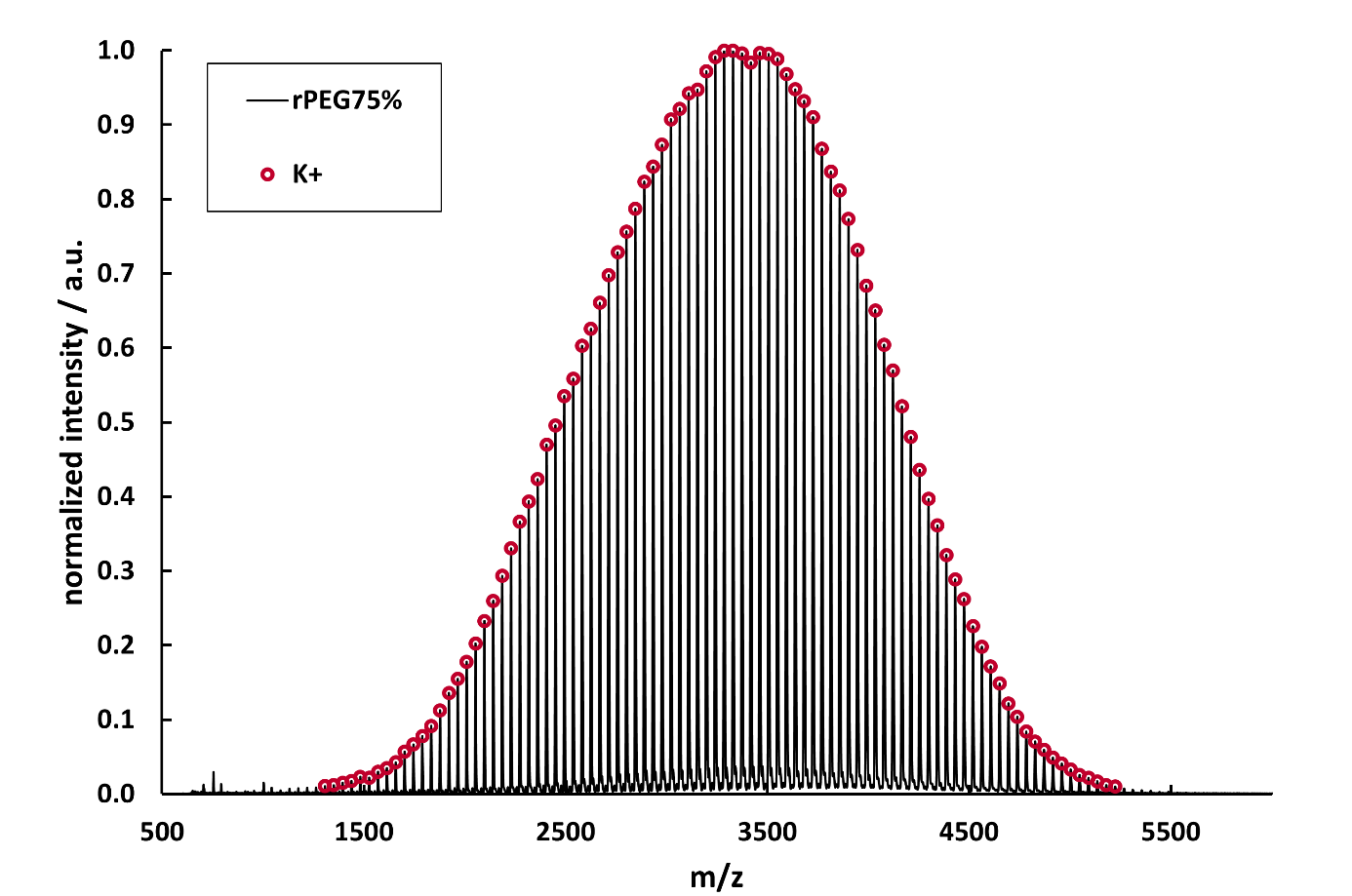


Figure S 22: MALDI-TOF MS (DCTB, KTFA, linear modus) spectrum of $\text{rPEG}_{\text{42}}^{\text{0.75}}$.


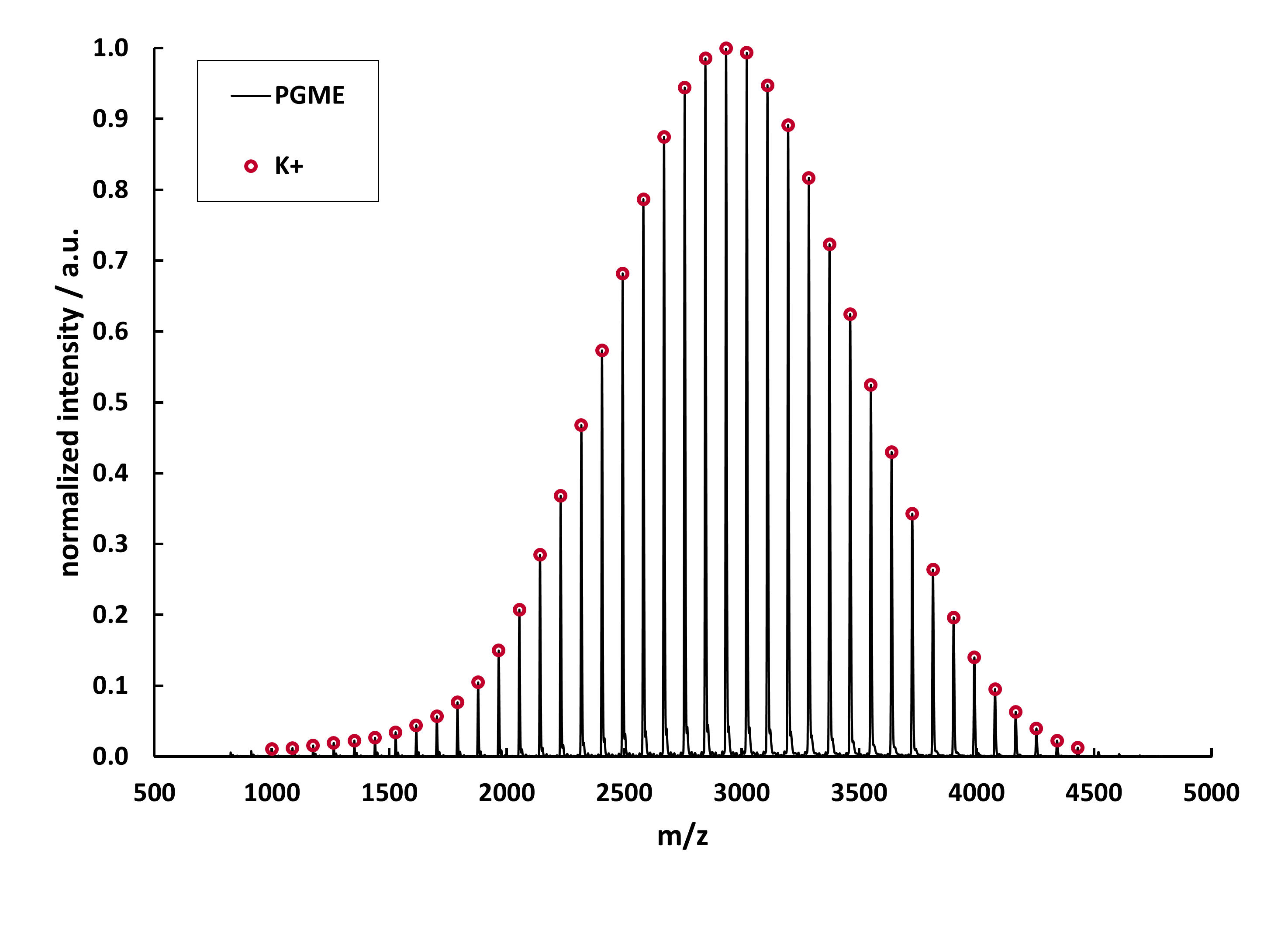


Figure S 23: MALDI-TOF MS (DCTB, KTFA, linear modus) spectrum of PGME_34_.


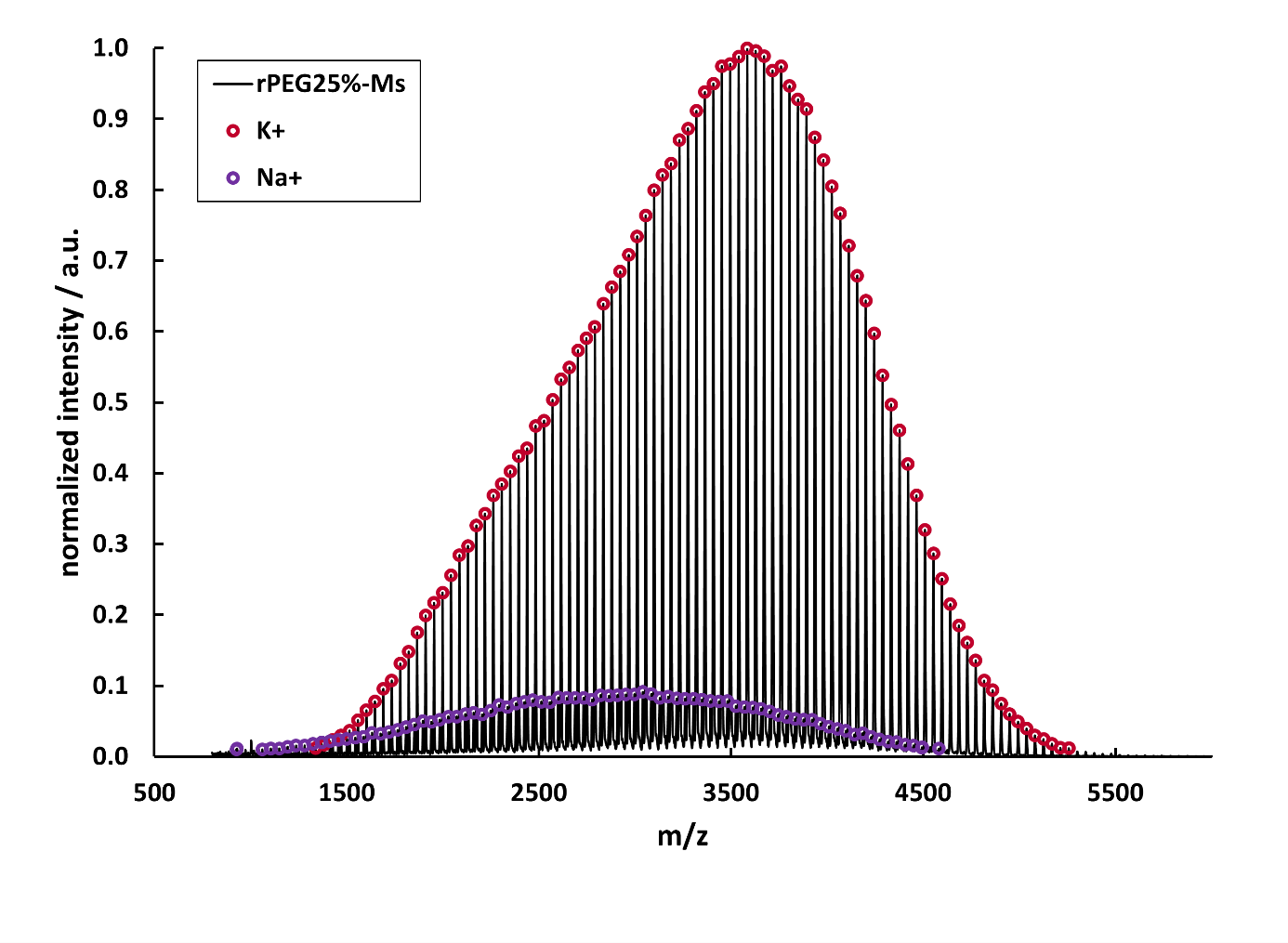


Figure S 24: MALDI-TOF MS (DCTB, KTFA, linear modus) spectrum of $\text{rPEG}_{\text{60}}^{\text{0.25}}$-Ms.


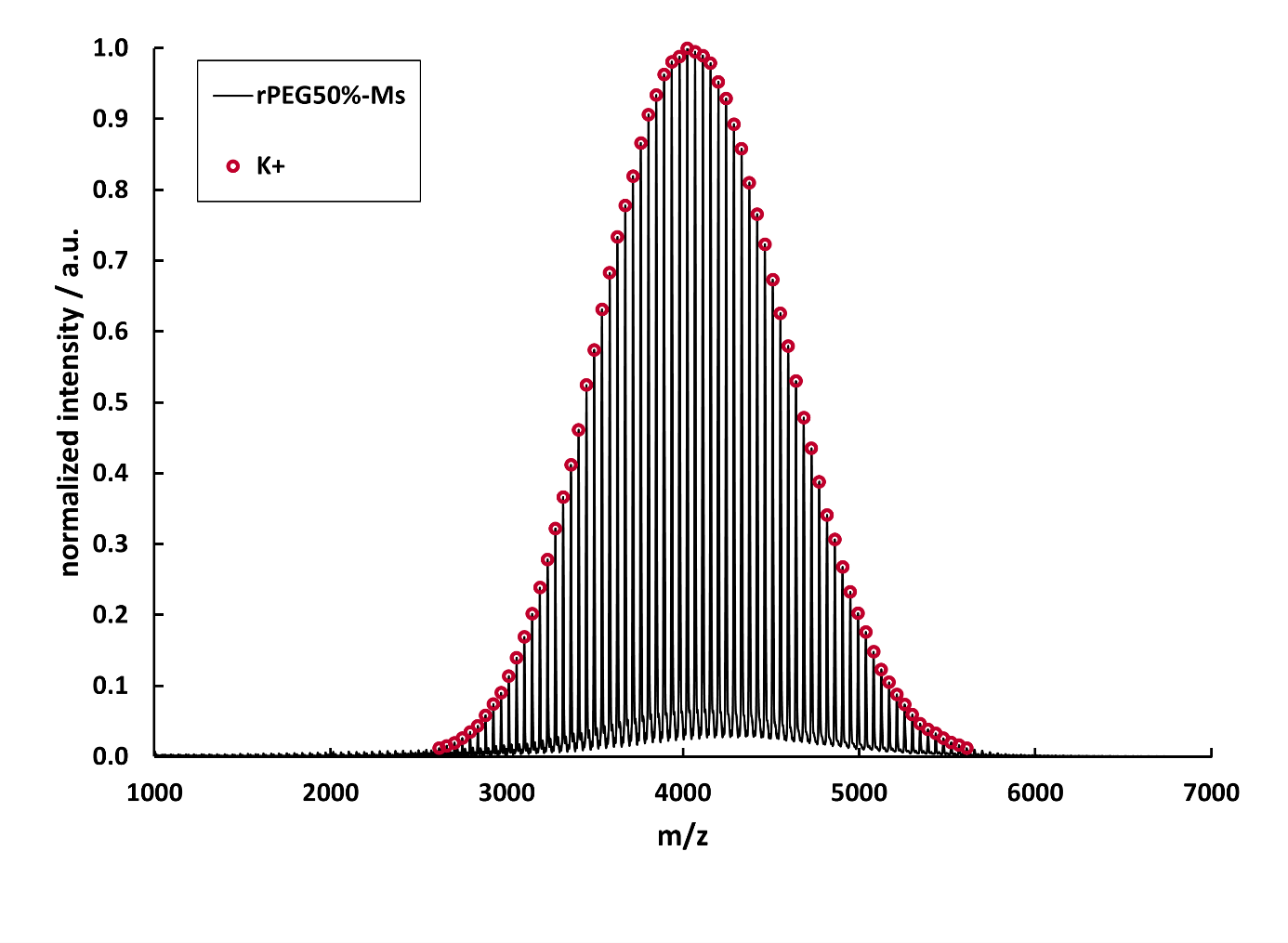


Figure S 25: MALDI-TOF MS (DCTB, KTFA, linear modus) spectrum of $\text{rPEG}_{\text{58}}^{\text{0.49}}$-Ms.


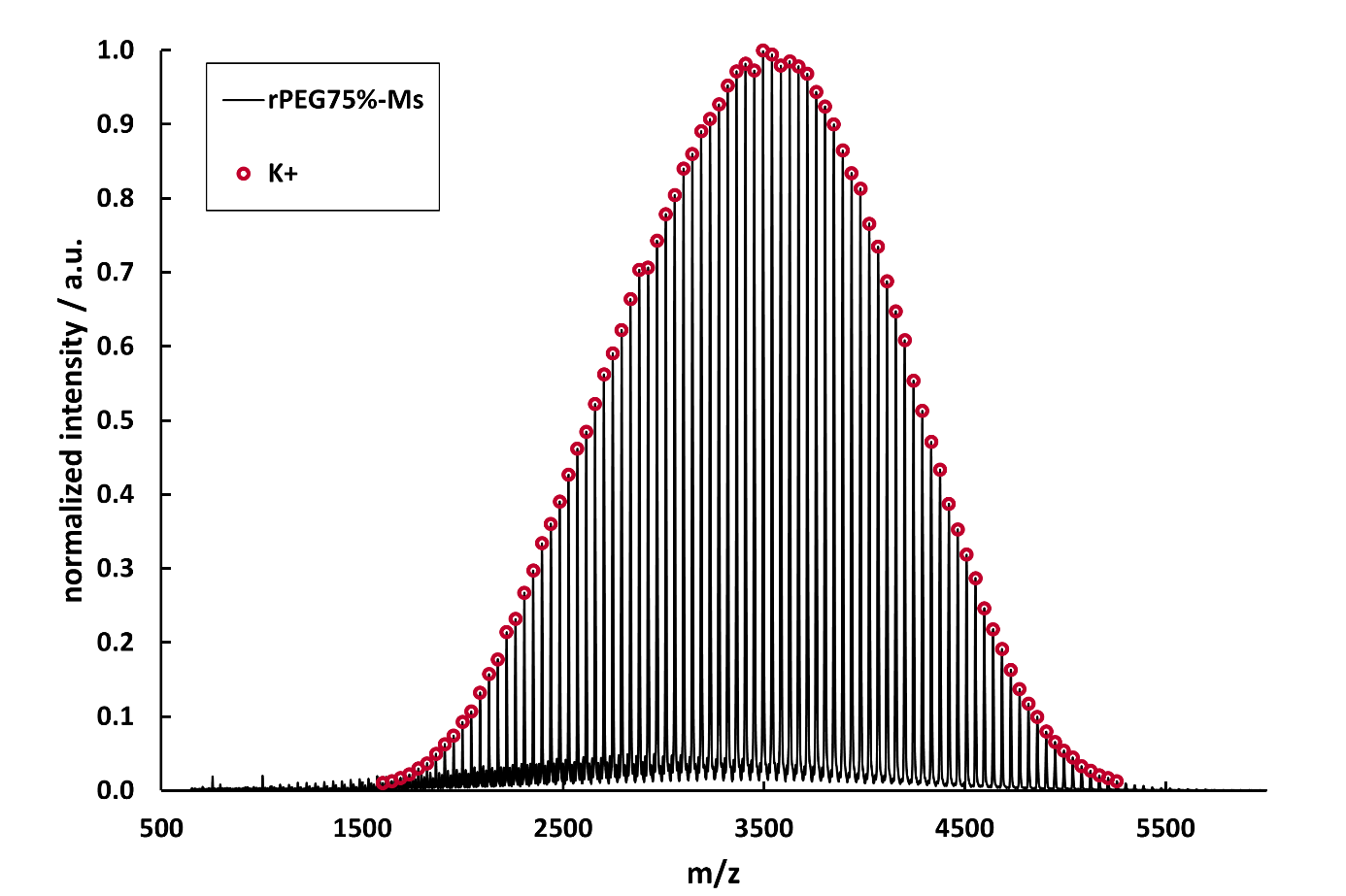


Figure S 26: MALDI-TOF MS (DCTB, KTFA, linear modus) spectrum of $\text{rPEG}_{\text{42}}^{\text{0.75}}$-Ms.


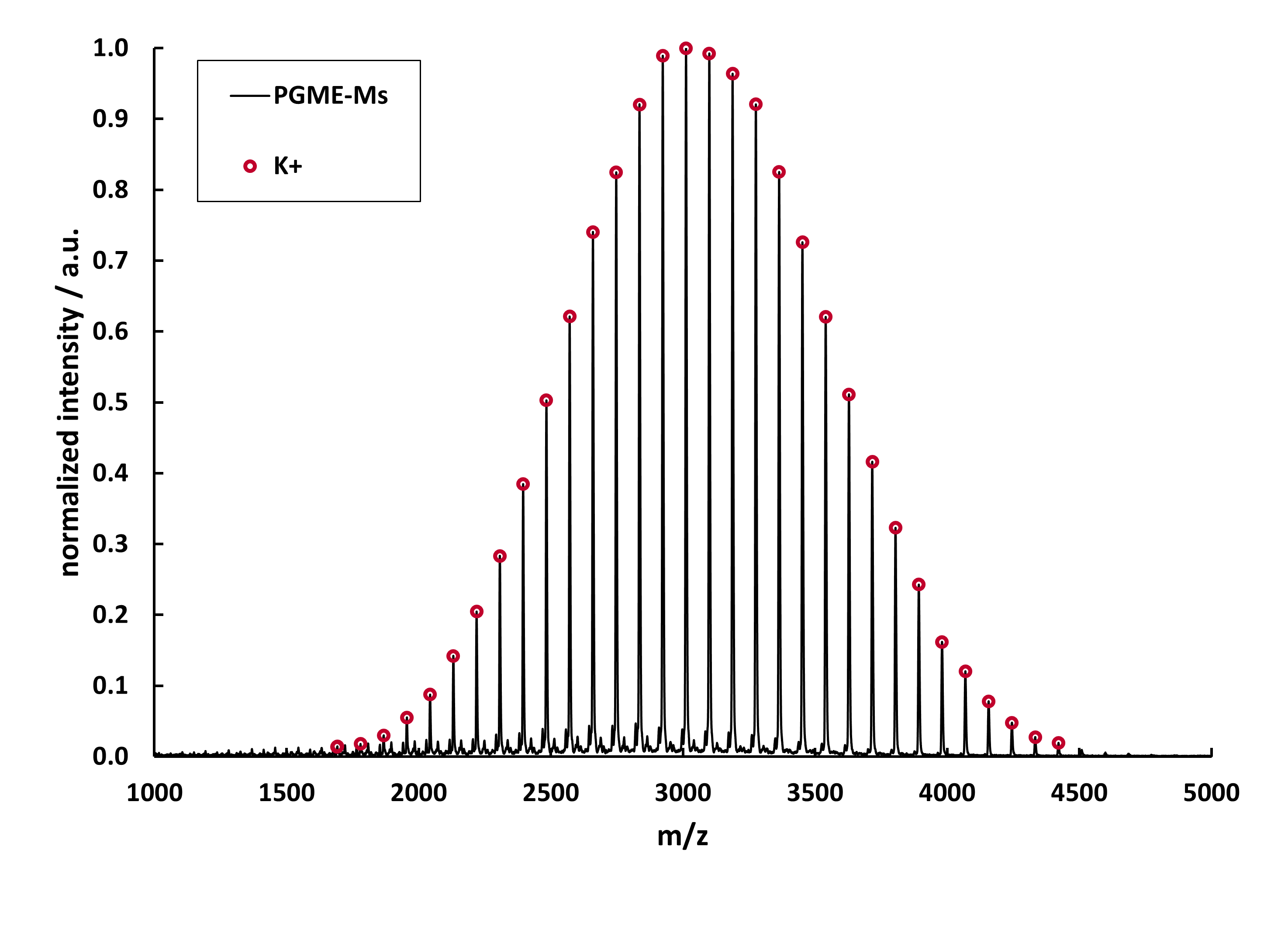


Figure S 27: MALDI-TOF MS (DCTB, KTFA, linear modus) spectrum of PGME_34_-Ms.


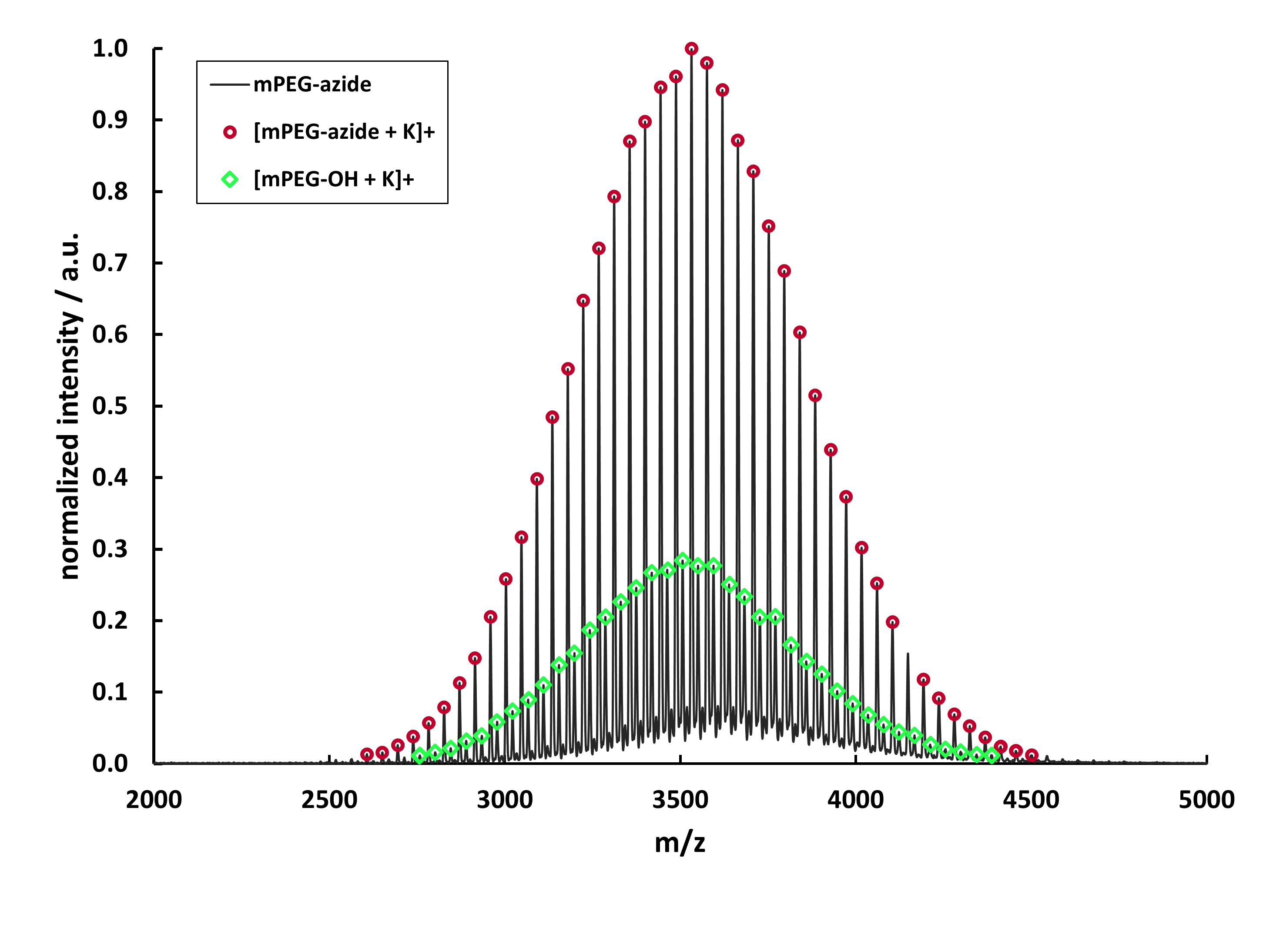


Figure S 28*:* MALDI-TOF MS (DCTB, KTFA, linear modus) spectrum of the commercially acquired mPEG_79_-N_3_.


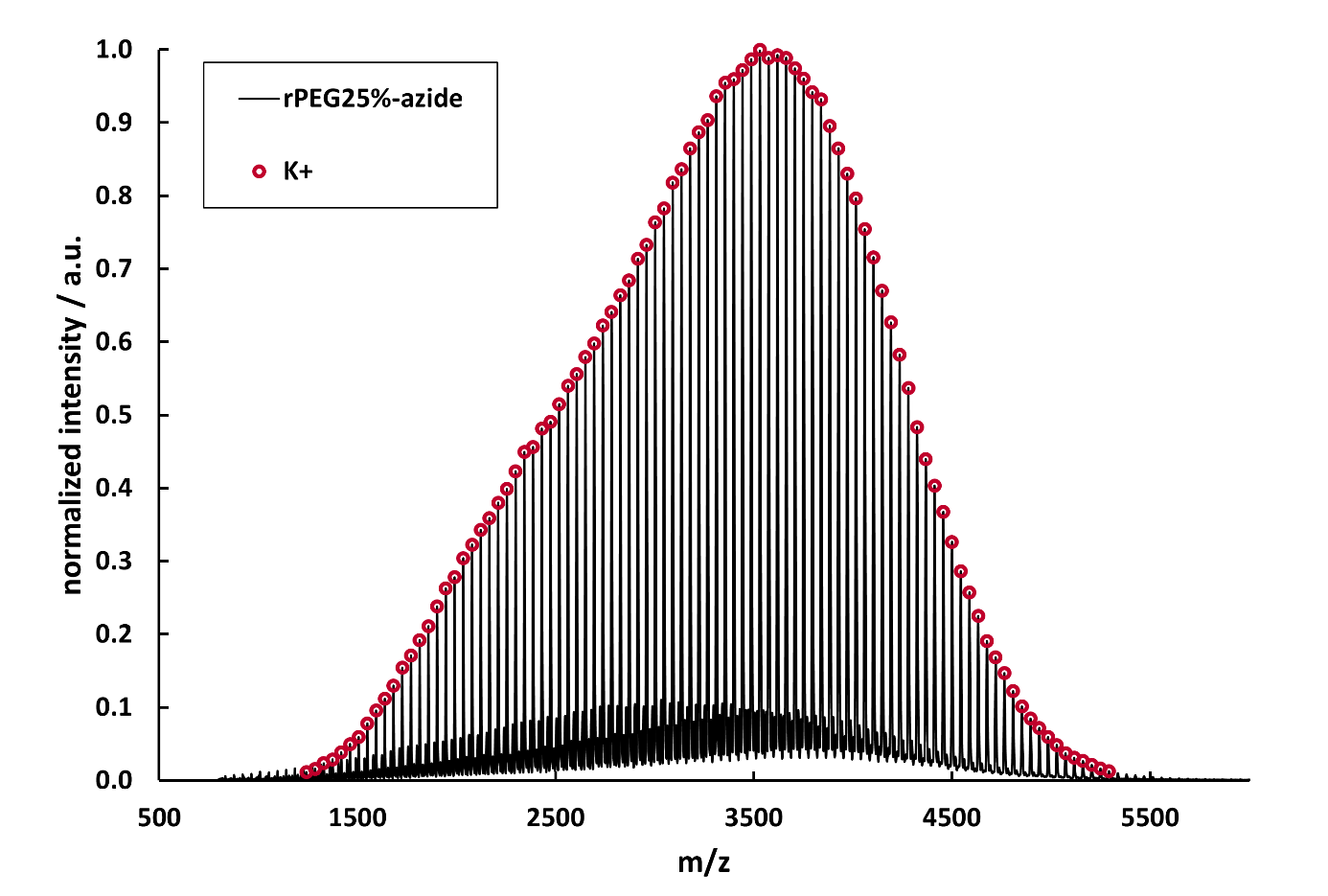


Figure S 29: MALDI-TOF MS (DCTB, KTFA, linear modus) spectrum of $\text{rPEG}_{\text{60}}^{\text{0.25}}$-N_3_.


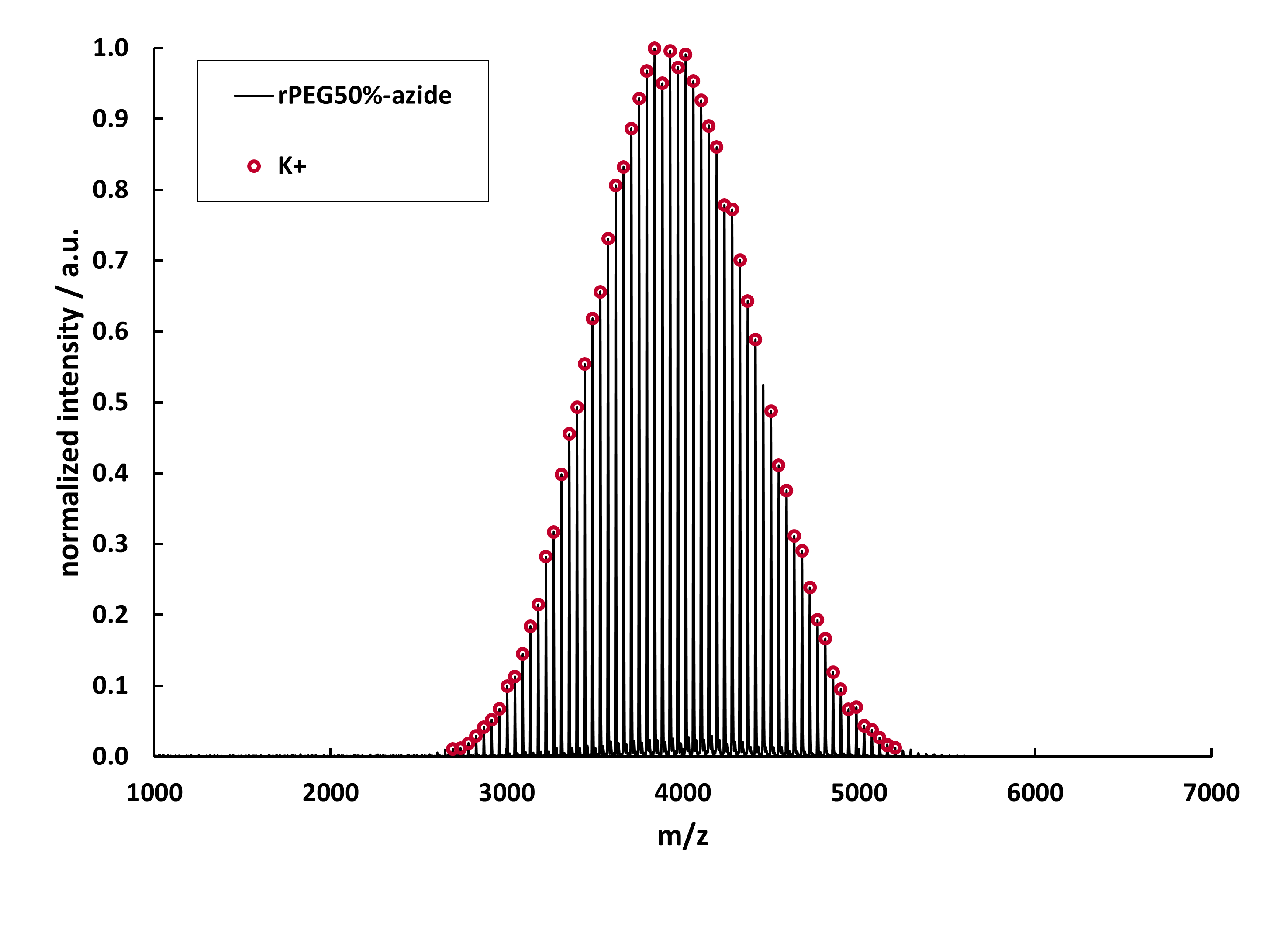


Figure S 30: MALDI-TOF MS (DCTB, KTFA, linear modus) spectrum of $\text{rPEG}_{\text{58}}^{\text{0.49}}$-N_3_.


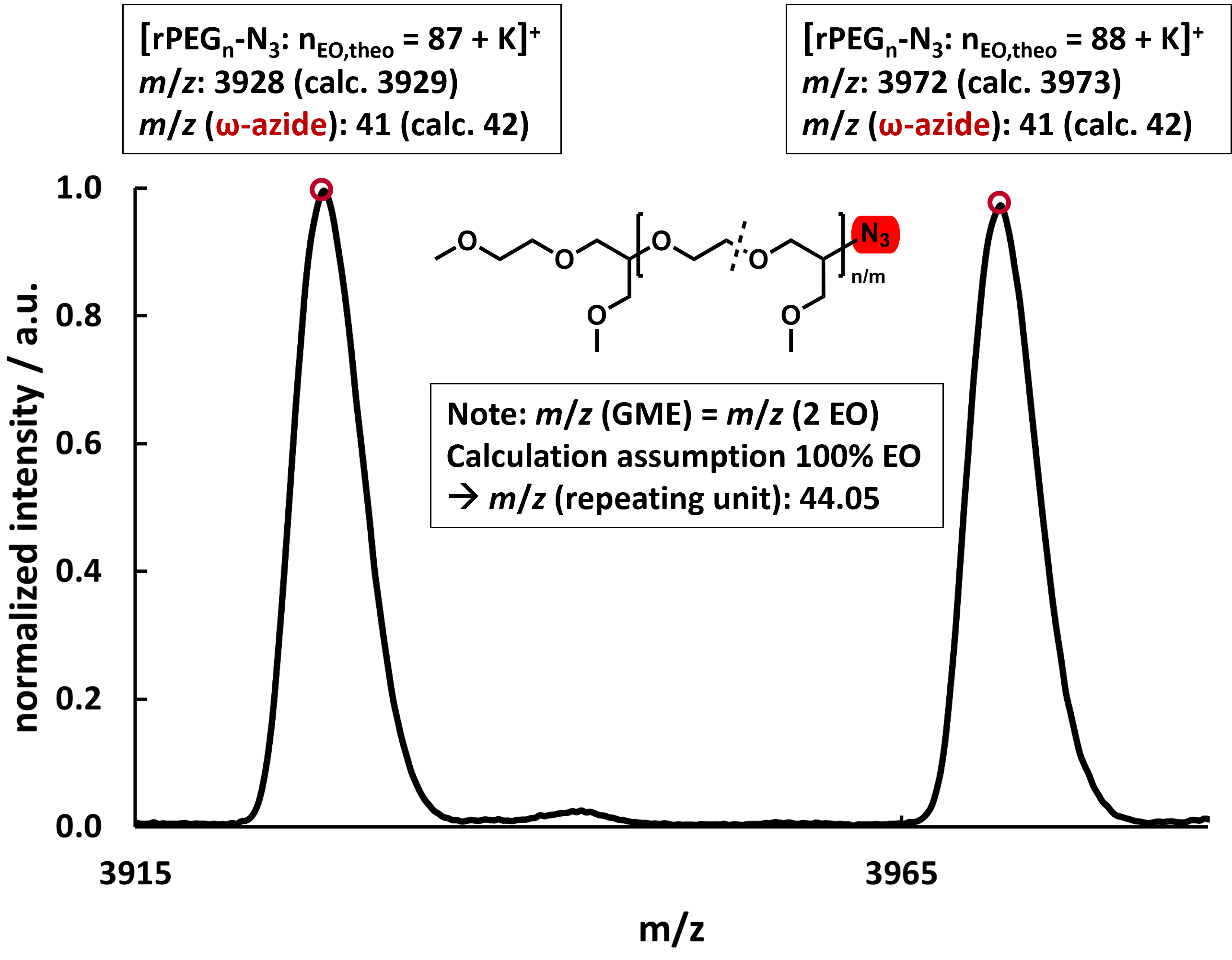


Figure S 31: MALDI-TOF MS (DCTB, KTFA, linear modus) zoom in spectrum of $\text{rPEG}_{\text{58}}^{\text{0.49}}$-N_3_ (Figure S 30). Visualized are the determined and calculated m/z values assigned to azide species. Note that as GME is an isomer of two EO units, the calculation is simplified using a 100% EO assumption, resulting in a simplified m/z value of 44.05 for the repeating unit.


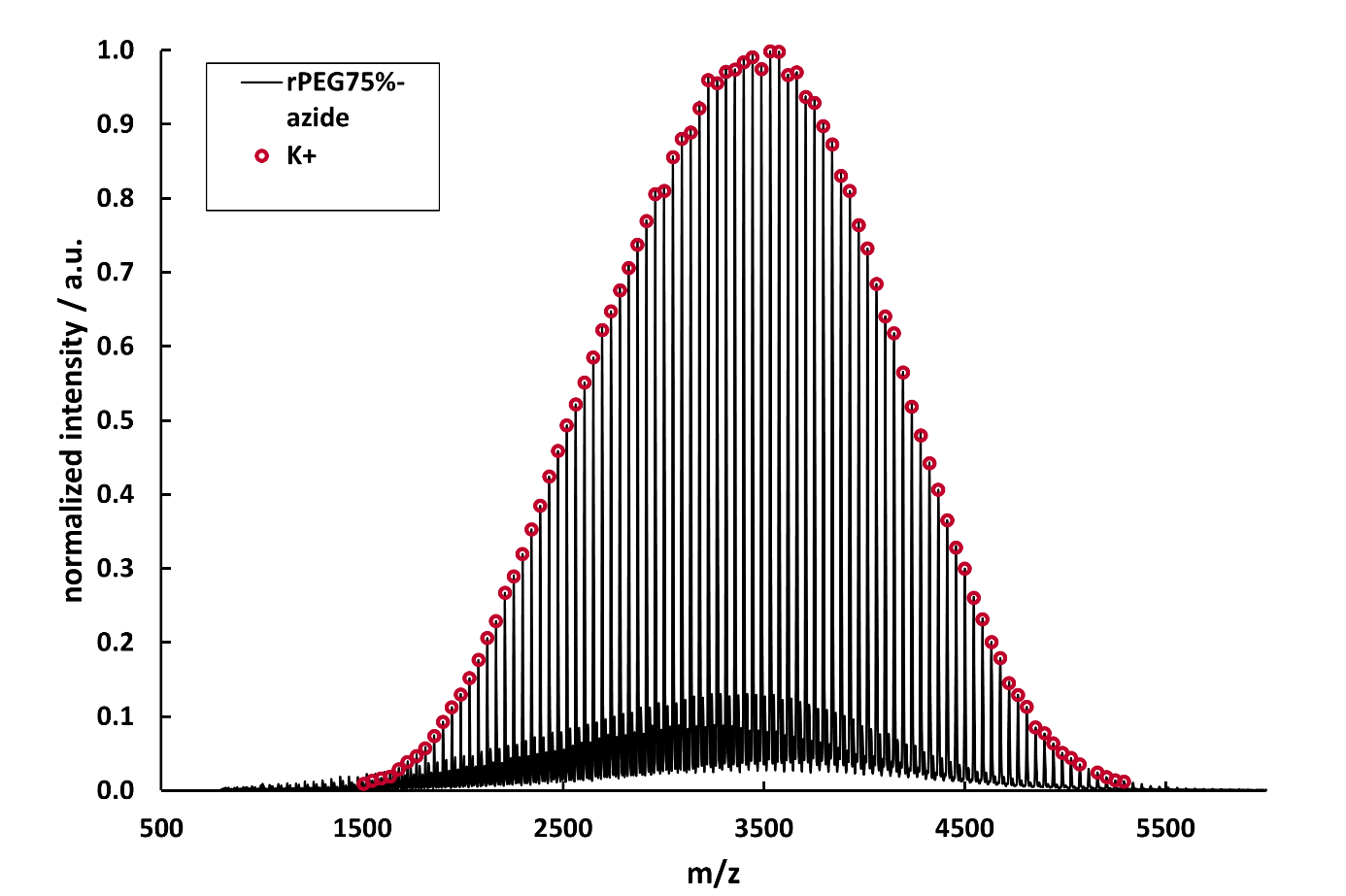


Figure S 32: MALDI-TOF MS (DCTB, KTFA, linear modus) spectrum of $\text{rPEG}_{\text{42}}^{\text{0.75}}$-N_3_.


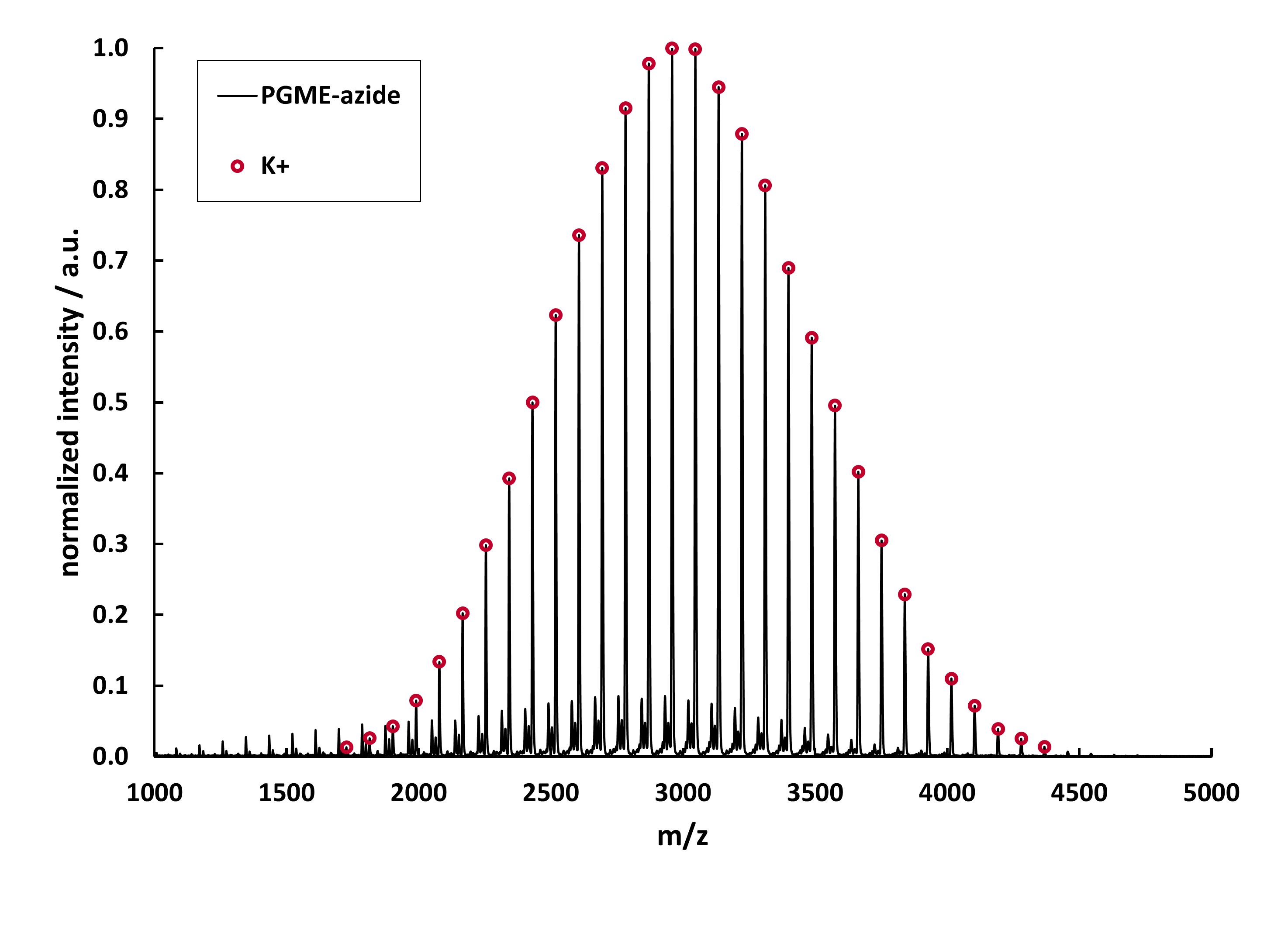


Figure S 33: MALDI-TOF MS (DCTB, KTFA, linear modus) spectrum of PGME_34_-N_3_.


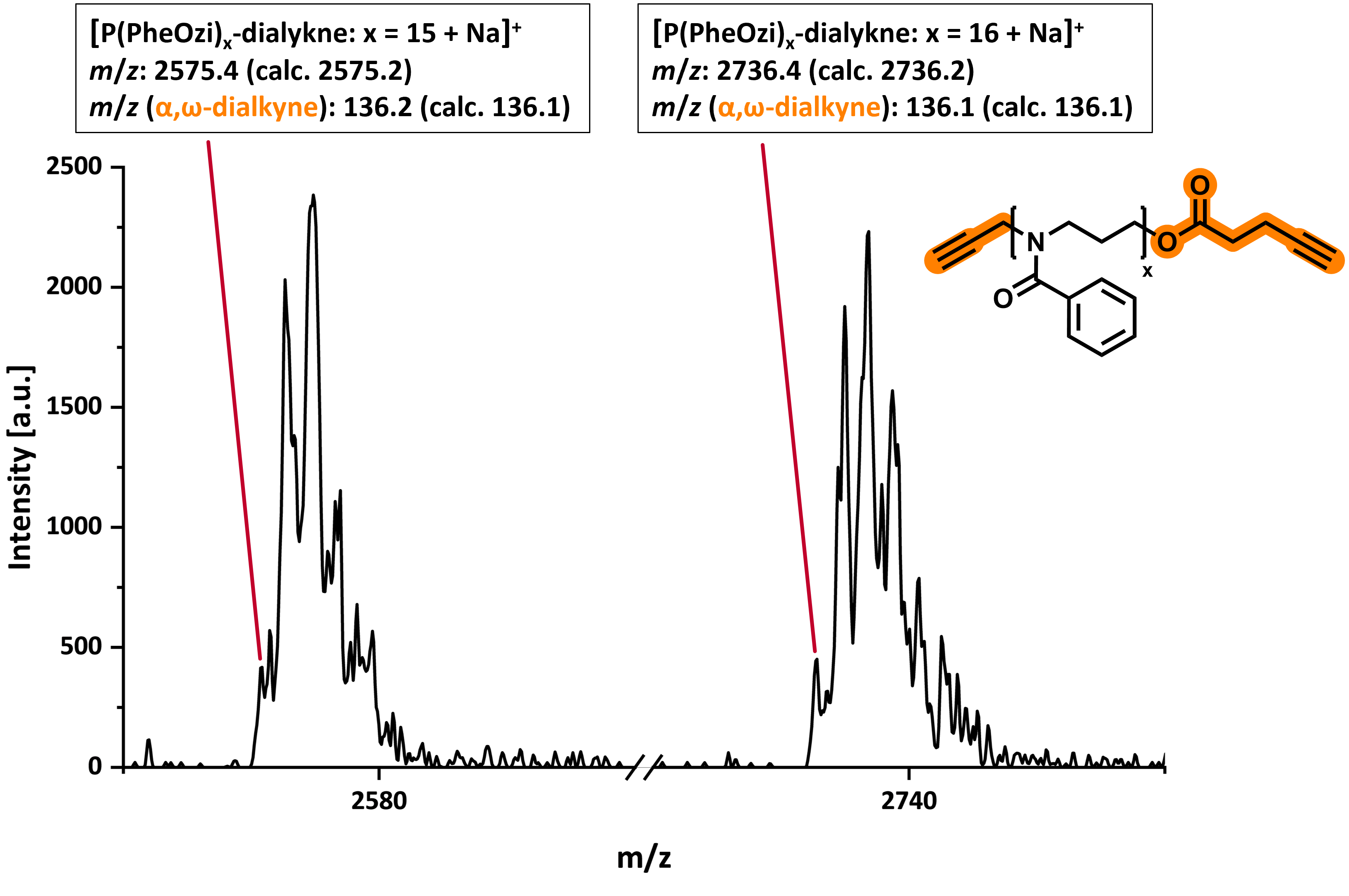


Figure S 34: MALDI-TOF MS (DCTB, NaTFA, reflector modus) zoom in spectra of PPheOzi_16_-dialkyne (Figure 2B). Visualized are the determined and calculated m/z values assigned to α-ω-dialykne structure.

**3.3 SEC Analysis**

**
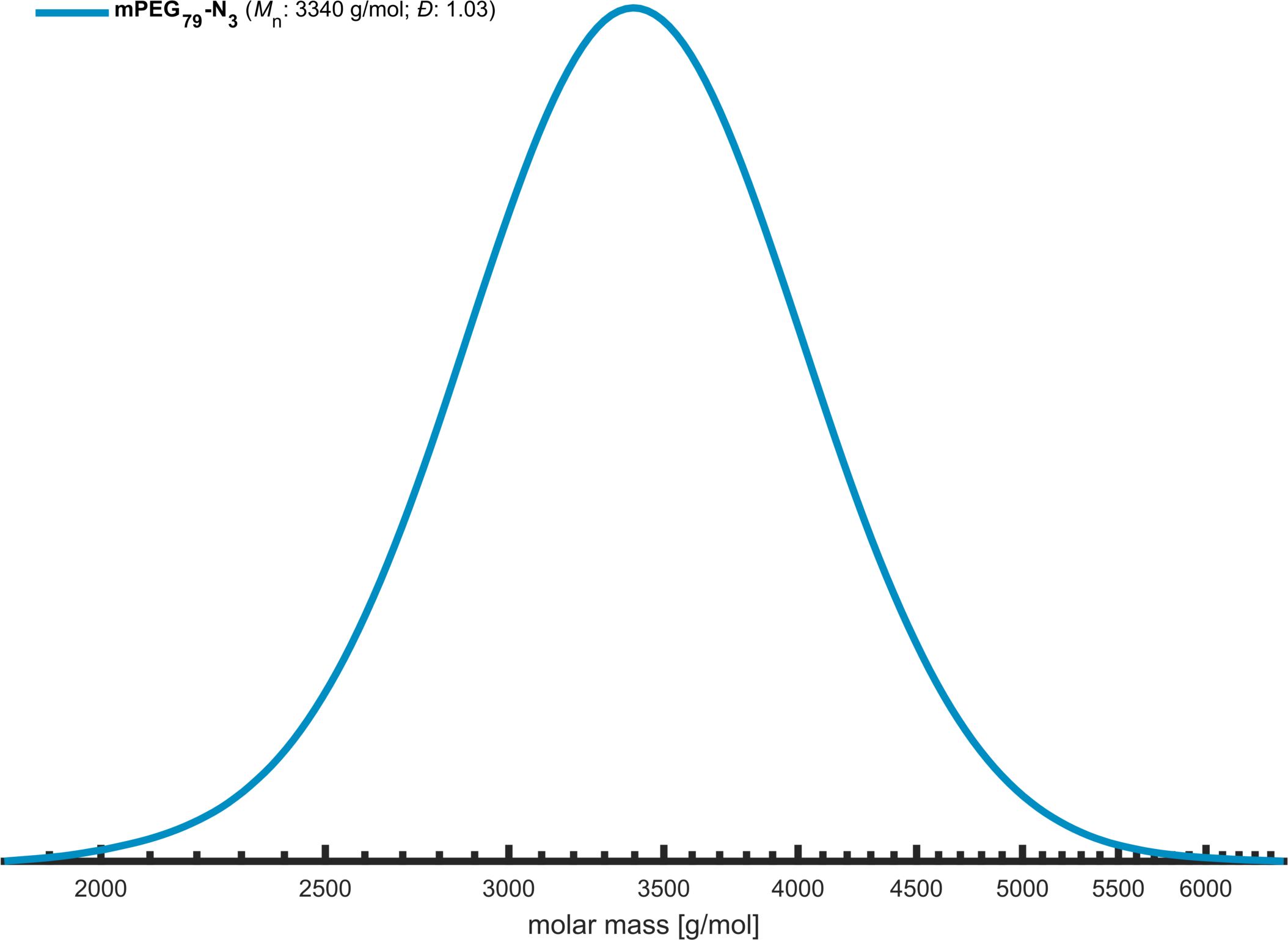
**

Figure S 35: SEC elugrams of the commercially acquired mPEG_79_-N_3_ using DMF + 1 g L^-1^ LiBr as eluent and PEG calibration.

**
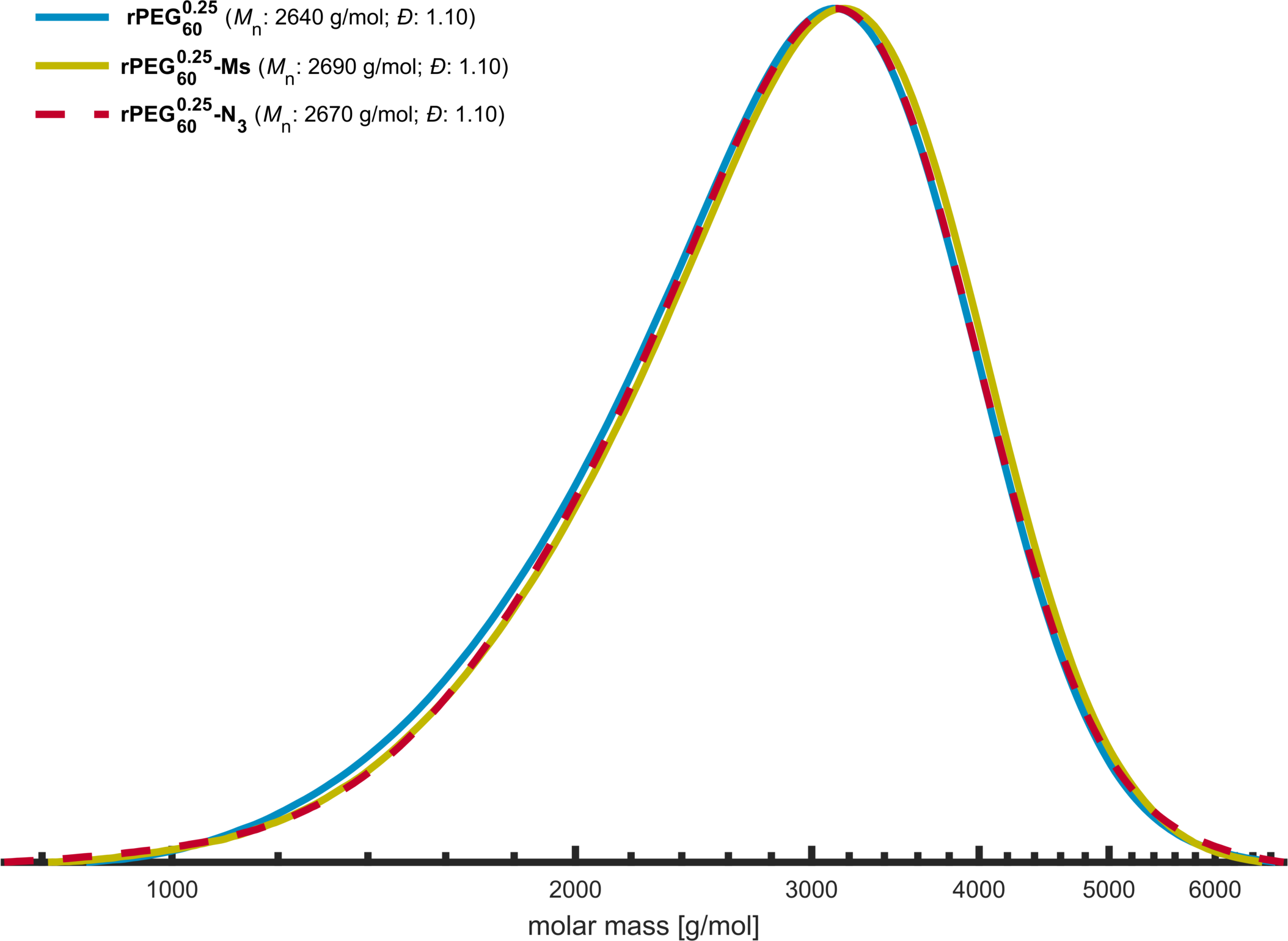
**

Figure S 36: Overlayed SEC elugrams of $\text{rPEG}_{\text{60}}^{\text{0.25}}$ (blue), $\text{rPEG}_{\text{60}}^{\text{0.25}}$-Ms (yellow), and $\text{rPEG}_{\text{58}}^{\text{0.49}}$-N_3_ (red) using DMF + 1 g L^-1^ LiBr as eluent and PEG calibration.


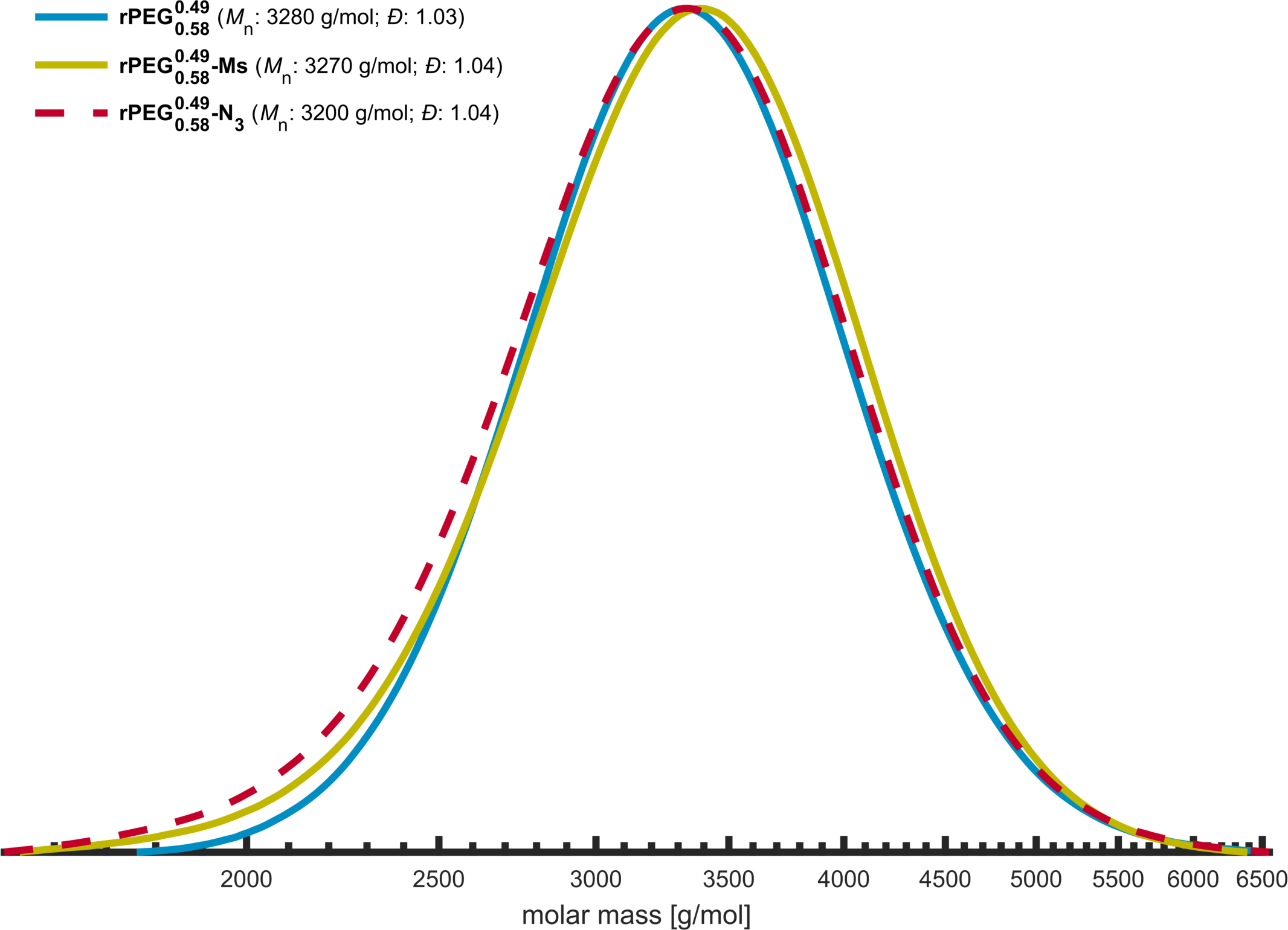


Figure S 37: Overlayed SEC traces of $\text{rPEG}_{\text{58}}^{\text{0.49}}$ (blue), $\text{rPEG}_{\text{58}}^{\text{0.49}}$-Ms (yellow), and $\text{rPEG}_{\text{58}}^{\text{0.49}}$-N_3_ (red) using DMF + 1 g L^-1^ LiBr as eluent and PEG calibration.


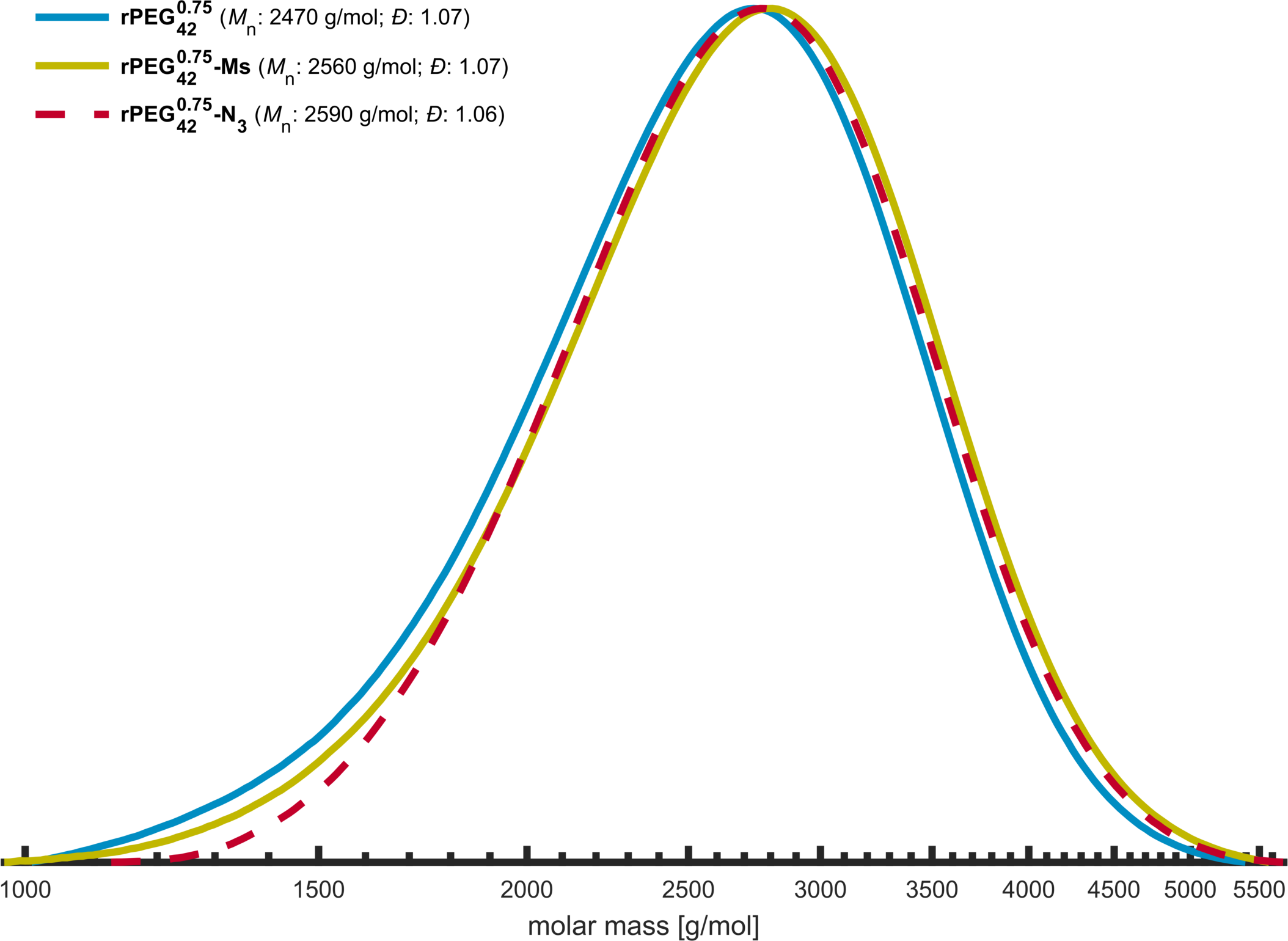


Figure S 38: Overlayed SEC traces of $\text{rPEG}_{\text{42}}^{\text{0.75}}$ (blue), $\text{rPEG}_{\text{42}}^{\text{0.75}}$-Ms (yellow), and $\text{rPEG}_{\text{42}}^{\text{0.75}}$-N_3_ (red) using DMF + 1 g L^-1^ LiBr as eluent and PEG calibration.


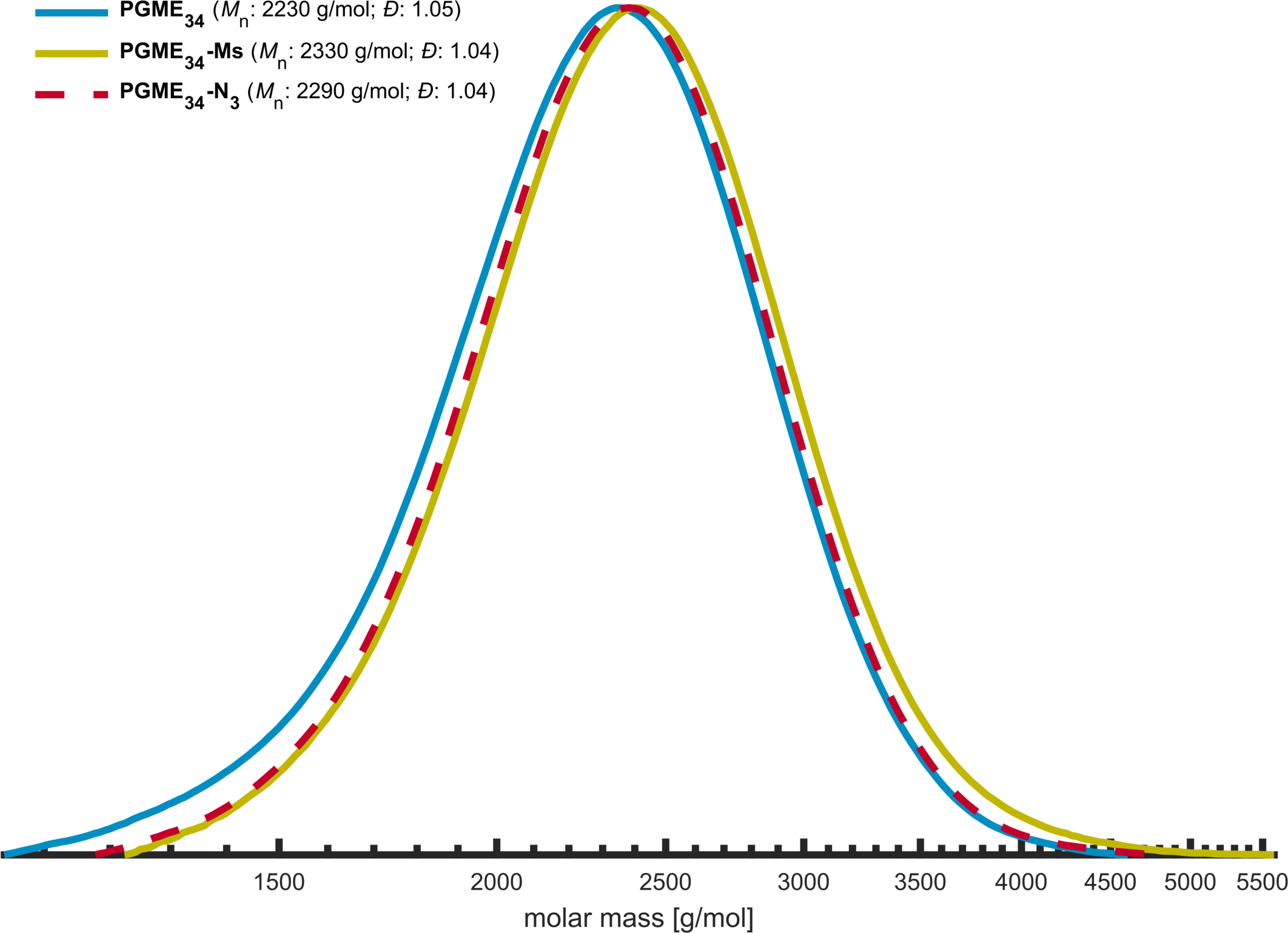


Figure S 39: Overlayed SEC traces of PGME_34_ (blue), PGME_34_-Ms (yellow) and PGME_34_-N_3_ (red) using DMF + 1 g L^-1^ LiBr as eluent and PEG calibration.

**3.4 Efavirenz Calibration**


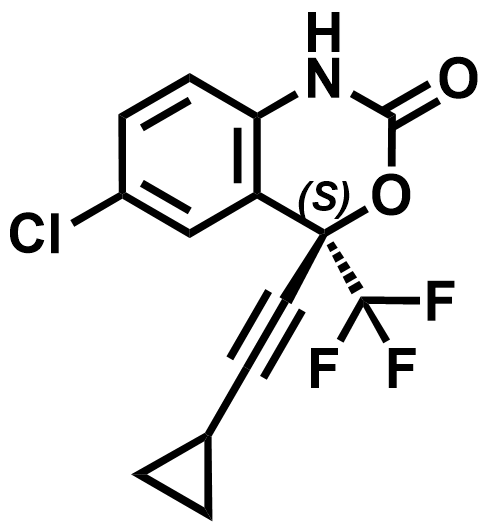


Figure S 40: Structure of Efavirenz (EFV, IUPAC: (4S)-6-Chlor-4-(2-cyclopropylethinyl)-4-(trifluormethyl)-2H-3,1-benzoxazin-2-on).


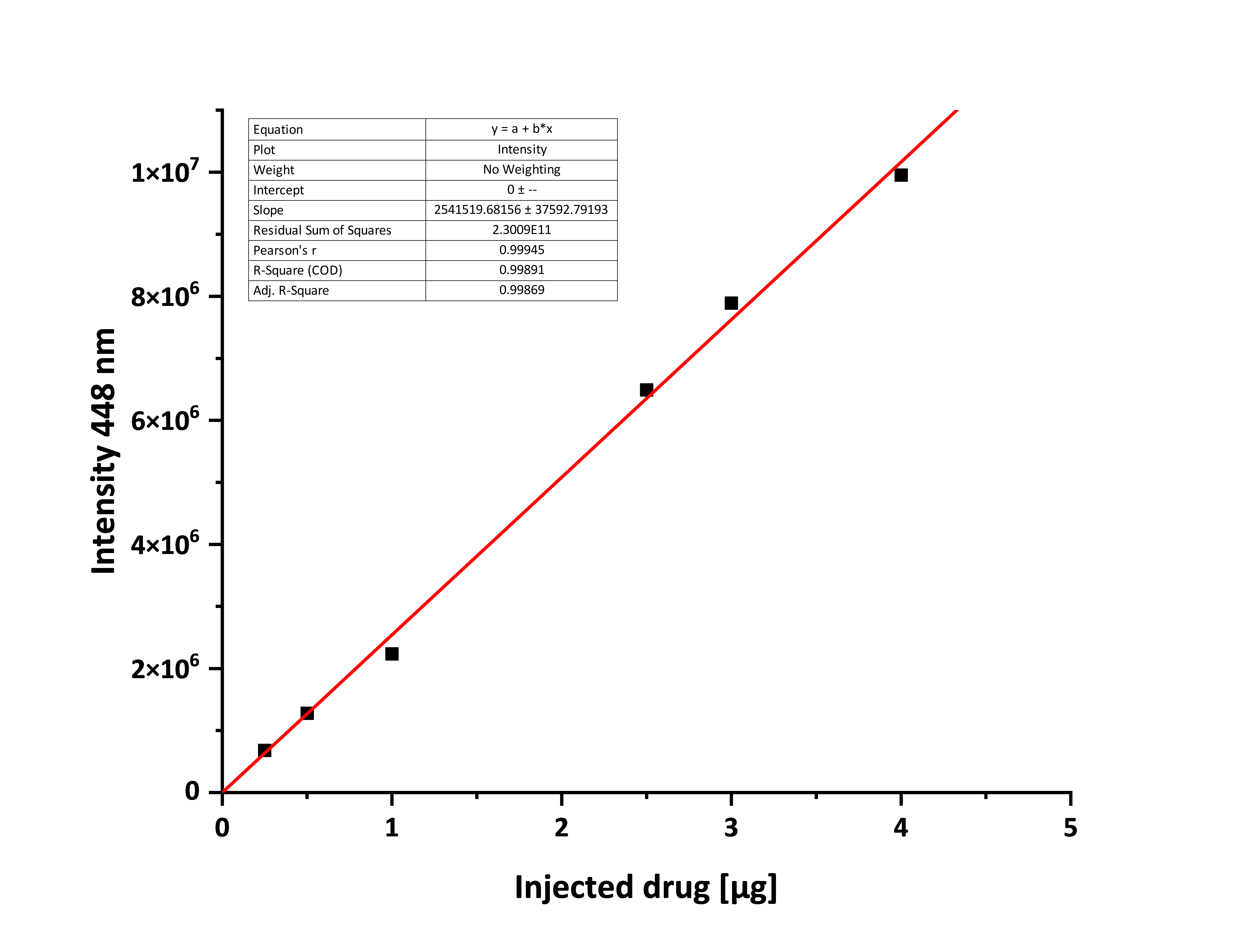


Figure S 41: Efavirenz calibration. 50 µL of Efavirenz standards in the concentration range of 5 to 80 mg L^-1^ were injected. The linear regression was performed with forced zero-intercept without weighting in Origin2024Pro.

**3.5 Flow Cytometry**


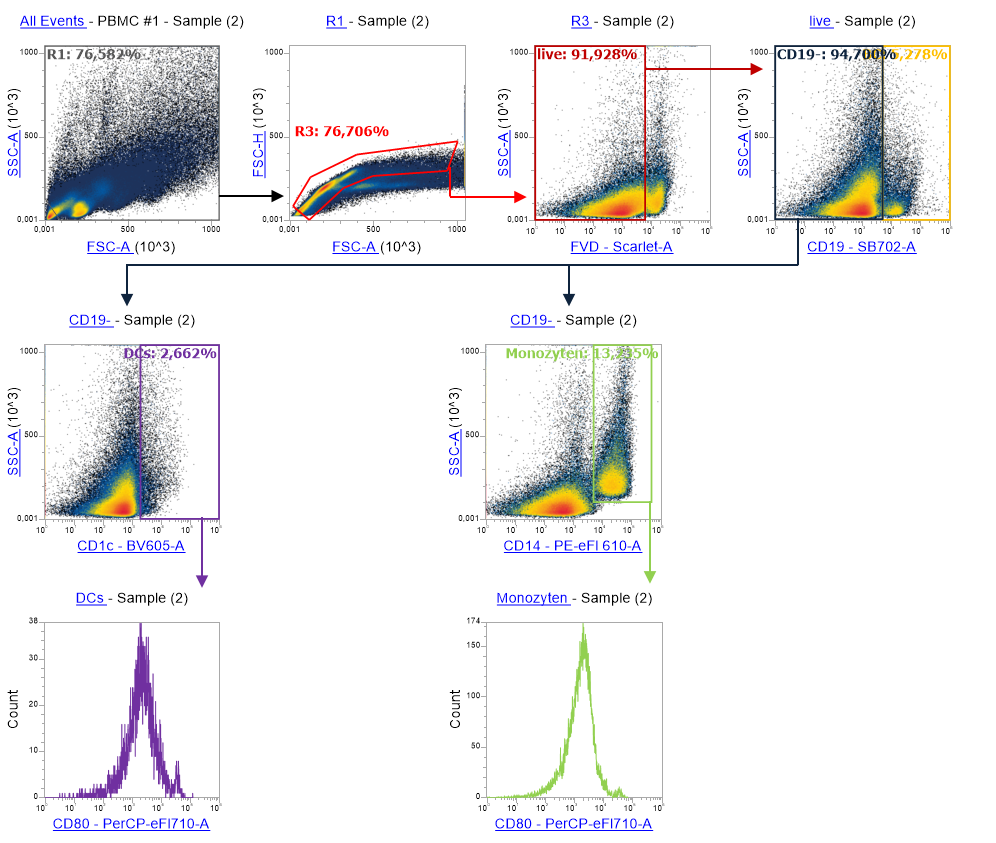


Figure S 42: Gating strategy of PMBC flow cytometry analysis to delineate cell types and corresponding immune activation.


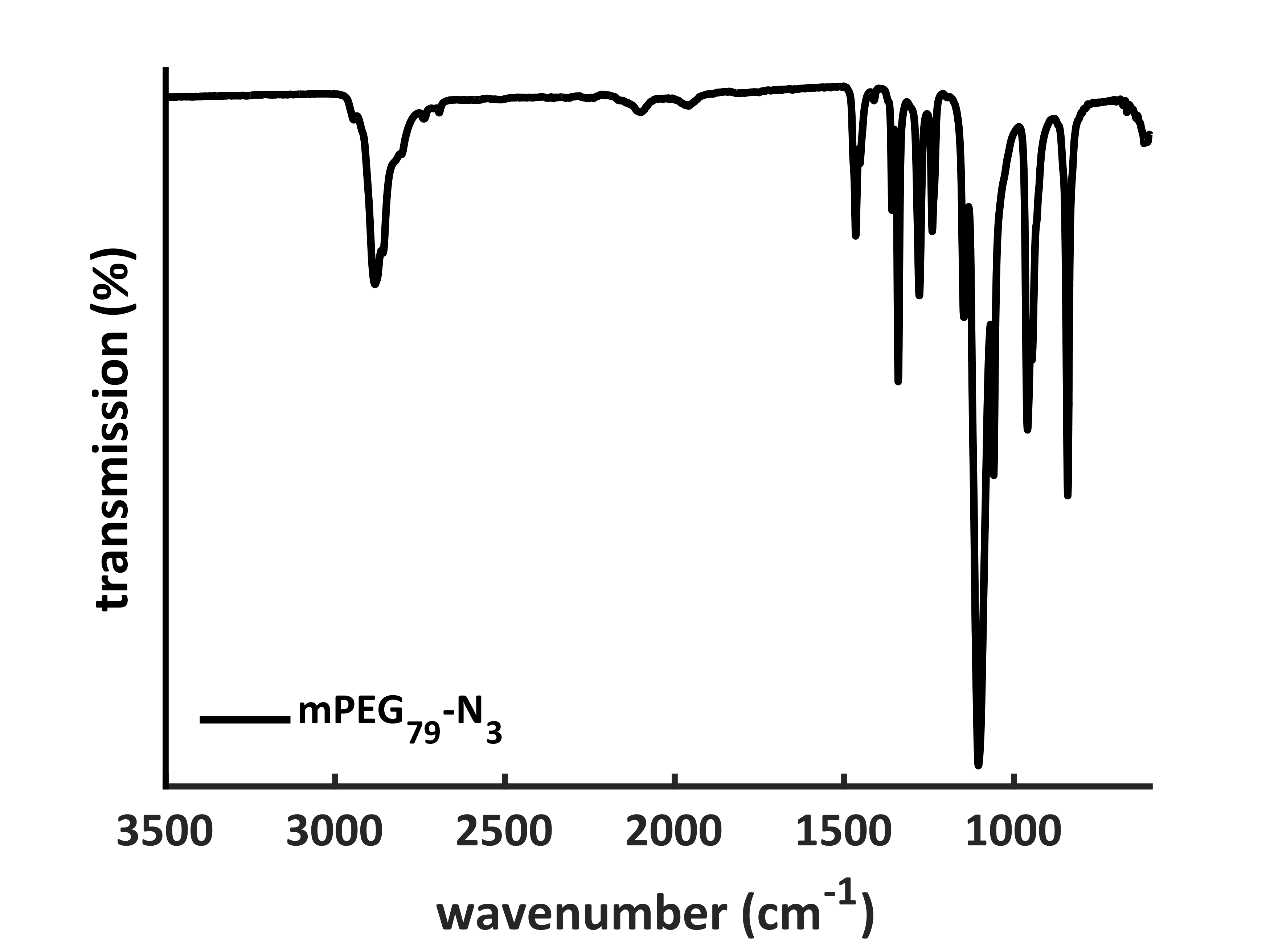


Figure S 43: FTIR spectra of mPEG_79_-N_3_ showing the characteristic azide bands (2200 – 2000 cm^-1^).


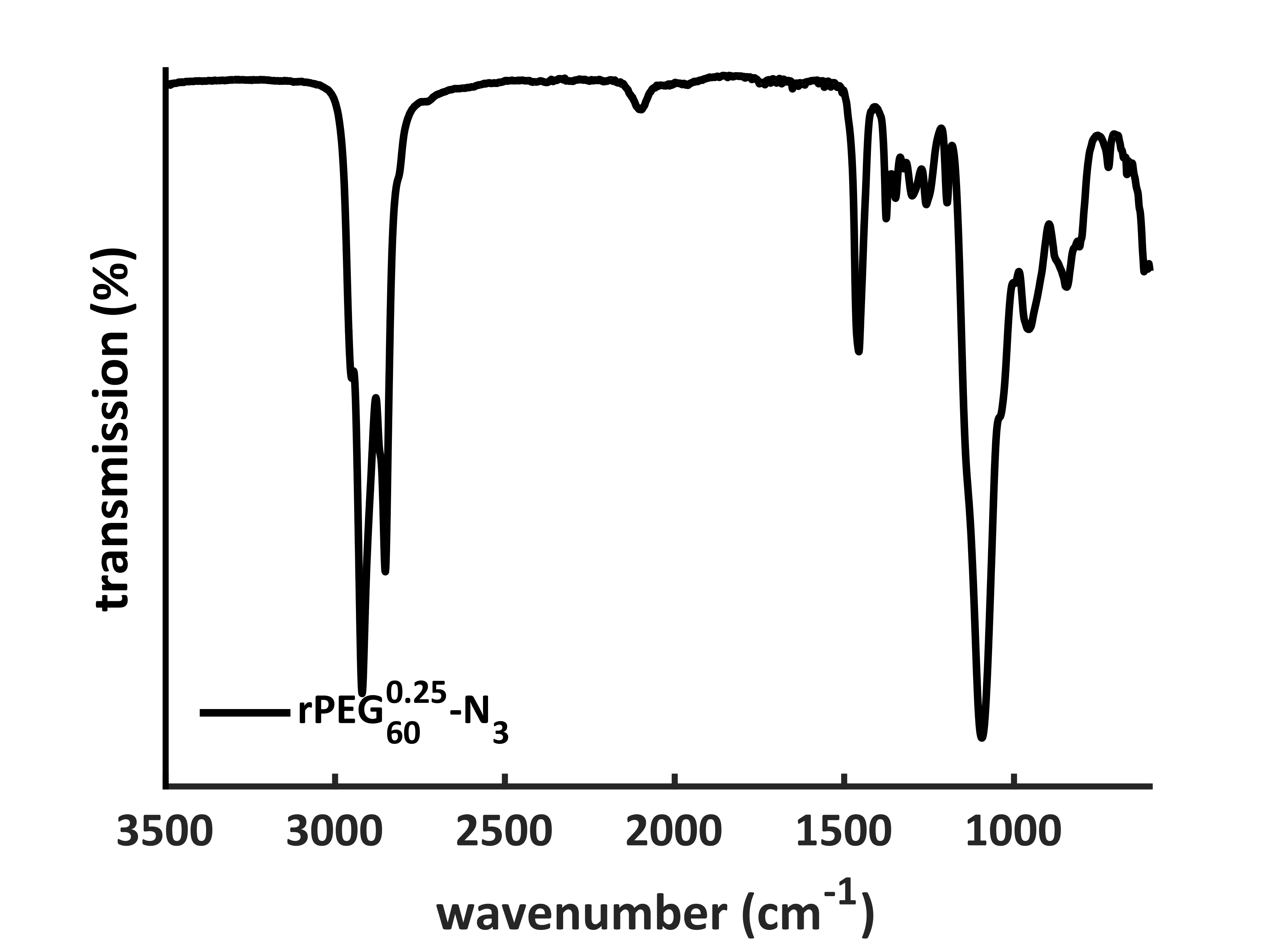


Figure S 44: FTIR spectra of $\text{rPEG}_{\text{60}}^{\text{0.25}}$-N_3_ showing the characteristic azide bands (2200 – 2000 cm^-1^).


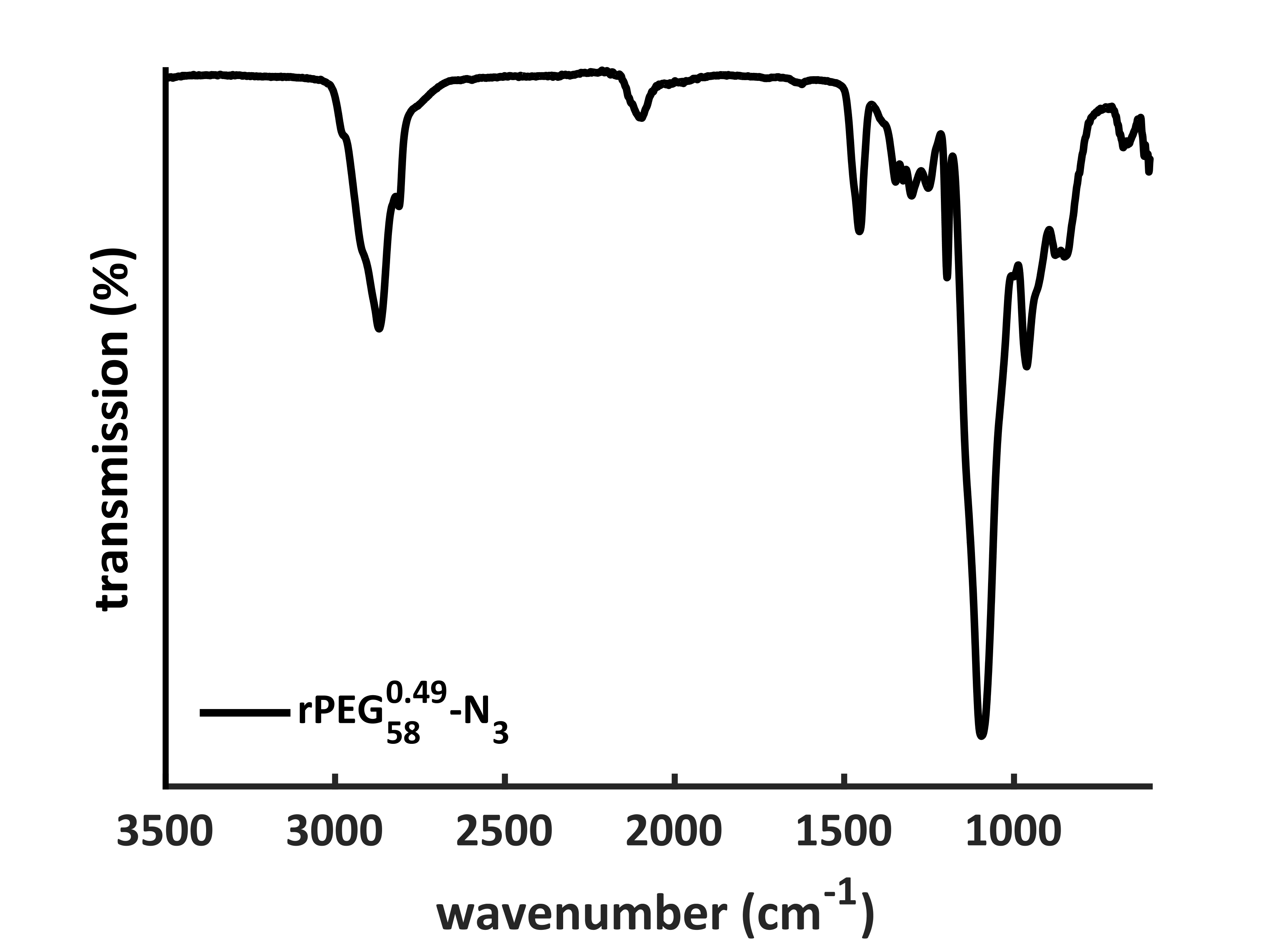


Figure S 45: FTIR spectra of $\text{rPEG}_{\text{58}}^{\text{0.49}}$-N_3_ showing the characteristic azide bands (2200 – 2000 cm^-1^).


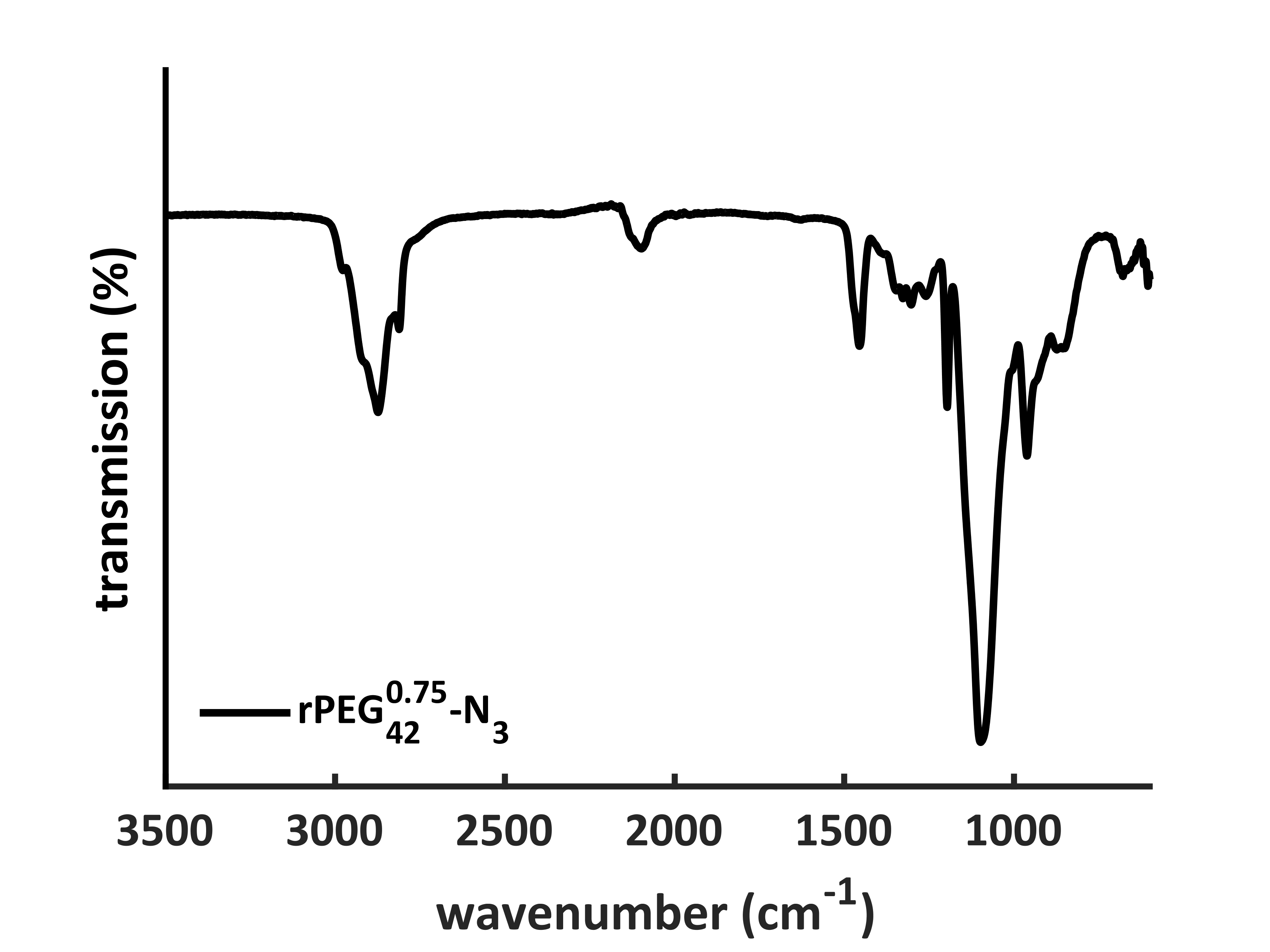


Figure S 46: FTIR spectra of $\text{rPEG}_{\text{42}}^{\text{0.75}}$-N_3_ showing the characteristic azide bands (2200 – 2000 cm^-1^).


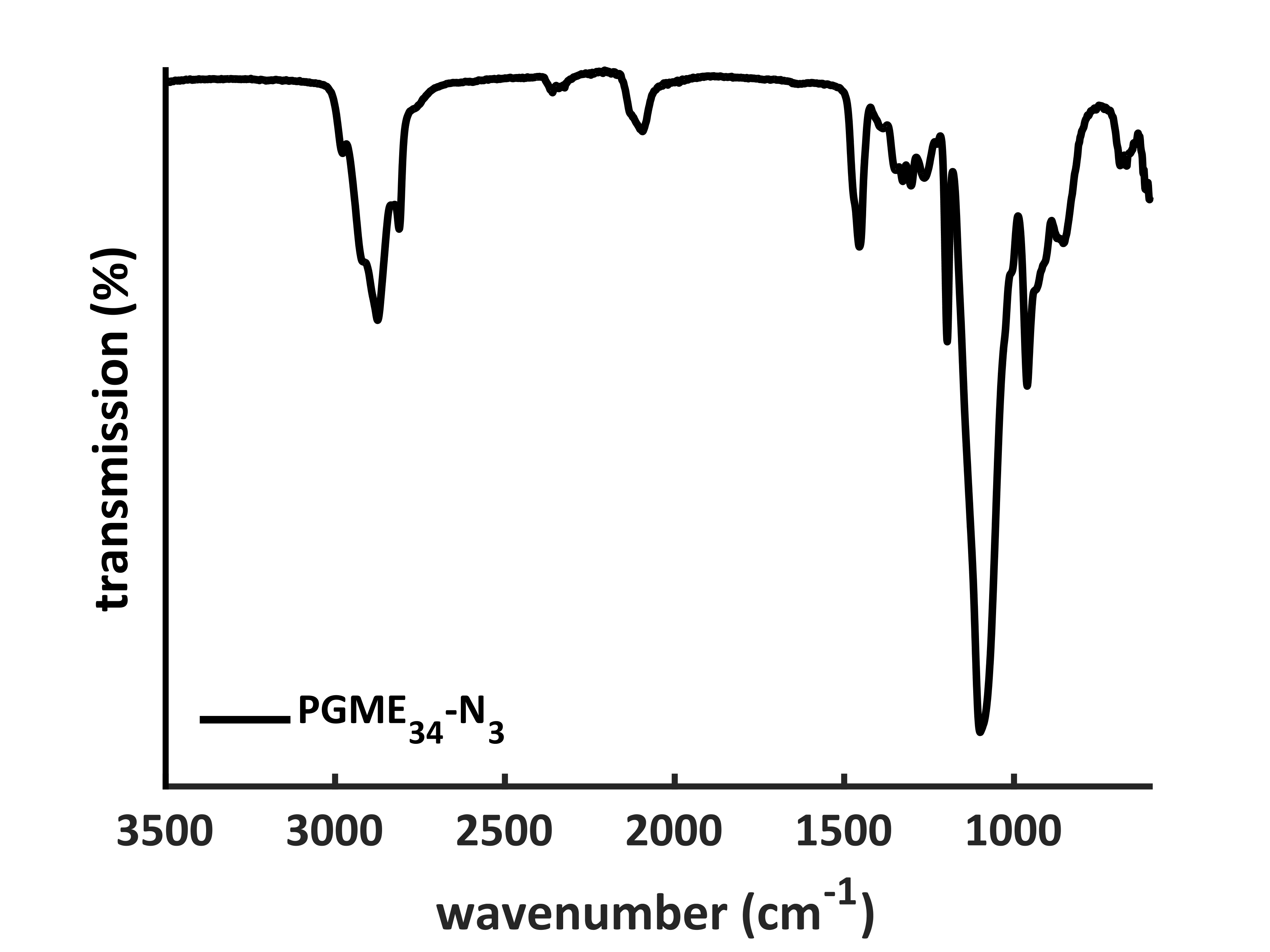


Figure S 47: FTIR spectra of PGME_34_-N_3_ showing the characteristic azide bands (2200 – 2000 cm^-1^).


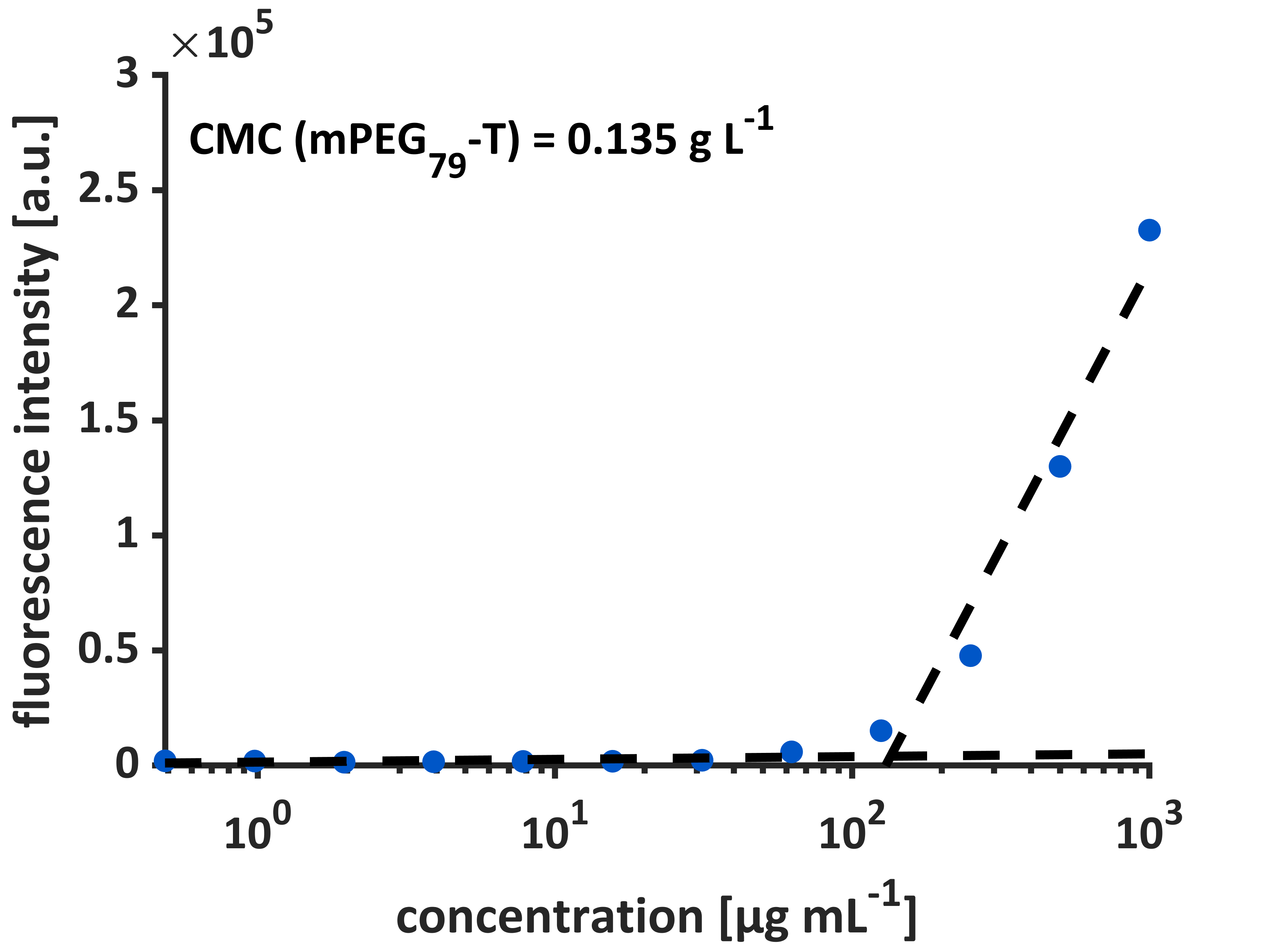


Figure S 48: Determination of the critical micelle concentration of mPEG_79_-T using coumarin-6 method.


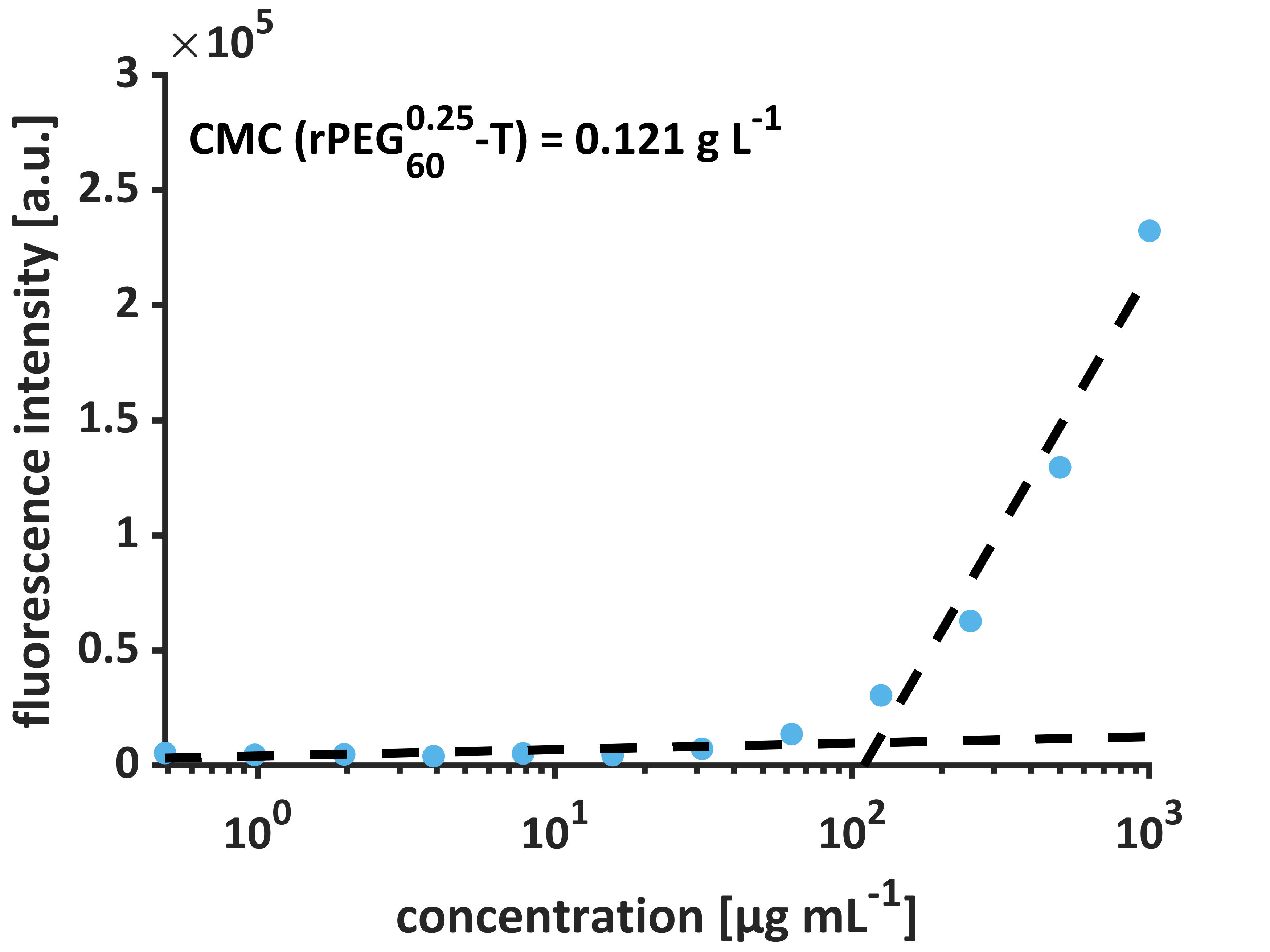


Figure S 49: Determination of the critical micelle concentration of $\text{rPEG}_{\text{60}}^{\text{0.25}}$-**T** using coumarin-6 method.


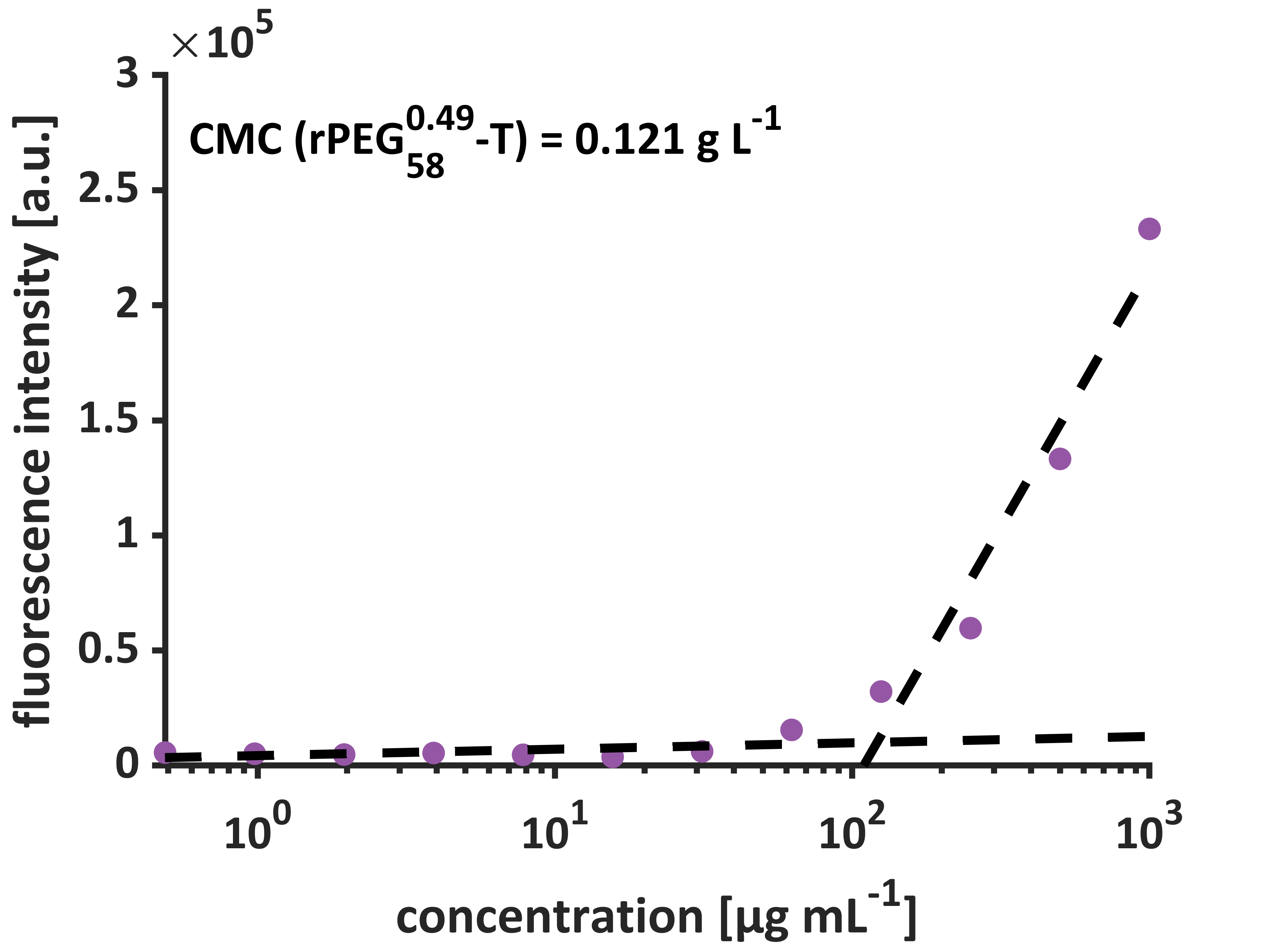


Figure S 50: Determination of the critical micelle concentration of $\text{rPEG}_{\text{58}}^{\text{0.49}}$-**T** using coumarin-6 method.


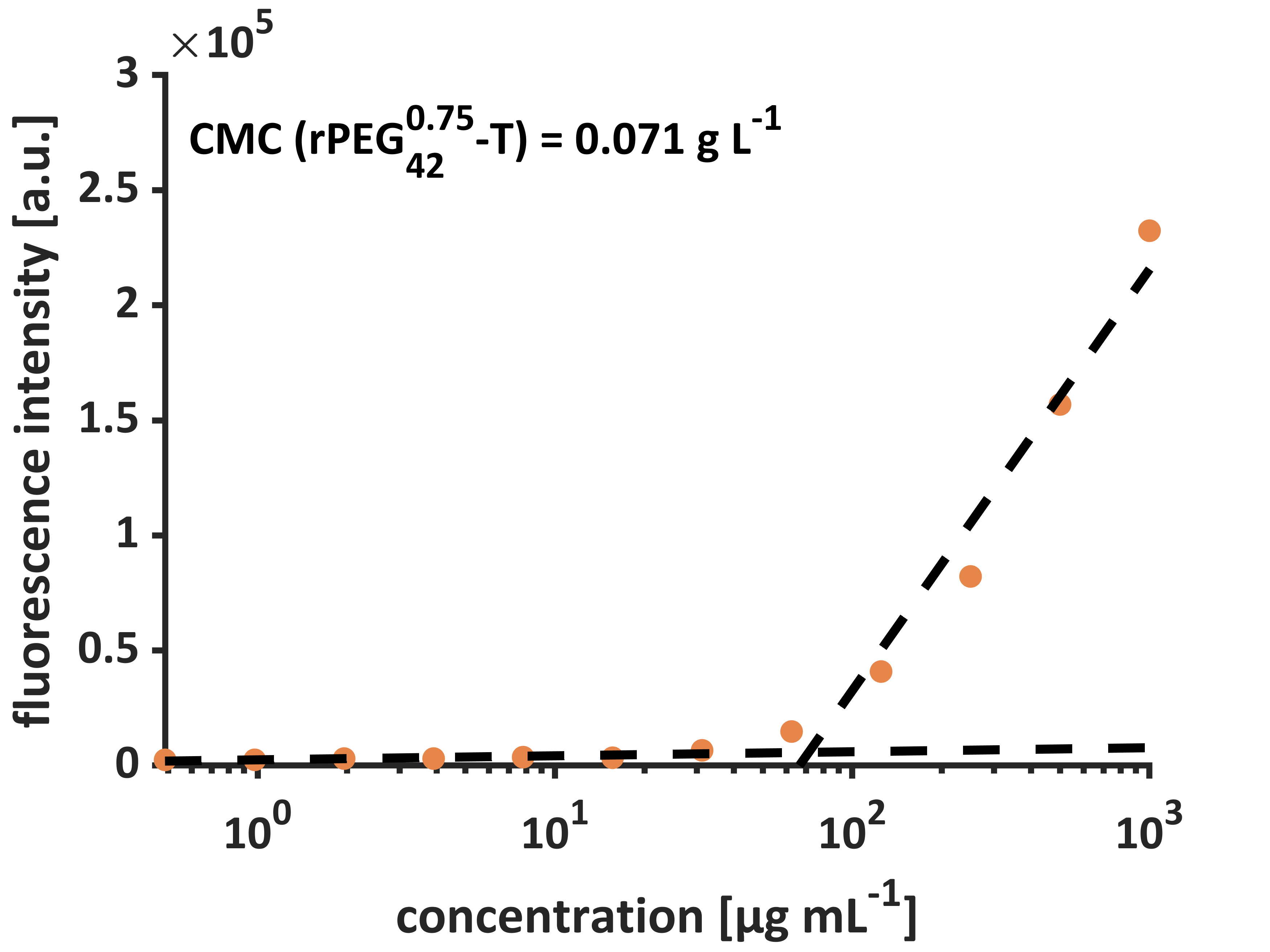


Figure S 51: Determination of the critical micelle concentration of $\text{rPEG}_{\text{42}}^{\text{0.75}}$-**T** using coumarin-6 method.


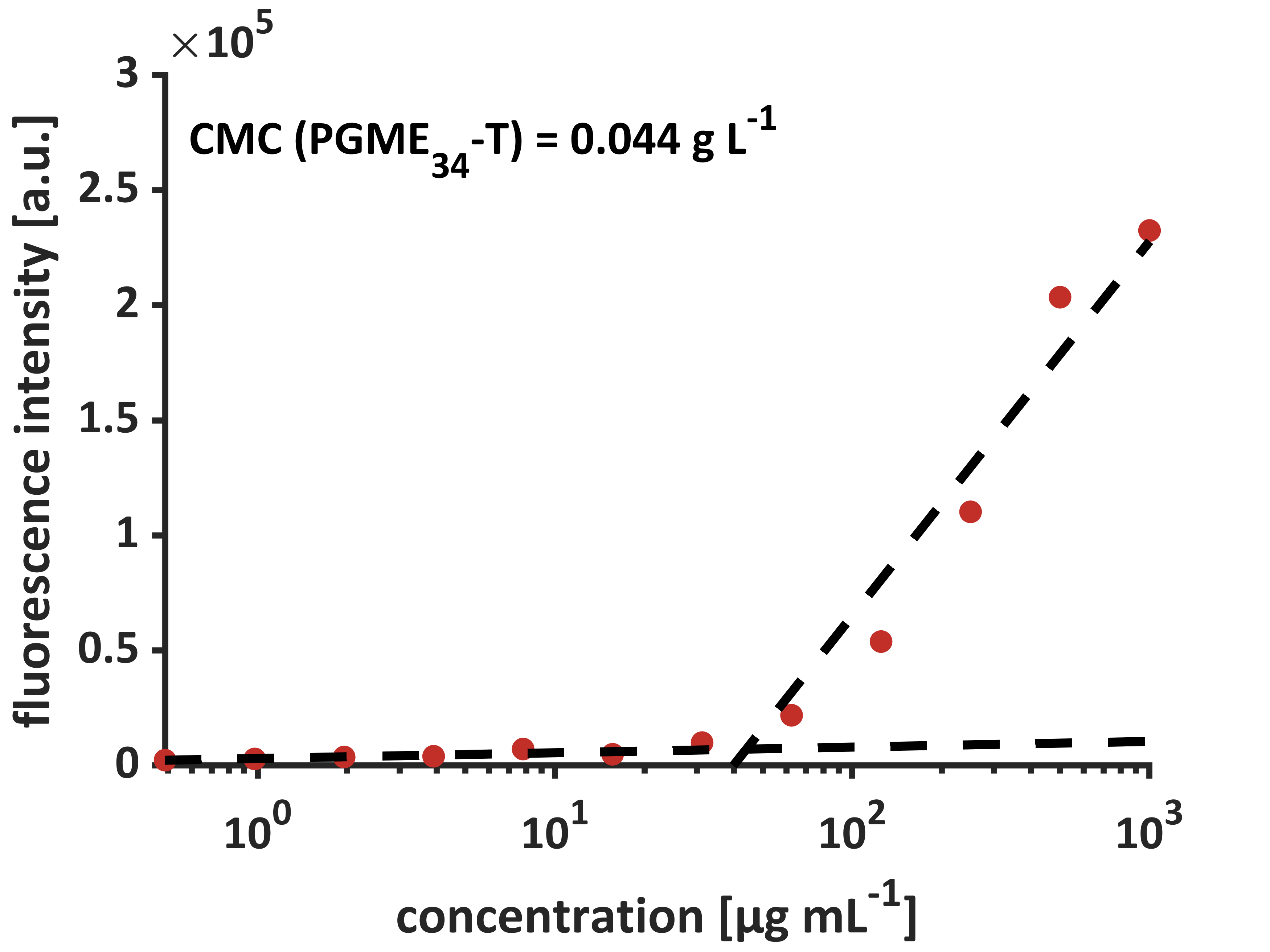


Figure S 52: Determination of the critical micelle concentration of PGME_34_-**T** using coumarin-6 method.


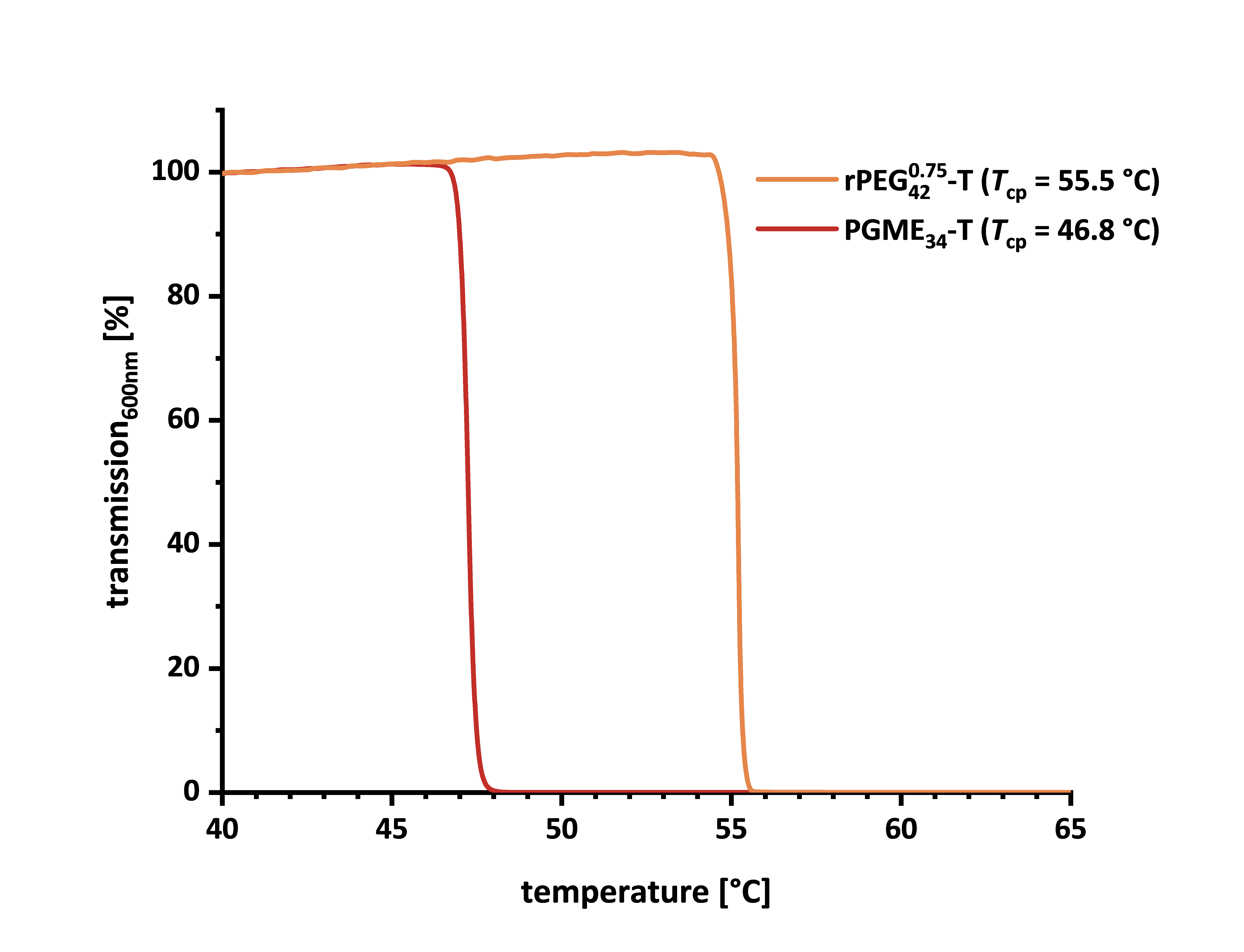


Figure S 53: Turbidimetry analysis (heating curve, 10 mg mL^-1^) of $\text{rPEG}_{\text{42}}^{\text{0.75}}$-**T** and PGME_34_-**T** in deionized water. The cloud points (T_cp_) are assigned as 50% decrease in transmission at 600 nm.

**References**

[1] P. Dreier, R. Matthes, F. Fuß, et al., “Isomerization of Poly(ethylene glycol): A Strategy for the Evasion of Anti-PEG Antibody Recognition”, *Journal of the American Chemical Society.* **2025.** 10.1021/jacs.5c02716.

[2] S. M. E. Vrouenraets, F. W. N. M. Wit, J. van Tongeren, J. M. A. Lange, “Efavirenz: a review”, *Expert Opinion on Pharmacotherapy.* **2007**, *8*, 851. 10.1517/14656566.8.6.851.

[3] A. Fluksman, O. Benny, “A robust method for critical micelle concentration determination using coumarin-6 as a fluorescent probe”, *Analytical Methods.* **2019**, *11*, 3810. 10.1039/C9AY00577C.

[4] B.-M. Chen, Y.-C. Su, C.-J. Chang, et al., “Measurement of Pre-Existing IgG and IgM Antibodies against Polyethylene Glycol in Healthy Individuals”, *Analytical Chemistry.* **2016**, *88*, 10661. 10.1021/acs.analchem.6b03109.
